# Sight-over-sound effect depends on interaction between evaluators’ musical experience and auditory-visual integration: An examination using Japanese brass band competition recordings

**DOI:** 10.1371/journal.pone.0321442

**Published:** 2025-04-29

**Authors:** Tomohiro Samma, Kazuaki Honda, Shinya Fujii

**Affiliations:** 1 Graduate School of Media and Governance, Keio University, Fujisawa, Japan; 2 NTT Communication Science Laboratories, NTT Corporation, Atsugi, Japan; 3 Faculty of Environment and Information Studies, Keio University, Fujisawa, Japan; Federal University of Paraiba, BRAZIL

## Abstract

The sight-over-sound effect, in which visual information dominates auditory cues in musical evaluations, challenges the common belief that sound is the primary factor in music evaluation. However, the replicability of the sight-over-sound effect remains controversial across different musical genres and contexts. Here, we investigated the sight-over-sound effect using recordings from Japanese brass band competitions with carefully controlled musical pieces and camera angles. Adult participants (age: 34.81 ± 11.71 years [mean ± standard deviation]) were divided into three groups based on their brass band and musical experience. Of the 301 participants, 171 were brass band musicians (age: 33.08 ± 11.57 years), 78 were non-brass band musicians (age: 35.39 ± 13.03 years), and 52 were non-musicians (age: 39.15 ± 8.73 years). The sight-over-sound effect was observed in non-brass band musicians (Kruskal–Wallis test: *p* < 0.001) but was absent in brass band musicians (*p* = 0.48) and non-musicians (*p* = 0.37). These findings indicate that the sight-over-sound effect depends on musical experience; specifically, our results indicate that auditory training in brass band musicians may mitigate the visual influence. Furthermore, the absence of the sight-over-sound effect in non-musicians suggests that without specific musical training, evaluators may not consistently prioritize visual information. The present findings fill a critical gap in our understanding of multisensory integration, especially regarding how different levels of musical expertise shape evaluative processes across sensory modalities. Our results underscore the need for educational and evaluative practices to consciously balance the influences of visual and auditory cues, particularly in situations in which visual dominance may overshadow auditory quality.

## Introduction

It is generally believed that auditory information is central to the domain of music and that auditory (rather than visual) information is a crucial element when judging musical performance [1]. However, in contrast to this belief, numerous studies have demonstrated the importance of visual information in musical performance [2–4]. Visual information plays an important role in appreciating performer expressiveness [5–7], aesthetic experience [8,9], attractiveness, and stage behavior [10–14]. For example, Behne and Wöllner [15] reported that performance evaluations can be altered by manipulations of the visual video, despite the presentation of identical sounds in the videos. In addition, other studies have reported that ancillary gestures that do not directly affect the performance can influence musical expression [16–20]. Investigations have also demonstrated that the length of a hitting motion influences the perceived duration of a percussion sound [21,22]. Furthermore, visual expressions by conductors reportedly affect the audience’s evaluation of expressiveness [23,24], and performances by more expressive conductors receive higher evaluations [25,26]. Furthermore, a recent interview study that targeted musicians noted that musicians themselves recognize that visual information (such as body movements and behaviors) is an important element of musical performance [27,28]. Together, these studies suggest that visual information markedly affects the evaluation of musical performance.

A recent study examined the relative influence of visual and auditory information in the evaluation of musical performances [1]. Regardless of musical experience, people consistently reported that auditory information was more important than visual information when evaluating musical performances (see Experiment 1 in [1]). In Experiment 3 in [1], participants watched 6-s excerpts of recordings from the first, second, and third-place performers in international music competitions and were asked to select the actual winner (chance level: 33.3%). Non-musicians correctly identified the actual winner most accurately when they made evaluations based solely on visual-only (VO) stimuli (mean answering rate: 46.4%) compared with evaluations based on audio-only (AO; 28.8%) or audio-visual (AV; 35.4%) stimuli. Similarly, professional musicians were able to select the winner with higher accuracy when evaluations were based on VO stimuli (47.0%) compared with AO (25.7%) and AV (29.5%; Experiment 5 in [1]) stimuli. These findings were termed the “sight-over-sound effect” [1]. Together, the results indicate that although sound is acknowledged as the most crucial source of information in the evaluation of musical performances, a natural and automatic reliance on visual cues also occurs subconsciously [1].

Since the publication of the study by Tsay [1], the replicability of the sight-over-sound effect has been extensively debated [1,29–33]. Mehr et al. [30] used the same stimuli presented by Tsay [1] and demonstrated that the sight-over-sound effect is replicable. However, when the experiment was modified to a two-choice format (chance level: 50%) using a combination of performance stimuli from the winner and a preliminary round loser, the sight-over-sound effect was not observed (VO: 45.2%, AO: 68.4%, AV: 63.6%; Experiment 3 in [30]). These results suggest that the sight-over-sound effect may not be replicable when differences in the skill levels of the performances being compared can be adequately judged through auditory perception alone. Thus, the sight-over-sound effect remains a subject of debate, with ongoing investigations into its replicability and adaptability across musical genres [31] and performance styles (e.g., solo vs. group performances) [29,33]. In summary, although there is a subjective perception that musical performances should be evaluated with an emphasis on sound, it is conceivable that different winners may be chosen based on the availability of visual information if the sight-over-sound effect indeed applies to actual competitions—especially when the skill levels of the compared performers are close. The potential existence of the sight-over-sound effect in competitions suggests that performers should be mindful that the judgments of evaluator can be substantially influenced by performers’ visual cues and preferences, necessitating training not only in the musical aspects of performance but also in visually engaging the evaluators. Moreover, the sight-over-sound effect may overturn the traditional concept of music as an “art of sound.”

In this context, we propose that there are at least three issues that should be considered when testing the replicability of the sight-over-sound effect. First, the stimuli used and compared in previous studies exhibit differences. Mehr et al. [30] also identified problems in the studies by Tsay [1,33], noting that the information in the stimuli that was used was not controlled. Specifically, these authors highlighted differences in camera angles and varying degrees of exposure to the outfit of each performer. Mehr et al. [30] hypothesized that controlling such visual information might prevent the replicability of the sight-over-sound effect. By examining this issue, it might be possible to clarify whether the sight-over-sound effect arises from variations in visual information, such as differences in camera angles and performer attire.

Second, the musical pieces used in the stimuli being compared have not always been well controlled. Mehr et al. [30] hypothesized that the sight-over-sound effect would not be replicated if the degree of movement of each performer was matched. By controlling the musical pieces, both the necessary movements for playing instruments and the ancillary movements for expression would be more consistent, thereby allowing this prediction to be tested. However, no previous research has controlled the musical pieces that are being compared. Recent research has indicated that the congruence between performers and musical pieces can influence musical performance evaluations [34]. Based on this research, the congruence or incongruence between the visual information from the performers and the auditory information from the musical piece might influence musical performance judgments. Thus, to verify the replicability of the sight-over-sound effect, it is crucial to control the musical pieces in the compared stimuli.

Third, the detailed musical experience of each evaluator should be considered when examining the sight-over-sound effect. Although previous studies have revealed that this effect occurs regardless of musical experience, the way in which musical expertise has been assessed varies. For example, Tsay [1] used professional orchestra musicians to evaluate piano performances, whereas Mehr et al. [30] identified participants with high Musical Ear Test [35] scores as having high musical expertise, despite the lack of a group with extensive musical training. A key issue is the mismatch between the musical expertise of the evaluators and the genre of the performance that is being assessed. Research indicates that long-term music training leads to brain changes that are specific to the instrument played, thereby affecting cognitive functions and skills [36–39]. Therefore, even within a group of evaluators with a background in long-term music training, experiences with different instruments or genres may influence performance preferences and evaluations. In addition, individuals with musical experience reportedly possess advanced auditory processing abilities [40,41], with higher pitch [42–44] and rhythm perception [45–47] accuracy than non-musicians. Such auditory information processing, developed through musical training, may influence the sight-over-sound effect. The musical backgrounds of evaluators may also affect how they perceive the visual aspects of performances. Waddell and Williamon [13] reported that musicians are harsher in their ratings than non-musicians, particularly toward performers with poor entrance attitudes or negative facial expressions during mistakes. Together, these findings highlight the need to consider musical experience when studying the sight-over-sound effect.

Given the three aforementioned issues in previous research into the sight-over-sound effect, we aimed to investigate the replicability of the sight-over-sound effect in the judgment of Japanese brass band competitions under controlled conditions using participants with both genre-specific and -nonspecific musical experience. In Japanese brass band competitions, all participating bands choose one piece to perform from a small set of assigned pieces; this enabled us to address the issue of controlling the camera angle and musical piece, which remained unresolved in previous studies. Additionally, we compared the choice of brass band winners not only between musicians and non-musicians (NMs), but also between brass band musicians (BMs) and non-brass band musicians (NBMs). This allowed us to comprehensively investigate the replicability of the sight-over-sound effect when musical expertise matches the stimulus genre.

We formulated two hypotheses in the present study: 1) that the sight-over-sound effect is not replicable in the judgment of Japanese brass band competitions when camera angles and music pieces are controlled; and 2) that BMs do not exhibit the sight-over-sound effect because of their ability to precisely evaluate the auditory information and accurately predict the winners.

The first hypothesis, which was based on Mehr et al. [30], predicts that the sight-over-sound effect will not be replicated when controlling for stimulus information such as camera angles and musical pieces. Previous studies have reported that VO conditions yield accuracy rates that are significantly above chance. However, with the controlled camera angles and musical pieces used in the current study, we hypothesized that the reduced variability in visual information may lead to lower accuracy rates in VO conditions. For this part of the investigation, we aimed to explore whether the sight-over-sound effect occurs when participants choose winners, regardless of their brass band or musical experience.

The second hypothesis suggests that, because of their specific musical experience, BMs might select the actual winners more accurately in AO and AV conditions than in VO conditions. A previous study demonstrated that musicians show greater selective attention to auditory information than non-musicians [48], and musical training reportedly enhances the development of selective auditory attention [49]. Because brass band performance is an ensemble involving multiple players, BMs need to be able to listen to their own sound while selectively distinguishing the sounds of others. It is therefore likely that individuals with brass band experience have better selective auditory attention abilities than NMs or NBMs. As a result, BMs may be able to leverage their brass band experience to accurately distinguish sounds and make more precise evaluations even when relying solely on auditory information. Unlike previous studies with inconsistent pieces, the consistent musical pieces used in the present study may be helpful for sound-based evaluations. This may allow BMs to use their expertise to evaluate sound and predict winners, thereby potentially negating the sight-over-sound effect.

## Methods

### Participant recruitment

The experimental protocol was approved by the Ethics Committee at Keio University Shonan Fujisawa Campus on September 19, 2019 (No. 256). Inclusion criteria were as follows: 1) aged 18 and over, 2) native Japanese speakers, and 3) without hearing or visual impairments. Participants were recruited from Keio University and its surrounding areas using a combination of social media and word-of-mouth advertising. We endeavored to ensure a balanced representation of individuals with brass band and musical experience by broadly soliciting participants. Informed consent was obtained from all participants via an online survey system as follows. The experimental instructions in the protocol were displayed on the screen; participants were required to fully comprehend the content of the instructions and provide consent for their involvement in the experiment by selecting the “Agree to Participate” button if they voluntarily agreed to participate. The demographic data (including age, sex, musical background, and brass band experience) collected through the questionnaire were handled in an anonymized manner by assigning a unique identifier to each participant. The recruitment period was from December 10, 2019, to June 30, 2020.

### Statistical power

We conducted a power analysis to determine an adequate sample size for testing our hypotheses. The effect size was estimated using G*Power [50] based on Experiment 1 from Mehr et al. [30], with an expected effect size of Cohen’s *d* = 0.23. The significance level was set to *α* = 0.05, and the power was set at 0.95. The power analysis indicated that 285 participants (95 per group) would be required.

### Participant grouping

The participants were asked if they had any musical training experience other than mandatory music education in schools or other educational institutions. If they had musical training experience, they were then asked if they had specific experience of playing in brass bands. We also asked the participants about the musical instruments they had practiced, the age of commencement, and the duration of musical training. To specifically explore the effects of experience with brass band music, we categorized participants into three groups: BMs, NBMs, and NMs. The participants were grouped based on their familiarity with brass band performances because we hypothesized that this might influence their evaluations. BMs had direct brass band experience, providing genre-specific expertise. By contrast, NBMs were musicians with general musical expertise but no brass band experience, and NMs had no formal musical training beyond compulsory education. These groupings were designed to examine whether familiarity with brass bands—beyond general musical experience—affects the judgment of brass band performances and contributes to the sight-over-sound effect.

### Stimuli

We used performance recordings from 30 high school brass bands who participated in Japanese regional brass band competitions (final qualifying rounds) as the experimental stimuli. In the competitions, the scoring and ranking of each band were conducted by a panel of judges who independently evaluated various musical elements such as tone quality, technique, and expression. Based on these evaluations, the bands were awarded gold, silver, or bronze distinctions. The bands with the highest scores then advanced to national-level competitions.

To minimize disparities in performance quality, we selected only bands that had received gold awards, representing approximately the top 30% of participants. This selection was based on findings by Mehr et al. [30], who reported that the sight-over-sound effect was not observed when stimuli exhibited marked differences in performance skill.

The musical pieces used as stimuli were selected from the compulsory pieces of brass band competitions held between 2012 and 2018. Each year, the brass band association selects five assigned pieces, and participating bands must choose one piece to perform. Each stimulus set therefore consisted of three different bands performing the same piece. We compiled 10 distinct sets, each containing three different bands performing the same piece (for further details on the composition of all sets, the participating bands, and the pieces performed, see S1 Text and S1 Fig in S1 File). In each set, one band qualified for the All Japan Brass Band Competition, whereas the other two bands ranked highly in the regional competitions but did not advance to the national level.

Three different audio and visual conditions (AO, VO, and AV) were prepared for each set of stimuli. For AO stimuli, only auditory information was extracted from the recordings, and the visual information was changed to a black image. For VO stimuli, audio information was separated from the recorded video and silent videos were created. For AV stimuli, we did not edit the AV information. The length of each experimental stimulus was set to 6 s, as in previous studies. This stimulus length is generally chosen to explore whether the phenomenon known as “thin-slices,” in which visual information has a marked and immediate impact on human social cognition [51], also applies to the evaluation of musical performance (which is believed to rely heavily on auditory information). All three stimuli in each group were excerpts from the same part of the piece. We extracted videos that showed as many performers as possible, including the conductor, and where the camera angles differed as little as possible between the videos in the same group.

### Procedure

The experiment was conducted using the online survey platform Qualtrics (https://www.qualtrics.com/). Randomization of the presentation order of sets and stimuli was conducted using the randomization methods with the Evenly Present Elements option in Qualtrics.

The online experiment consisted of two parts: 1) a questionnaire on the participants’ backgrounds, and 2) the judgment experiment. The questionnaire included questions for the participants about their history playing in brass bands and their experiences of playing in brass band groups and participating in brass band competitions. The last question in the questionnaire investigated whether they believed that auditory or visual information was more important for judging brass band competitions (for the results of the last question, see S2 Text and S2 Fig in S1 File).

After completing the questionnaire, participants were automatically redirected to the website for the second part of the experiment. Each participant was randomly assigned to one of the following three conditions: AO, VO, or AV. The participants were asked to perform the tasks alone in a quiet room. Moreover, participants in the two conditions with auditory information were asked to use headphones or earphones if possible, and were instructed to adjust the volume of the audio system to a comfortable level before starting the test using three sample videos.

**Fig 1:**
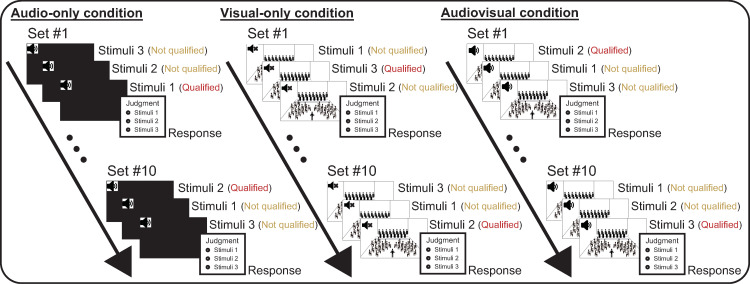
Overview of the judgment experiment. Each participant performed a three-choice performance judgment task 10 times. The order of questions and playback sequence of the three stimuli in each set were both randomized. Participants were asked to listen to and/or watch three consecutive performance recordings from a single set and then choose the one they believed would achieve the highest rating in the competition. The experiment was conducted with a between-subject design; each participant participated in only one of the three experimental conditions. Note that the stimulus images in the procedures for the visual-only and audio-visual conditions are not actual experimental stimuli; they are reference images created by the authors.

In the judgment experiment, participants were asked to select the performance that they felt would achieve the best results in a competition from three stimuli in each set (Fig 1). Participants completed 10 judgment tasks for the brass band performance-assigned experimental condition. The 10 stimulus sets were presented in a random order. Within each set, the performances of the three bands were also randomly ordered. Each video was displayed individually on a screen. When the participant selected the “Next” button (located at the bottom right of the screen), the page was switched, and the next stimulus was played. Similar to previous studies [1,30,32,33], participants were instructed to play each stimulus only once, and they were not allowed to return to the previous page. After the third band performance was played, the page switched to the response page, where participants selected the stimulus that they considered would receive the highest evaluation in the competition.

### Statistical analysis

Participant responses were evaluated using the actual results of the brass band competition, which were treated as the correct answers. The responses were then scored as either correct or incorrect. Next, the percentage of correct responses for each participant was calculated (e.g., if a participant correctly identified the winners in 8 out of 10 sets of stimuli, the percentage of correct responses was calculated as 80%).

To assess the normality of the data, the Shapiro–Wilk test was performed and both kurtosis and skewness were calculated. The distribution of all participants (ALLs), BMs, and NBMs departed significantly from normality, whereas a normal distribution was identified for NMs (for results of the Shapiro–Wilk test, kurtosis, and skewness, see S1 Table in S1 File). Nonparametric tests were therefore used for the data analysis of all groups. To compare the percentage of correct responses between conditions, we used the Kruskal–Wallis test (*α* = 0.05), which can be used even when sample sizes in the compared groups are not equal. In addition, we used the Steel–Dwass test for multiple comparisons as a post hoc test (adjusted *α* = 0.05). To analyze the difference between chance (33.33%) and the mean accuracy rate, we used the one-sample Wilcoxon test (adjusted *α* = 0.016). We also conducted supplementary analyses to compare accuracy rates between BMs with and without musical experience outside of brass bands, to compare accuracy rates within each condition, and to examine the effect of sex on accuracy rates. The effect of musical experience outside of brass bands on the accuracy rates of BMs was tested using two-way analysis of variance (ANOVA) with musical experience (presence or absence of musical experience outside of brass bands) and experimental conditions (*α* = 0.05). For comparisons within conditions, the Kruskal–Wallis test (adjusted *α* = 0.05) and the Steel–Dwass test for multiple comparisons (adjusted *α* = 0.05) were used, similar to the comparisons across conditions. The effect of sex on accuracy rates was tested using two-way ANOVA with sex and experimental conditions (*α* = 0.05), and using ANCOVA with accuracy rate as the dependent variable, experimental condition as the independent variable, and sex as a covariate (*α* = 0.05). Data analyses were performed using R and MATLAB 2021b software. All data and codes used in this study are available from the Open Science Framework (OSF; https://osf.io/7e6gx/).

## Results

### Demographics and musical training background

A total of 301 adults participated in the study (70 men and 231 women, mean age = 34.81 years, standard deviation [SD] = 11.71 years, range = 18−56 years). The number of participants in each group and in each experimental condition was as follows: BMs: 171 (46 men and 125 women; AO: 52, VO: 58, AV: 61); NBMs: 78 (16 men, 62 women; AO: 32, VO: 21, AV: 25); and NMs: 52 (8 men and 44 women; AO: 21, VO: 18, AV: 13) (Tables 1 and 2).

**Table 1:** Demographics and musical training background.

	Age (years)	Sex (female/male)	Duration of musical training (years)	Age of commencement of musical training (years)	Duration of brass-band training (years)	Age of commencement of brass-band training (years)
Brass band musicians (*n* = 171)	33.08 (11.57, 18–56)	125/46	10.98 (8.79, 1–48)	7.06 (4.88, 2–34)	8.07 (5.37, 1–35)	12.25 (1.88, 7–19)
Non-brass band musicians (*n* =78)	35.39 (13.03, 18–58)	62/16	11.72 (7.81, 1–34)	7.35 (5.10, 2–28)		
Non-musicians (*n* = 52)	39.15 (8.73, 19–55)	44/8				

Data are shown as the mean (SD, range) or number.

**Table 2:** Number of participants in each experimental condition.

	Audio-only condition	Visual-only condition	Audio-visual condition
Brass band musicians (*n* = 171)	52	58	61
Non-brass band musicians (*n* = 78)	32	21	25
Non-musicians (*n* = 52)	21	18	13
All participants (*N* = 301)	105	97	99

All BMs had musical training experience in brass bands (mean = 8.07 years, SD = 5.37 years, range = 1−35 years; Table 1). Furthermore, 137 of the 171 BMs had participated in brass band competitions, and 12 had participated at the highest competition level (e.g., All Japan Brass Band Competition). Eighteen BMs reported experience as brass band clinicians.

Among the BMs, 118 participants had musical training other than brass band training (mean = 10.98 years, SD = 8.79, range = 1−48 years; vocal: 6, keyboards: 96, strings: 3, wind instruments: 26, percussion: 2). The mean duration of musical training in NBMs was 11.72 years (SD = 7.81, range = 1−34 years; vocal: 15, keyboards: 39, strings: 14, wind instruments: 3, percussion: 3, traditional Japanese instruments: 4) (Table 1).

There were no significant differences in musical training duration (*W* = 5311, *p* = 0.63, *r* = 0.03) or the age at which music training commenced (BMs: mean = 7.51 years, SD = 49.2, range = 2−34 years; NBMs: mean = 7.35 years, SD = 5.10, range 2−28 years; W = 4664.5, *p* = 0.26, *r* = 0.08) between the BMs and NBMs. Eleven BMs and 10 NBMs had participated in musical competitions in genres other than brass band music.

### Judgment experiment

#### ALLs.

**Fig 2:**
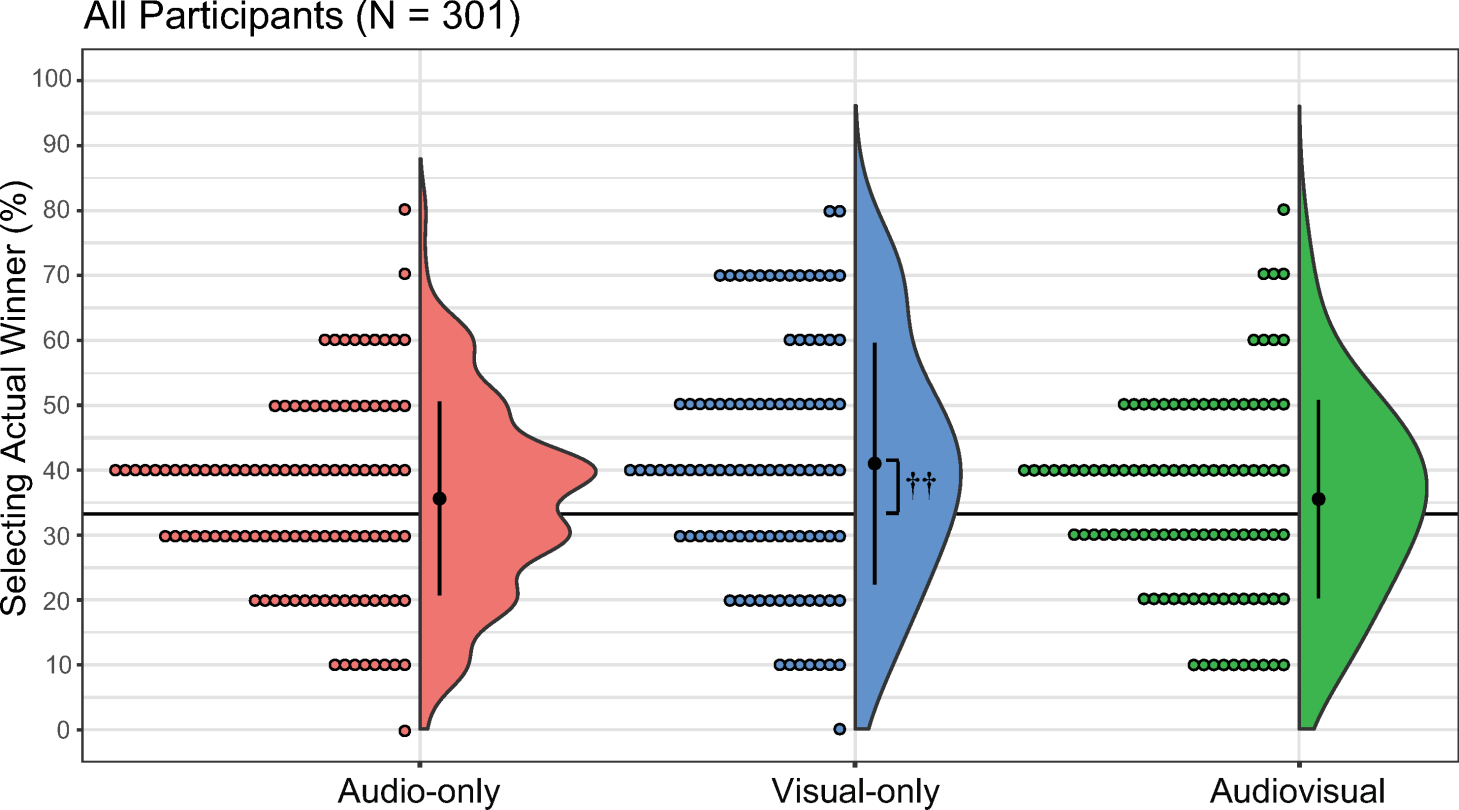
Percentage of actual winner selection for each condition in all participants. The audio-only, visual-only, and audio-visual conditions are denoted as red, blue, and green, respectively. Error bars denote the standard error of the percentage of selecting the actual winner. Each dot corresponds to data from an individual participant. ^††^*p* < 0.01 (accuracy rate vs. chance).

For ALLs, the mean percentage of correct responses was 35.62% in the AO condition (median = 40, SD = 14.93, 95% confidence interval [CI]: [32.98, 38.81]), 40.92% in the VO condition (median = 40, SD = 18.56, 95% CI: [36.94, 44.78]), and 35.50% in the AV condition (median = 40, SD = 15.20, 95% CI: [32.30, 38.78]; Fig 2). The Kruskal–Wallis test revealed no significant difference between the conditions (*H*(2) = 5.42, *p* = 0.07, *η*^2^ = 0.02). The one-sample Wilcoxon signed-rank test indicated no significant differences between the mean percentage of correct responses and chance in both the AO and AV conditions (AO: *V* = 3405, *p* = 0.05, *r* = 0.40 and AV: *V* = 2977, *p* = 0.08, *r* = 0.18, respectively). However, a significant difference was observed between the accuracy rate and chance in the VO condition (*V* = 3468, *p* < 0.01, *r* = 0.40).

#### BMs.

**Fig 3:**
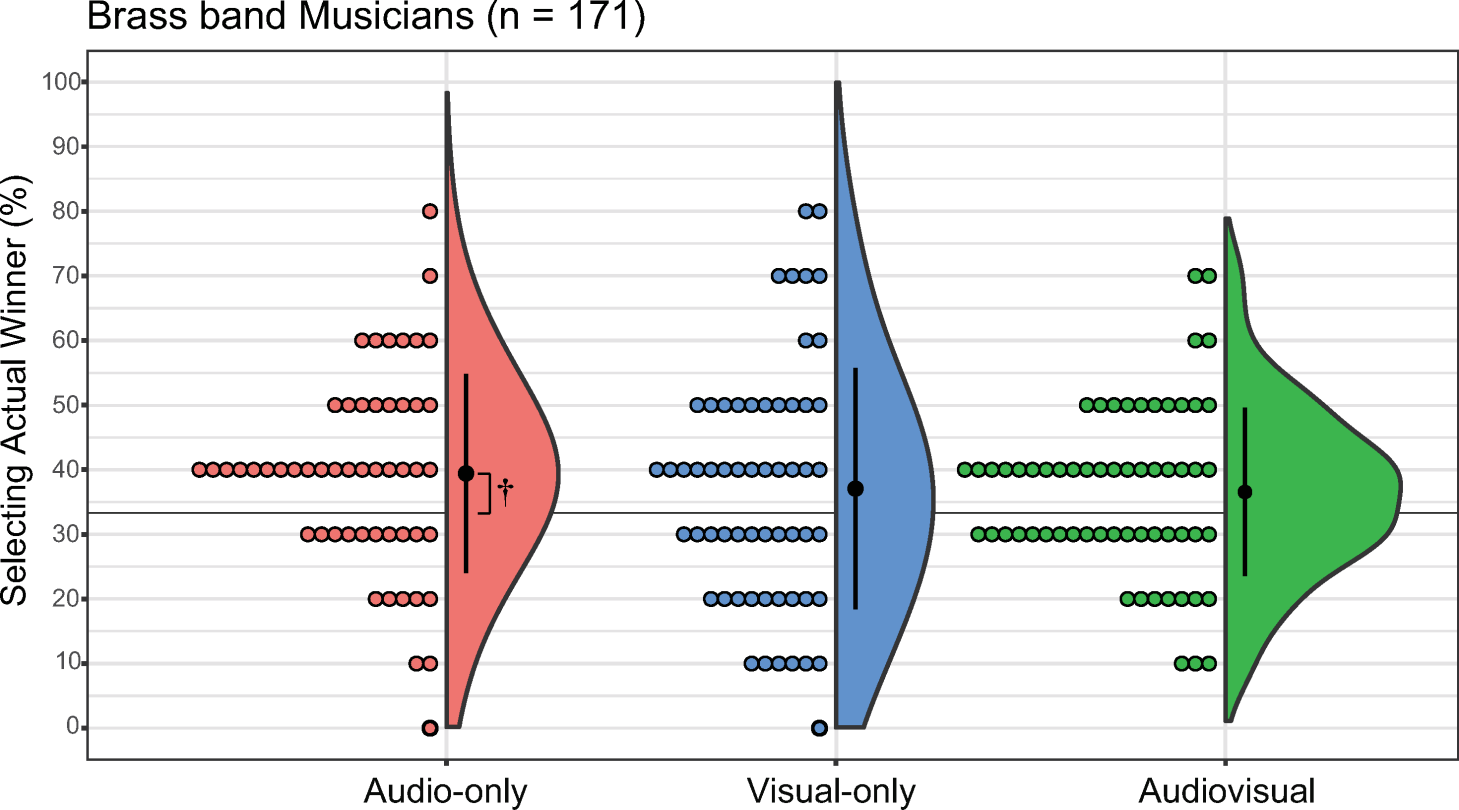
Percentage of actual winner selection for each condition in brass-band musicians. The audio-only, visual-only, and audio-visual conditions are denoted as red, blue, and green, respectively. Error bars denote the standard error of the percentage of selecting the actual winner. Each dot corresponds to data from an individual participant. ^†^*p* < 0.01 (accuracy rate vs. chance).

For the BM group, the mean percentage of correct responses was 39.42% in the AO condition (median = 40, SD = 15.39, 95% CI: [35.14, 43.71]), 37.07% in the VO condition (median = 40, SD = 18.64, 95% CI: [32.17, 41.97]), and 36.56% in the AV condition (median = 40, SD = 13.02, 95% CI: [33.22, 39.89]; Fig 3). The Kruskal–Wallis test revealed no significant difference between the conditions (*H*(2) = 1.47, *p* = 0.48, *η*^*2*^ = 0.03). The one-sample Wilcoxon signed-rank test indicated no significant differences between the mean percentage of correct responses and chance in both the VO and AV conditions (VO: *V* = 1053, *p* = 0.13, *r* = 0.20 and AV: *V* = 1265, *p* = 0.02, *r* = 0.30, respectively). However, a significant difference was observed between the accuracy rate and chance in the AO condition (*V* = 1033, *p* < 0.01, *r* = 0.44).

#### NBMs.

**Fig 4:**
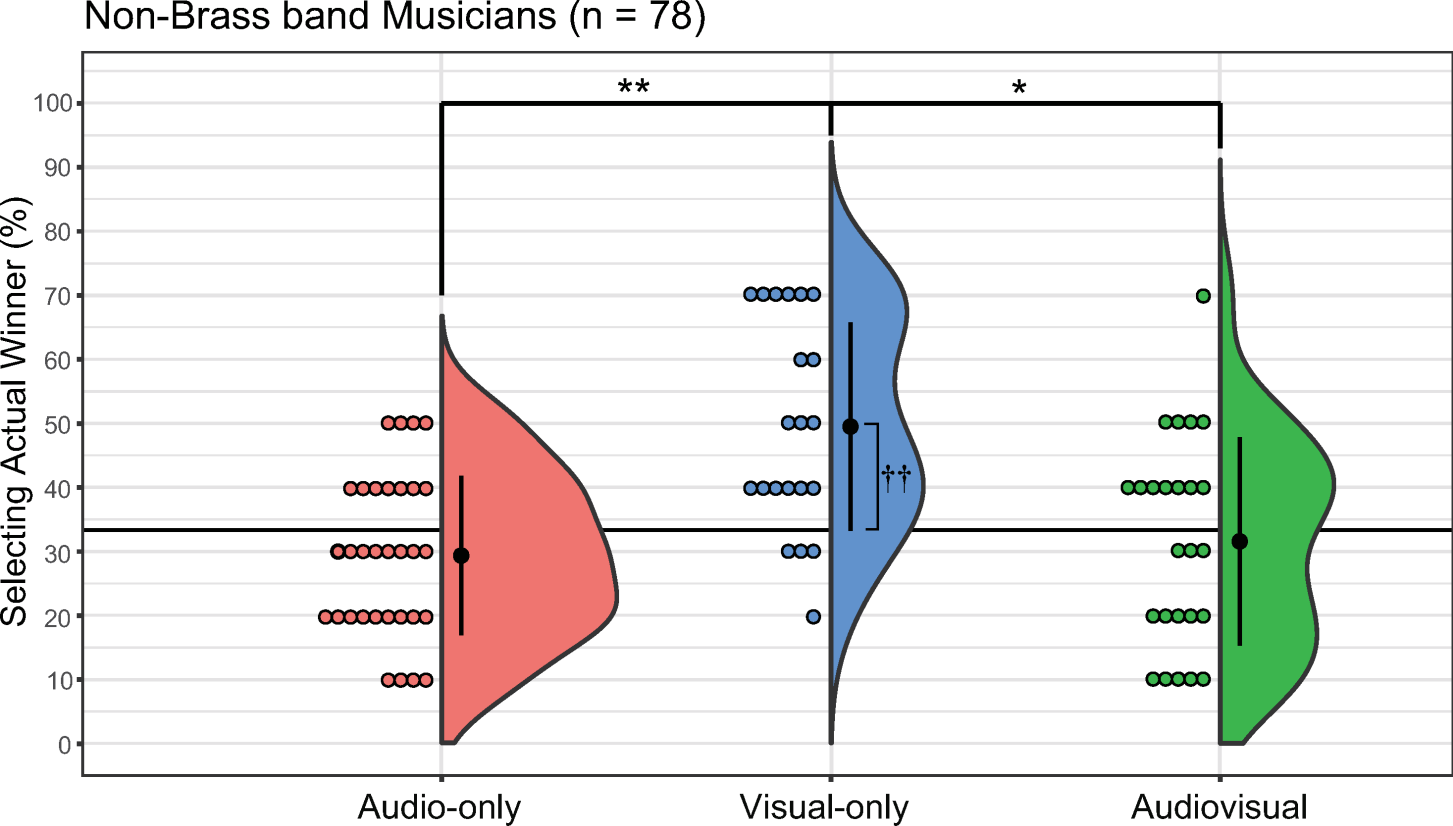
Percentage of actual winner selection for each condition in non-brass band musicians. The audio-only, visual-only, and audio-visual conditions are denoted as red, blue, and green, respectively. Error bars denote the standard error of the percentage of selecting the actual winner. Each dot corresponds to data from an individual participant. *p = 0.05, **p < 0.01 (between conditions); ^††^*p* < 0.01 (accuracy rate vs. chance).

For the NBM group, the percentage of correct responses was 29.37% in the AO condition (median = 30, SD = 12.43, 95% CI: [24.89, 33.86]), 49.52% in the VO condition (median = 50, SD = 16.27, 95% CI: [42.12, 56.93]), and 31.60% in the AV condition (median = 30, SD = 16.25, 95% CI: [24.89, 38.31], Fig 4). The Kruskal–Wallis test revealed significant differences between conditions (*H*(2) = 16.93, *p* < 0.01, *η*^2^ = 0.53, 95% CI for effect size: [0.06, 0.39]). The Steel–Dwass test indicated significant differences between the AO and VO conditions (*t* = 4.01, *p* < 0.01, *r* = 0.63) and between the VO and AV conditions (*t* = 3.13, *p* < 0.01, *r* = 0.50). The percentage of correct responses in the VO condition was significantly higher than that in the AO or AV condition. The one-sample Wilcoxon signed-rank test revealed no significant differences between the mean percentage of correct responses and chance in both the AO and AV conditions (AO: *V* = 190, *p* = 0.17, *r* = 0.24 and AV: *V* = 144, *p* = 0.63, *r* = 0.10, respectively). However, a significant difference was observed between the accuracy rate and chance in the VO condition (*V* = 215, *p* < 0.01, *r* = 0.76).

#### NMs.

**Fig 5:**
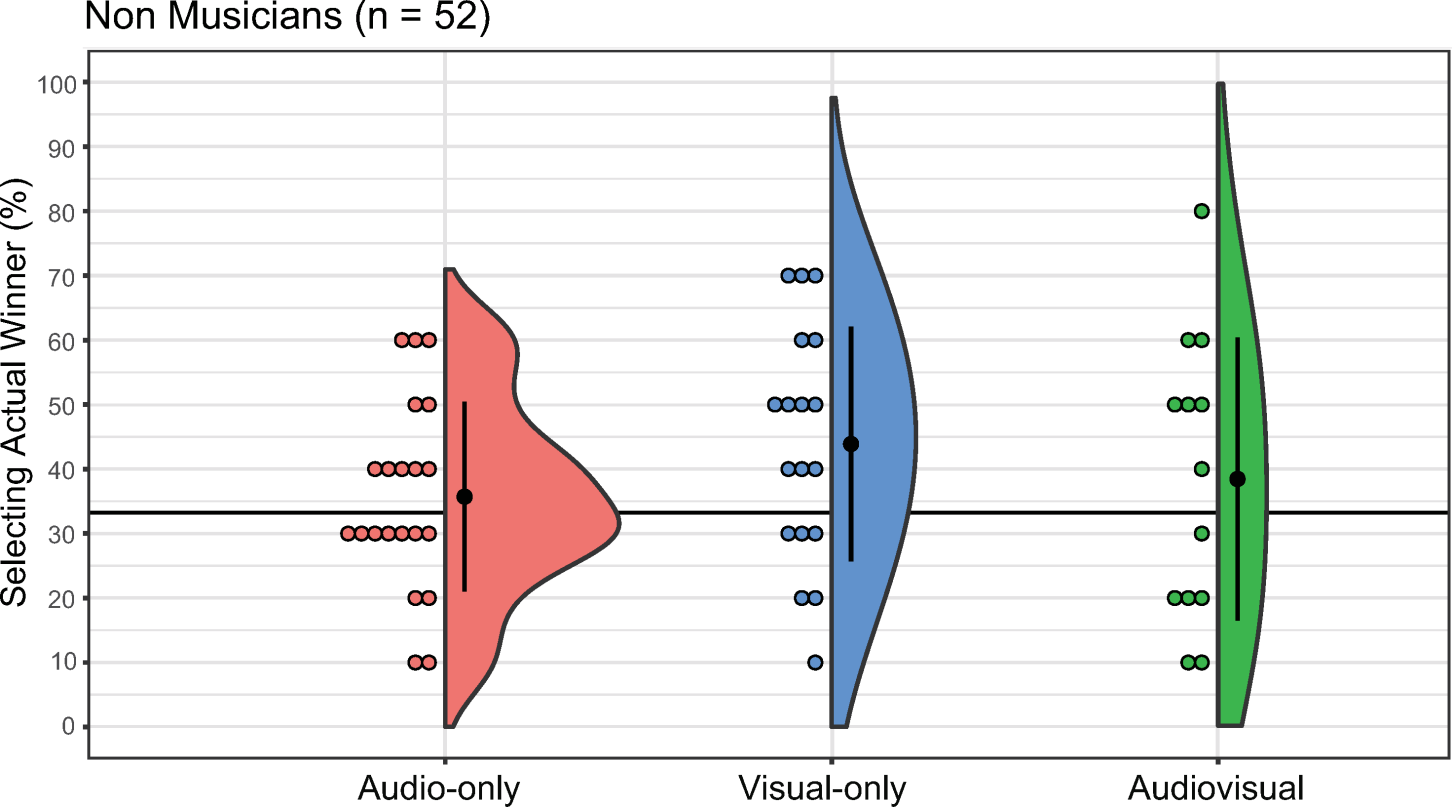
Percentage of actual winner selection for each condition in non-musicians. The audio-only, visual-only, and audio-visual conditions are denoted as red, blue, and green, respectively. Error bars denote the standard error of the percentage of selecting the actual winner. Each dot corresponds to data from an individual participant.

For the NM group, the percentage of correct responses was 35.71% in the AO condition (median = 30, SD = 14.69, 95% CI: [29.03, 42.40]), 43.16% in the VO condition (median = 40, SD = 18.19, 95% CI: [34.84, 52.94]), and 37.86% in the AV condition (median = 45, SD = 21.93, 95% CI: [25.21, 51.71]; Fig 5). The Kruskal−Wallis test revealed no significant difference between the conditions (*H*(2) = 1.97, *p* = 0.37, *η*^*2*^ = 0.04). The one-sample Wilcoxon signed-rank test indicated that none of the percentages exceeded chance (AO: *V* = 141, *p* = 0.38, *r* = 0.19; VO: *V* = 137, *p* = 0.03, *r* = 0.53; AV: *V* = 59, *p* = 0.36, *r* = 0.25).

### Effects of musical experience outside brass bands in BMs

There was no main effect of musical experience (*F*(1, 165) < 0.01, *p* = 0.98, ω^2^ < 0.01) and no interaction between the presence or absence of musical experience outside of brass bands and the experimental conditions (*F*(2, 165) = 1.42, *p* = 0.24, ω^2^ < 0.01). It is therefore unlikely that musical experience outside of brass bands influenced the results of the current experiment.

### Group comparisons by conditions

#### AO condition.

**Fig 6:**
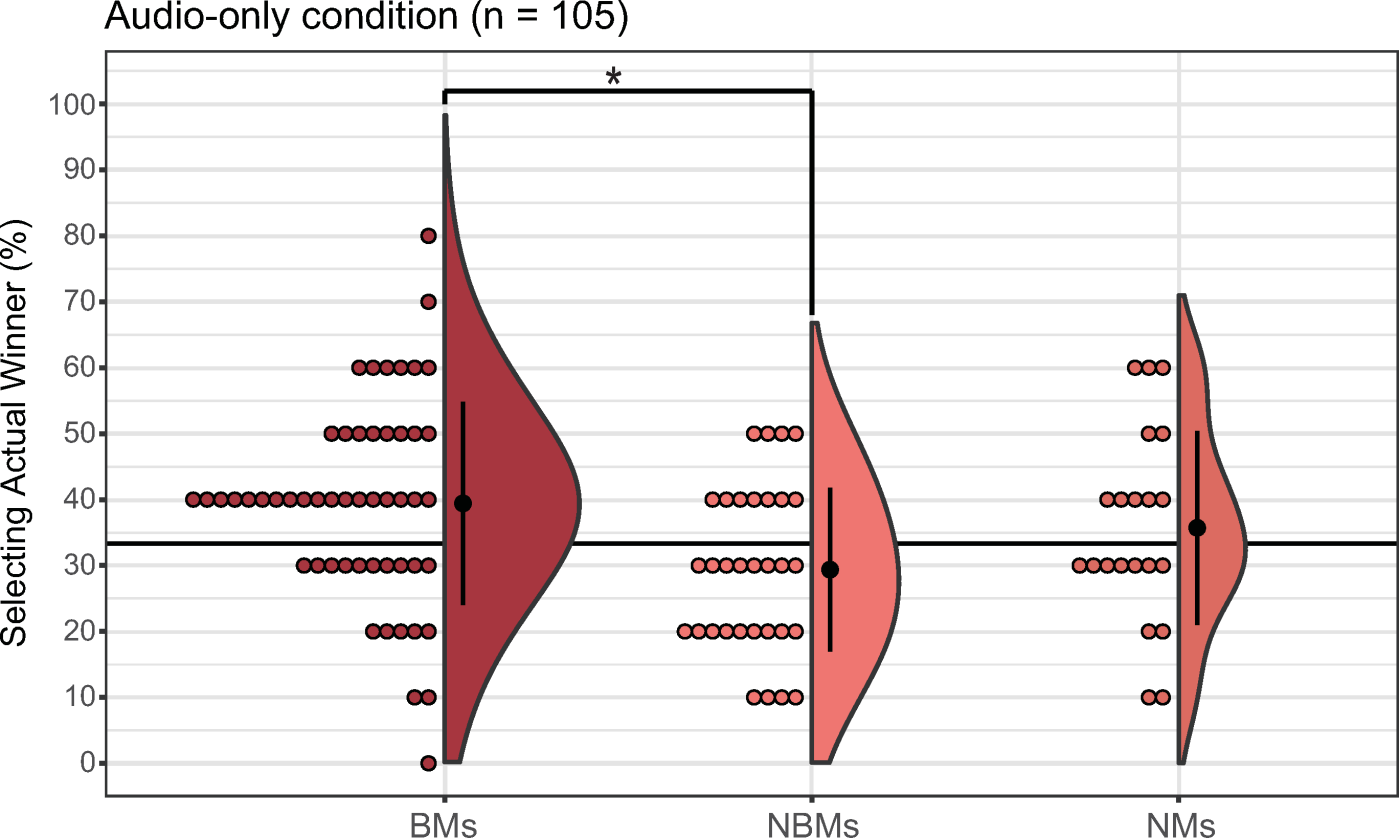
Group comparison in the audio-only condition. The left, middle, and right plots represent the results for BMs, NBMs, and NMs, respectively. Error bars denote the standard error of the percentage of selecting the actual winner. Each dot corresponds to data from an individual participant. *p < 0.01 (between groups).

The percentage of correct responses was 39.42% (median = 40, SD = 15.39, 95% CI: [35.14, 43.71]) for BMs, 29.37% (median = 30, SD = 12.43, 95% CI: [24.89, 33.86]) for NBMs, and 35.71% (median = 30, SD = 14.69, 95% CI: [29.03, 42.40]) for NMs. The Kruskal–Wallis test revealed significant differences in the percentage of correct responses (*H*(2) = 9.32, *p* < 0.01, *η*^2^ = 0.07, 95% CI for effect size: [0.0018, 0.22]; Fig 6), and the Steel–Dwass test indicated a significant difference between BMs and NBMs (*t* = 3.03, *p* < 0.01, *r* = 0.35).

#### VO condition.

**Fig 7:**
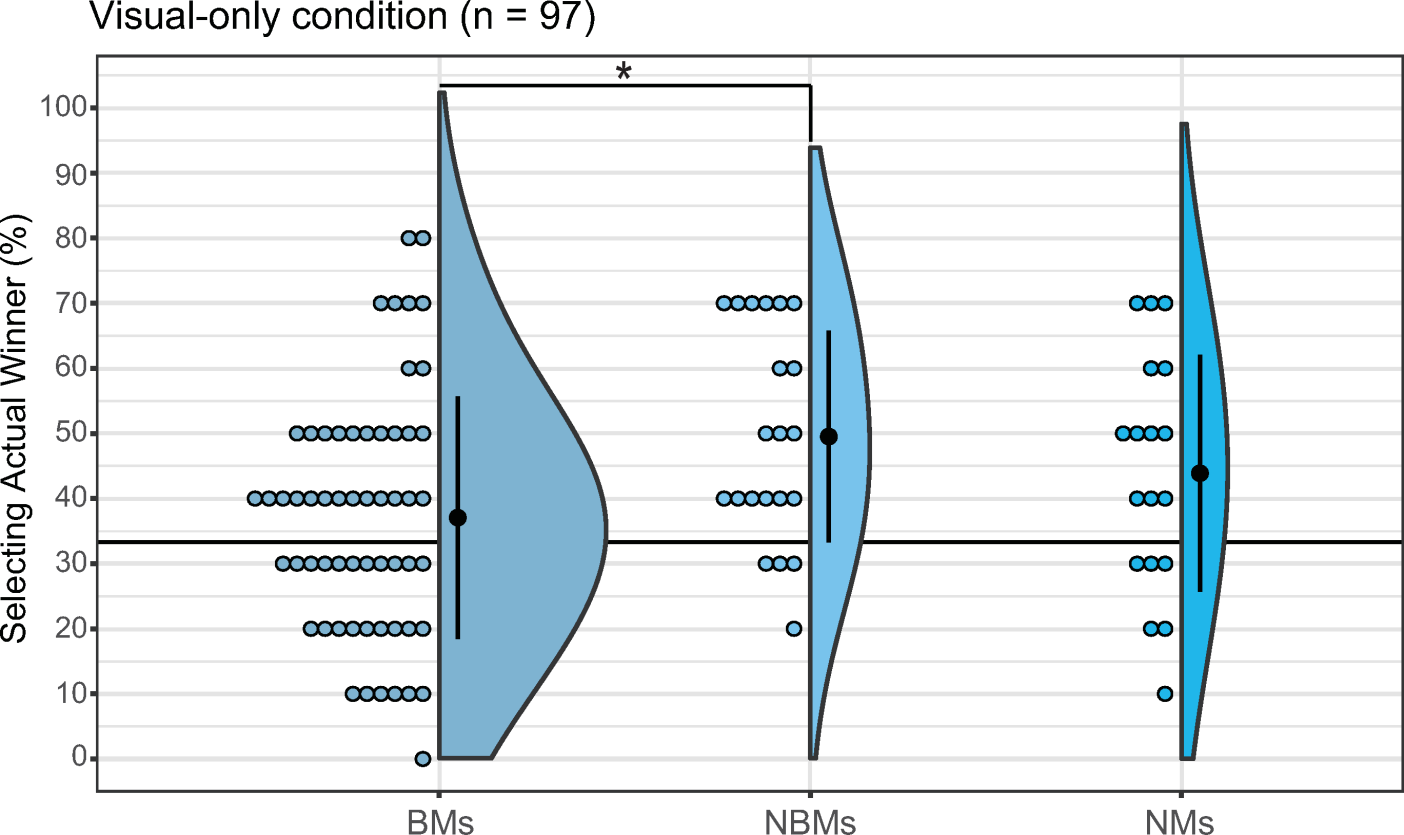
Group comparison in the visual-only condition. The left, middle, and right plots represent the results for BMs, NBMs, and NMs, respectively. Error bars denote the standard error of the percentage of selecting the actual winner. Each dot corresponds to data from an individual participant. *p = 0.03 (between groups).

The percentage of correct responses was 37.07% (median = 40, SD = 18.64, 95% CI: [32.17, 41.97]) for BMs, 49.52% (median = 50, SD = 16.27, 95% CI: [42.12, 56.93]) for NBMs, and 43.16% (median = 40, SD = 18.19, 95% CI: [34.84, 52.94]) for NMs. The Kruskal–Wallis test revealed significant differences in the percentage of correct responses (*H*(2) = 7.32, *p* = 0.03, *η*^2^ = 0.06, 95% CI for effect size: [0, 0.21]; Fig 7), and the Steel–Dwass test indicated a significant difference between BMs and NBMs (*t* = 2.58, *p* = 0.03, *r* = 0.41). However, because the lower limit of the 95% CI for the effect size was 0, this result was not considered to have a significant effect.

#### AV condition.

**Fig 8:**
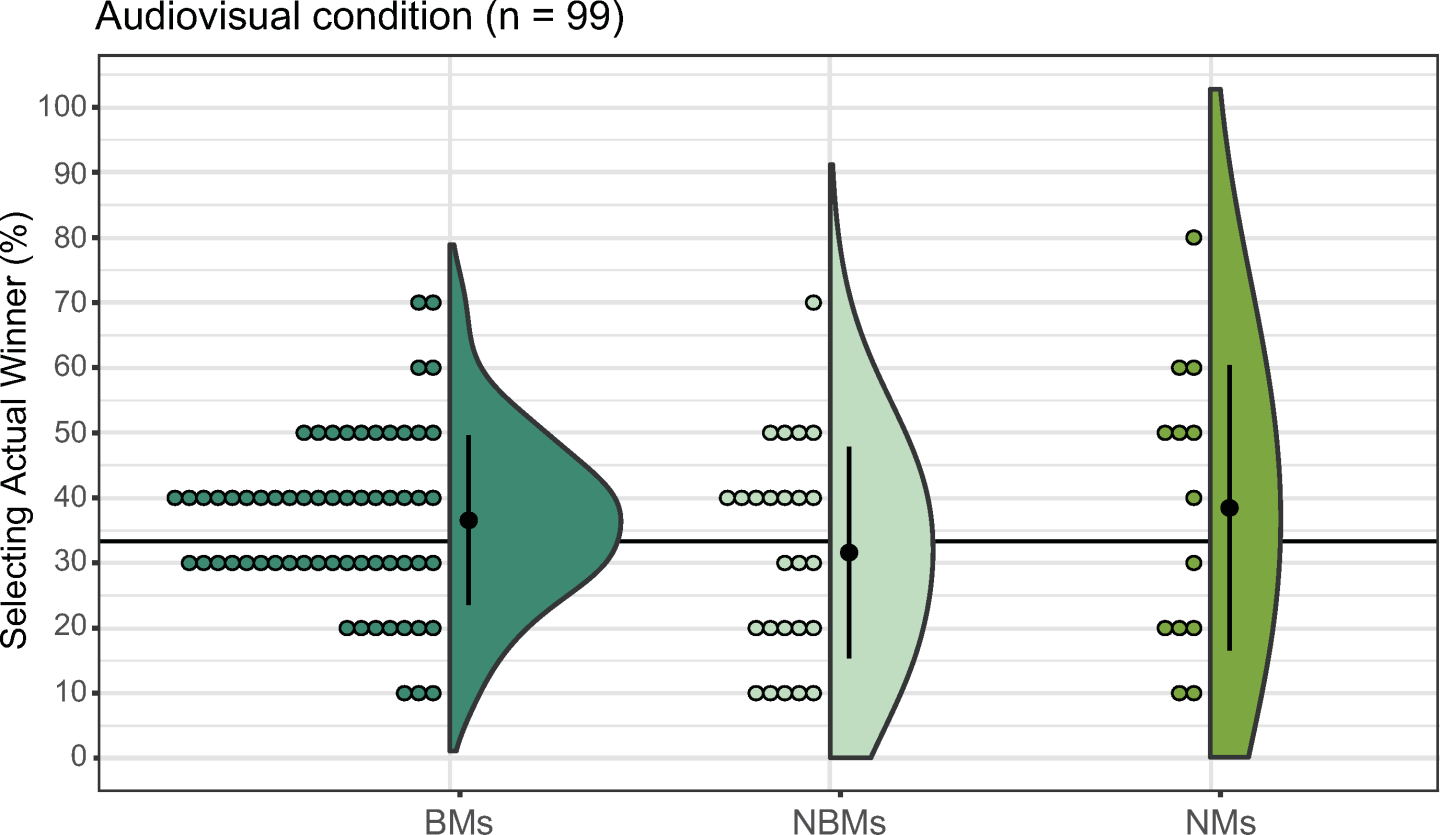
Group comparison in the audio-visual condition. The left, middle, and right plots represent the results for BMs, NBMs, and NMs, respectively. Error bars denote the standard error of the percentage of selecting the actual winner. Each dot corresponds to data from an individual participant.

The percentage of correct responses was 36.56% (median = 40, SD = 13.02, 95% CI: [33.22, 39.89]) for BMs, 31.60% (median = 30, SD = 16.25, 95% CI: [24.89, 38.31]) for NBMs, and 37.86% (median = 45, SD = 21.93, 95% CI: [25.21, 51.71]) for NMs. A Kruskal–Wallis test revealed no significant differences in the percentage of correct responses (*H*(2) = 1.92, *p* = 0.38, *η*^2^ = 0.02; Fig 8).

### Effects of participant sex on accuracy rates

Two-way ANOVA with sex and experimental conditions revealed no significant main effect of sex (*F*(1, 295) = 0.94, *p* = 0.33, *ω*^2^ < 0.01) and no significant interaction between sex and experimental condition (*F*(2, 295) = 0.63, *p* = 0.53, *ω*^2^ < 0.01). These findings suggest that the sex of the evaluators did not significantly influence the present results.

To examine the effects of sex on accuracy rates within each participant group, ANCOVA was conducted with accuracy rate as the dependent variable, experimental condition as the independent variable, and sex as a covariate. For the ALLs, the condition had a significant effect on accuracy rates after controlling for participants’ sex (*F*(2, 1948) = 3.65, *p* = 0.02, *p*η**² = 0.02). By contrast, in the BM group, no significant effect of condition on accuracy rates was observed after controlling for sex (*F*(2, 255) = 0.51, *p* = 0.60, *p*η**² = 0.006). In the NBM group, condition significantly affected accuracy rates even after controlling for sex (*F*(2, 5711) = 13.10, *p* < 0.01, *p*η**² = 0.26). However, in the NM group, no significant effect of condition on accuracy rates was observed after accounting for sex (*F*(2, 658) = 1.01, *p* = 0.37, *p*η**² = 0.04). Thus, the ANCOVA results, which included sex as a covariate, were consistent with those obtained without including sex. These findings further suggest the minimal influence of participants’ sex on the study findings.

## Discussion

The aim of the present study was to investigate the replicability of the sight-over-sound effect when judging Japanese brass band competitions under controlled conditions. Our primary hypothesis was that the sight-over-sound effect would not be replicable in the judgment of Japanese brass band competitions when the stimuli were more controlled relative to previous studies. Our secondary hypothesis proposed that BMs would not exhibit the sight-over-sound effect because of their abilities gained from brass band experiences; that is, we expected them to select the actual winners with higher accuracy in AO and AV conditions than in VO conditions. Our analysis of all participants revealed no significant differences between the AO, VO, and AV conditions, demonstrating that our overall sample did not demonstrate the sight-over-sound effect (Fig 2). However, our subgroup analyses of BMs, NBMs, and NMs revealed that the sight-over-sound effect was present in the NBMs but absent in the BMs and NMs (Figs 3-5). Furthermore, the effect size observed in the NBM results (*η*^2^ = 0.53) was relatively large, indicating a meaningful difference [52] among the three conditions. Collectively, our findings indicate that the replicability of the sight-over-sound effect in the judgment of Japanese brass band competitions is limited and depends on the musical experience of the evaluator.

How should our results be interpreted in relation to previous studies on the sight-over-sound effect? Both the initial study by Tsay [1] and its replication by Mehr et al. [30] demonstrated a higher accuracy in the VO condition than in the AO and AV conditions. Tsay [33] also observed a similar trend in orchestra performances. By contrast, the sight-over-sound effect was only replicated in the NBMs in our study, but not in the ALLs, BMs, or NMs. We suggest at least four factors that may explain the similarities and differences between our investigation and prior studies: (1) stimulus control, (2) variation in performance levels and genres, (3) differences between solo and ensemble performances, and (4) musical experience of evaluators.

### Stimulus control

In our study, we used recordings with controlled camera angles for all performances, thus addressing the issue identified by Mehr et al. [30] who proposed that varying camera angles might influence the sight-over-sound effect. Our findings indeed indicate that controlled camera angles, which reduce visual disparities, may diminish the sight-over-sound effect. In this way, our study contrasts with previous studies in which varied camera angles were used [1,29,30,33]. Additionally, unlike studies that used stimuli comprising different musical pieces, our study used the same pieces performed by different brass band groups. This control of musical pieces likely led to more consistent player movements, thereby potentially reducing the sight-over-sound effect. Conversely, the use of varied musical pieces in previous studies might have resulted in more diverse player movements because of differences in melodies, harmonies, and rhythms, which may have enhanced the ability of participants to discern between winning and losing performances. Our findings therefore suggest that both camera angles and musical piece selection are key factors for explaining the sight-over-sound effect.

### Variation in performance levels and genres

Mehr et al. [30] highlighted that the degree of variation in performance level is a significant factor influencing the sight-over-sound effect. When this variation is small, such as when the top three performers in international-level competitions are evaluated, visual dominance can be observed [1,33]. Conversely, when this variation is large, such as when competition winners are compared with competitors eliminated in earlier rounds, auditory dominance is observed [30]. Notably, however, Chiba et al. [31] recently proposed that the effects of variation in performance level may be genre-dependent; when variation was small, visual dominance was observed in Western-classical solo music competitions, whereas auditory dominance was noted in Japanese-Shamisen solo music competitions [31]. These authors suggested that such differences in the dominance of auditory and visual cues may occur as the result of movements induced by music genres from different cultures.

In the present study, the variation in performance level was considered small because all of the bands were winners in the preliminary rounds, and there were likely only minor differences in performance during the final qualifying rounds. The degree of variation in performance level in our study may therefore be perceived as small, akin to the conditions in previous studies that examined the top three performers in international-level competitions [1,30,33]. However, despite these similar conditions, we did not observe the sight-over-sound effect in our overall sample (Fig 2). This discrepancy might be attributed to the differences in performance genres from different cultures. Similar to the conclusions of Chiba et al. [31], our findings suggest that the effects of variations in performance level may be both genre- and culture-dependent. Taken together, the interplay between variation in performance level and genre might be a crucial factor for interpreting the sight-over-sound effect. Further research into the sight-over-sound effect across diverse musical contexts, as suggested by Chiba et al. [31], will help to identify how variations in performance level and genre influence performance evaluations.

### Differences between solo and group performances

It is crucial to consider the distinctions between solo and group performances. In previous studies, piano, violin [1,30,33], and Tsugaru-shamisen [31] competitions featured single-player performances, whereas in the present study, we evaluated performances by multiple players in a brass band. We propose that, for evaluators, the focus of attention may differ between solo and ensemble performances. It is expected that in the evaluation of a solo performance, attention is primarily directed toward a single performer, whereas in the assessment of a performance involving multiple players, attention may transit from one performer to another, although attention is generally directed to especially relevant objects or targets [53]. In brass band performances, more than 10 different musical instruments are typically played, meaning that evaluators are likely to shift their focus of attention during the assessment period. Notably, differences in attentional focus between solo and group performances may influence the sight-over-sound effect.

To date, the series of research on the sight-over-sound effect (including the present study) has been conducted using recordings of actual competition performances. It is important to consider that these recordings are dependent on the filming environment, which may result in differences in the relative sizes of movements of the performers in solo versus group performances. Specifically, the movements of group performers may appear smaller than those of solo performers because of the degree of camera zoom needed to capture solo versus group performances. In solo performances, relative size is generally larger, thereby allowing for the observation of subtle visual changes such as facial expressions. Conversely, in group performances, it is usually possible to discern the movements of the entire body and the relationships of movements between performers rather than the detailed movements of each individual. Future research should therefore consider using videos with a standardized distance between the performers and evaluators, or should attempt to use experimental environments that are similar to those of actual concert halls, to further investigate the sight-over-sound effect.

Another aspect that is worth considering pertains to performer interactions during ensemble performances. The degree of body movement synchronization among ensemble performers is reportedly correlated with the evaluation of performance quality and proficiency with the musical piece [54–56]. In addition, both auditory information (e.g., the intensity of the performed sound) and visual information (e.g., the coordination of body movements) significantly contribute to the evaluation of togetherness among ensemble performers [57]. In the videos used as stimuli in the present study, we noted apparent differences in the degrees of performer interactions among the different brass bands. In future studies, it would be intriguing to examine how performer interactions among multiple players influences the judgment of brass band performances. Furthermore, research examining the neural activities and learning effects involved in temporal coordination during social activities such as ensemble performances has also become increasingly active [58–62]. It may therefore be of interest to investigate the effects of performer-specific behaviors, stemming from ensemble experience, on performance evaluation.

### Musical experience of evaluators

A significant finding of the present study was the variation in results depending on the musical experience of the evaluator; the sight-over-sound effect was not observed in BMs, whereas it was present in NBMs. Additionally, the presence or absence of musical experience other than brass band experience in the BM group had no effects on the results. Previous research has demonstrated that experience with specific music genres can shape neural responses to musical features [63], as well as sensorimotor synchronization and timing perception [64]. The similar trends observed among BMs with and without musical experience other than brass band experience may be attributable to the specialized exposure of evaluators to brass band performances, which may have uniquely influenced their perceptual judgments. When comparing the average accuracy rates for each experimental condition across participant groups, the accuracy rate of BMs was significantly higher than that of NBMs in the AO condition (Fig 6). Conversely, in the VO condition, the accuracy rate of NBMs was significantly higher than that of BMs (Fig 7). These results suggest that when evaluating excerpts from the brass band competition used in the current study, BMs were able to judge more accurately from AO information, whereas NBMs were able to judge more accurately from VO information. Nevertheless, we did not find a main effect of musical experience outside of brass bands, suggesting that having or lacking experience in other musical genres may have only a minor effect among BMs.

Why might BMs have been more accurate than NBMs and NMs under the AO condition? We propose that the extensive experience of BMs in brass bands might have enhanced their ability to detect nuanced differences in the sounds. Compared with non-musicians, musicians pay selective attention to auditory information [48]. Moreover, musical training enhances selective auditory attention development [49]. In addition, musicians with different musical specializations reportedly have enhancements in different elements of auditory perception [65], suggesting that the type of musical experience is important when testing auditory abilities. Thus, BMs may have better selective auditory attention abilities specifically for brass band music compared with NMs or NBMs. This enhanced auditory discernment might also stem from the development of auditory–motor connections and the mirror neuron system, which may both be fortified through rigorous brass band practice. Mirror neurons react to one’s own actions and the observation of actions of others [66,67], as well as to related sounds [68–70]. Previous studies have reported these auditory–motor interactions in musicians [71–74] as well as heightened conscious motor imagery in response to sounds [38]. Consequently, we hypothesize that BMs can anticipate the movements of a performer based on the sounds that they perceive, thereby enabling them to discern fine, nuanced sound variations. This skill is particularly pronounced in the AO condition, potentially leading to the observed higher accuracy rates in BMs.

Why were the NBMs able to identify the actual winners above the chance level solely in the VO condition? It may be that their non-brass band musical experience might allow them to discern performance quality through the visual appeal of the movements. It is reasonable to assume that the NBMs predominantly relied on visual information, influenced by their diverse non-brass band musical background; this assumption aligns with the results of previous studies that have examined the sight-over-sound effect [1,33]. Additionally, in Japanese brass band performances, the visual aspects of a performance are often crucial to a positive reception. The groups featured in our stimuli may have therefore been actively engaging the audience with their movements; such visual strategies might resonate more with NBMs, thus influencing their assessments. The visual dominance observed in the NBMs may also be caused by this group’s strong awareness of the visual elements that are assessed during the professional judgment of music, such as signs of motivation and passion [1]. For example, previous studies have revealed that musicians understand that body movements and behaviors are an important part of musical performance [27,28]. By contrast, NBMs may not have a particularly strong awareness of the specific auditory elements that are important for evaluating brass bands.

In the NMs, although no significant differences were observed, there was a tendency toward higher accuracy rates in the VO condition compared with chance (*p* = 0.03, adjusted *α* = 0.016). This result suggests that NMs, like NBMs, may exhibit a tendency toward visual information dominance. Given their minimal musical experience, NMs might evaluate performances based on the visual appearance of their own musical experiences. Some previous research suggests that NMs are able to successfully assess musical performances from visual information only [75–77]. However, our results provide only limited support for these previous findings.

## Limitations

We consider that there are at least five limitations in this study. First, there are limitations related to online surveys. For example, certain participants may have been unintentionally excluded from the study, such as older participants, participants without access to the internet, and/or participants who are not on social media. Moreover, because the study was conducted online, it was not possible to control the devices used by the participants. The display size of the visual stimuli might have varied between participants, and differences in audio quality may also have been present. Several previous studies that examined the replicability of the sight-over-sound effect were conducted online; however, to eliminate such biases, it may be necessary to conduct measurements in more controlled environments (such as laboratories).

The second limitation was the lack of a within-subject design; the study used a between-subject design to minimize learning and transfer effects. However, this approach has limitations, including potential differences in participant characteristics across conditions. A within-subject design may address these differences but can introduce other challenges, such as order effects and familiarity with specific musical pieces, which might affect responses in later trials. Although counterbalancing may mitigate these effects, residual biases such as fatigue or increased familiarity might still affect the results. Future studies may consider the use of a within-subject design with counterbalancing to validate our findings.

The third limitation is the considerable variability of the age at which musical training began among BMs and NBMs. Previous studies have reported that structural and functional plastic changes occur in the brain as a result of music training in early childhood [78,79]. Although the present study did not consider the onset age or duration of music training, an investigation of the relationship between such musical backgrounds and the sight-over-sound effect may lead to a greater understanding of the effects of musical experience.

The fourth limitation—or an open question for future study—is why the accuracy rates in the AV condition did not surpass those in the AO or VO conditions. Given that the judging in actual music competitions is conducted in a format that closely resembles the AV condition, it remains unclear why the accuracy rates in the AV condition were at chance level. One previous study demonstrated that combining both auditory and visual information allows for a more accurate evaluation of a performer’s emotional intent than using only auditory or visual information [80]. However, the stimuli used in the previous study were longer than the 6-s clips used in our study [80,81]; it is therefore possible that our shorter stimulus duration may not have been sufficient to accurately capture these effects. Future studies are thus needed to investigate why the accuracy rates in the AV condition did not surpass those in the AO or VO conditions.

The fifth limitation involves the use of recordings from Japanese brass band competitions. To maintain auditory neutrality, we limited our selection to performances by gold-award-winning bands. However, competition rankings are not publicly available, meaning that we were unable to neutralize the auditory information based on actual rankings, as in previous studies [1,30,31,33]. Mehr et al. [30] reported that auditory information dominates when differences in stimulus levels are large, whereas visual information prevails when these differences are small. In the present study, no group showed marked auditory dominance, suggesting that we successfully controlled stimulus level differences within the given limitations. Nevertheless, the use of brass band competition videos has some advantages. With over 10,000 bands participating annually in Japan, future research might explore varying performance levels and ensemble sizes to better understand the interactions between auditory and visual information in performance evaluation.

## Conclusions

We examined the replicability of the sight-over-sound effect in the judgment of Japanese brass band competitions using controlled musical pieces and camera angles. Our results, drawn from 301 adult participants, did not replicate the sight-over sound effect in our overall sample, thereby highlighting the importance of stimulus control for replicating this effect. Furthermore, when the participants were divided into BMs, NBMs, and NMs, we observed varying effects on musical performance judgments. Our results suggest that the specific musical experience of evaluators should be considered when investigating the sight-over-sound effect.

## Supporting information

S1 File Supporting information. (DOCX)
