# Supplementary material for: Sight-over-sound effect depends on interaction between evaluators’ musical experience and auditory-visual integration: An examination using Japanese brass band competition recordings
Source: PLoS One. 2025 Apr 29;20(4):e0321442. doi: 10.1371/journal.pone.0321442 (PMC12040236; doi:10.1371/journal.pone.0321442)
Supplement: S1 File — (DOCX) [file pone.0321442.s001.docx]

**Supplementary information**

**S1 text.** **Criteria for Choosing the Experiment Stimuli.**

All the experimental stimuli for this study were sourced from recordings of the final qualifying rounds of the All Japan Brass Band Competition, covering five regional rounds: 2012 Hokuriku, 2014 Hokuriku, 2016 Kyushu, 2017 Kyushu, and 2017 East-Kanto. In Japanese brass band competitions, the Band Association annually selects five assigned pieces, with each participating group having to perform one of five assigned pieces. Leveraging this unique aspect of the Japanese brass band competition, each stimulus set included in our study consisted of three performance recordings from the same regional competition, all performing the same assigned piece. Within each set, one group was a finalist from the qualifying rounds, having advanced to the All Japan Brass Band Competition (usually the top 2-3 groups in regional competition), while the other two achieved gold awards but did not proceed to the national competition (typically the top 30% in each regional competition). The results of these regional competitions and all stimulus sets used in this study are detailed in S2 Fig.

#### Fig.S1 Structure of Each Experimental Stimulus Sets


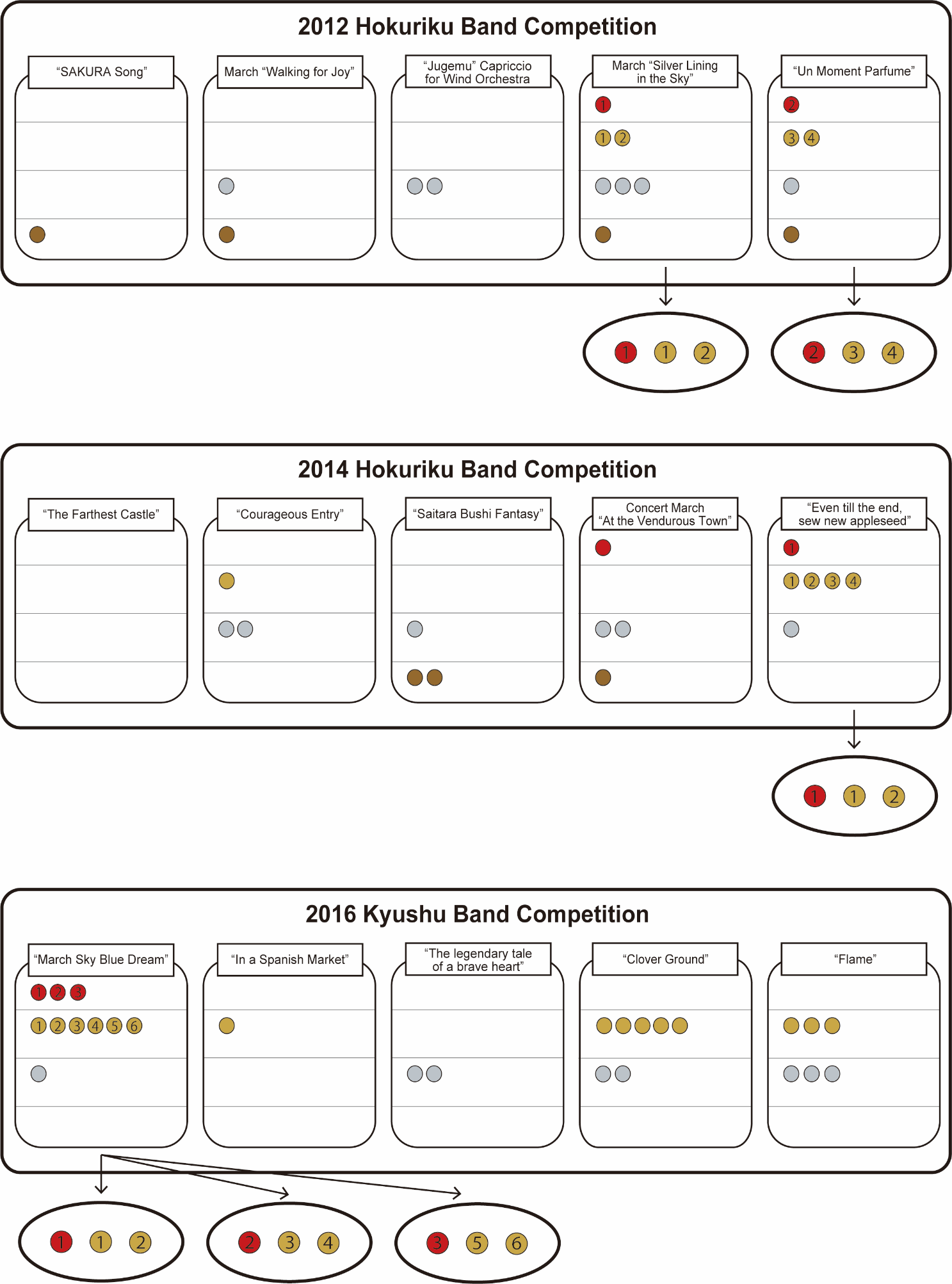


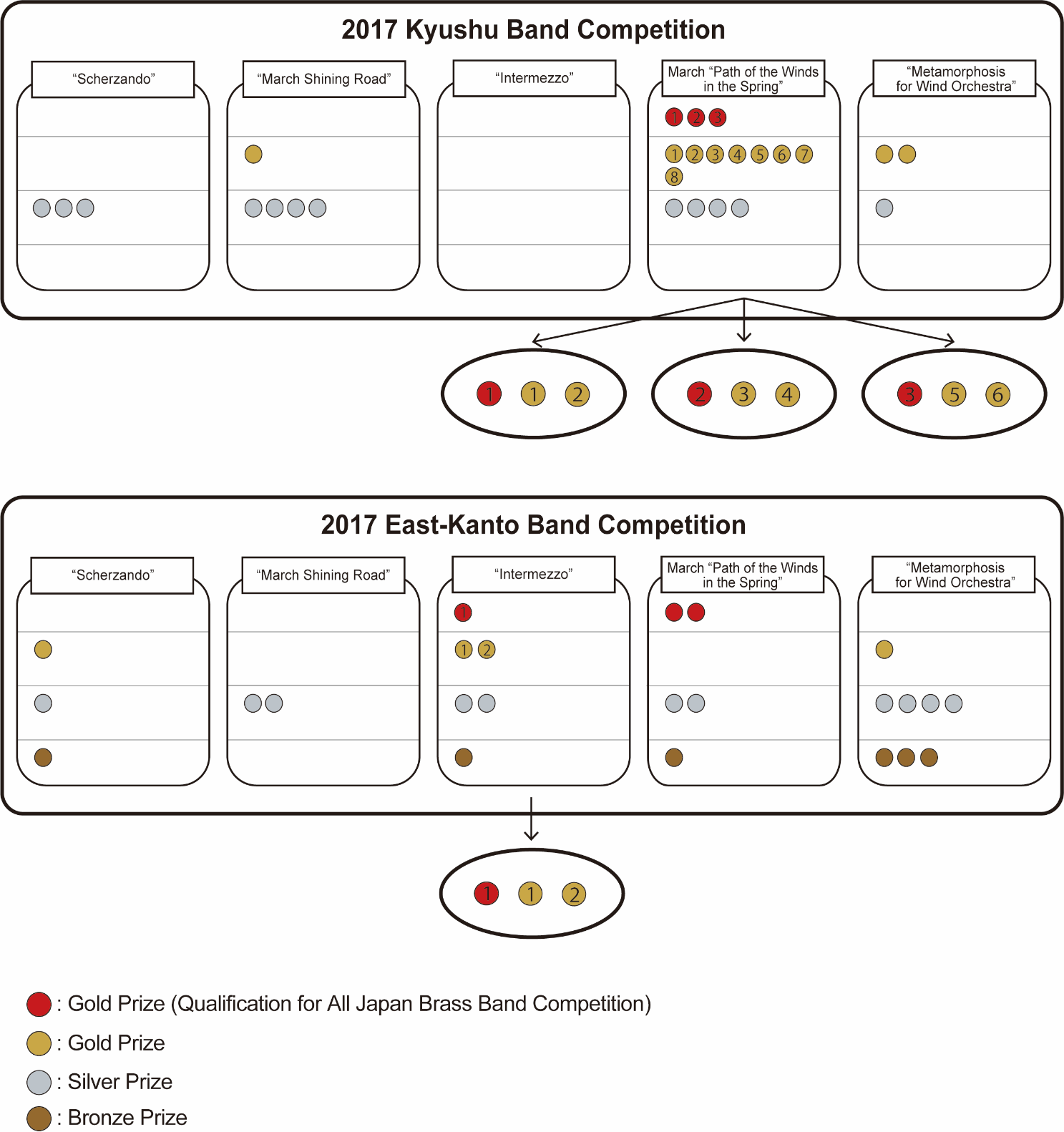


This figure represents the actual results of each regional competition and for all stimulus sets used in this study. Dots representing individual brass bands for each assigned piece, with colors indicating competition results (red for gold award winners advancing to the national competition, gold for those winning gold but not advancing, silver for silver award, and bronze for bronze award). Stimulus sets were created only when, among the groups that performed the same assigned piece at the same regional competition, there was one red dot representing a qualified group and two gold dots representing gold prize groups.

**S2 Text. Subjective Impression of the Importance of Audiovisual Information.**

Subjective impressions of the importance of audiovisual information in judging brass band competitions were investigated. In a previous study, the majority of participants surveyed responded that auditory information was more important in judging music competitions (see Tsay [1]). We investigated whether auditory information was also subjectively perceived as more important than visual information in judging brass band competitions. We asked the participants "Which do you think is more important in judging brass band competitions, auditory information or visual information?" in the questionnaire that preceded the performance evaluation. The participants were asked to choose between auditory and visual information. Chi-square tests were conducted for BMs, NBMs, and NMs based on the participants’ responses.

According to the results, 92.98 % of BMs indicated that audio information was more important, and 7.02 % indicated that visual information was more important (χ^2^ (1) = 126.37, p < 0.001, φ = 0.86). Further, 87.18 % of NBMs indicated that audio information was more important, and 12.82 % indicated that visual information was more important (χ^2^ (1) = 43.13, p < 0.001, φ = 0.744). All NMs indicated that audio information was more important than visual information (χ^2^ (1) = 52.00, p < 0.001, Ï † = 1.00).

Consistent with Tsay’s [1] report, the subjective impression that auditory information is more important than visual information in the judgment of brass band competitions, regardless of judges’ brass band or musical experiences, was confirmed

**Fig. S2 Comparison of the Selection Rate of the Importance of Audio and Visual Information in Judging Brass Band Competitions.**

**
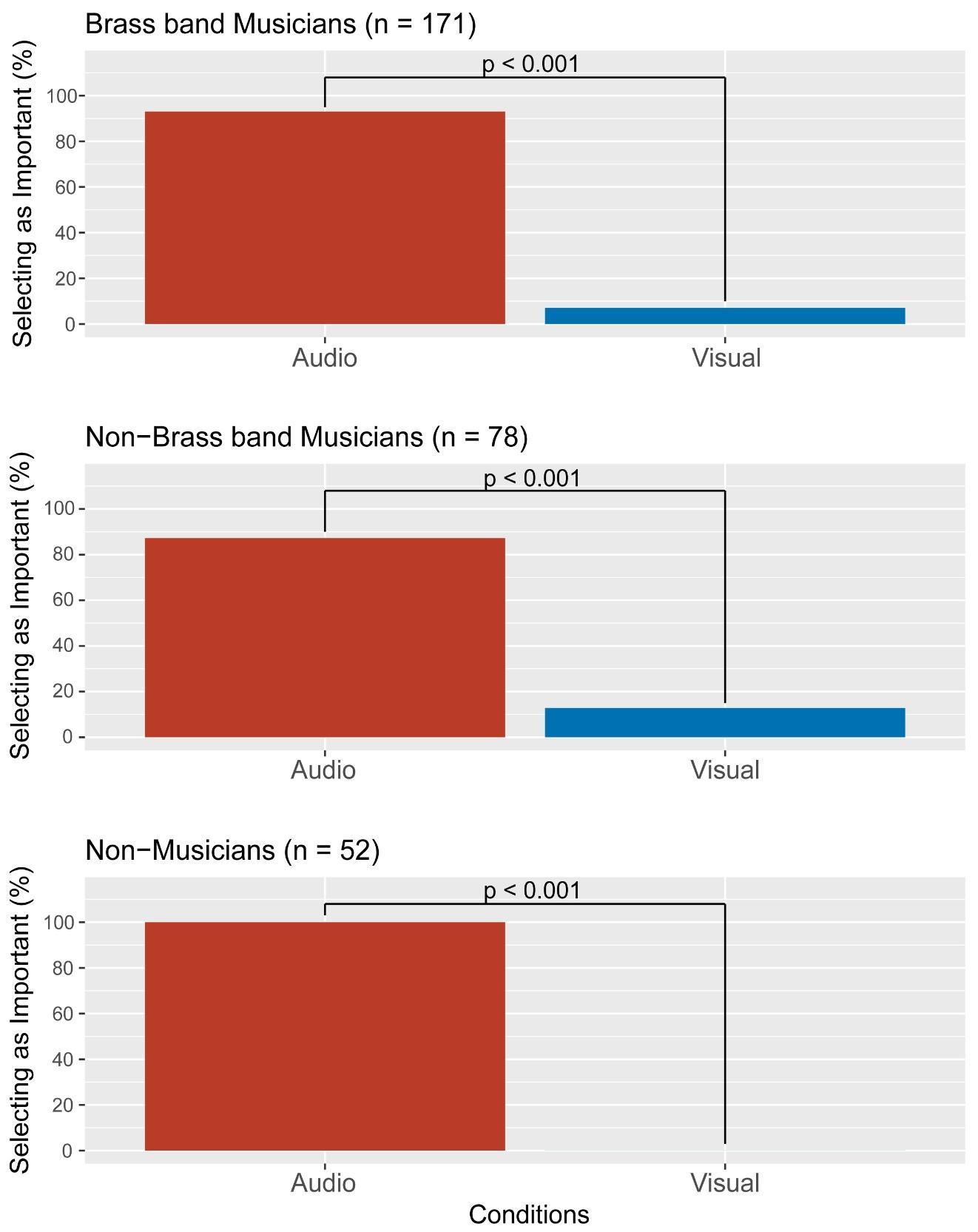
**

These graphs respectively represent comparisons of the selection rates of the importance of audio and visual information in the judging brass band competitions among brass band musicians (top), non-brass band musicians (middle), and non-musicians (bottom).

**Table S1. Results of the Shapiro-Wilk test and the values of kurtosis and skewness for assessing the normality of data.**

|  |  | **ALLs** | **BMs** | **NBMs** | **NMs** |
| --- | --- | --- | --- | --- | --- |
| AV condition | Skewness | 0.27 | 0.26 | 0.26 | 0.22 |
|  | Kurtosis | -0.12 | -0.79 | -0.79 | -1.29 |
|  | Shapiro-Wilk test | *W* = 0.95,  *p* < 0.001 | *W* = 0.94,  *p* = 0.045 | *W* = 0.92,  *p* = 0.045 | *W* = 0.93,  *p* = 0.35 |
| VO condition | Skewness | 0.12 | 0.34 | 0.006 | 0.22 |
|  | Kurtosis | -0.72 | -0.40 | -1.42 | -1.29 |
|  | Shapiro-Wilk test | *W* = 0.96,  *p* = 0.003 | *W* = 0.96,  *p* = 0.037 | *W* = 0.89,  *p* = 0.022 | *W* = 0.95,  *p* = 0.40 |
| AO condition | Skewness | 0.16 | > -0,001 | 0.11 | 0.0092 |
|  | Kurtosis | -0.11 | 0.24 | -1.10 | -0.82 |
|  | Shapiro-Wilk test | *W* = 0.96,  *p* < 0.001 | *W* = 0.96,  *p* = 0.064 | *W* = 0.92,  *p* = 0.015 | *W* = 0.93,  *p* = 0.14 |
