# Supplementary material for: Sight-over-sound effect depends on interaction between evaluators’ musical experience and auditory-visual integration: An examination using Japanese brass band competition recordings
Source: PLoS One. 2025 Apr 29;20(4):e0321442. doi: 10.1371/journal.pone.0321442 (PMC12040236; doi:10.1371/journal.pone.0321442)
Supplement: Attachment — Submitted filename: Response to Reviewers_ver2.docx [file pone.0321442.s003.docx]

**Manuscript ID:** PONE-D-23-19525

**Type:** Research Article

**Title:** Sight-over-sound effect depends on evaluator’s musical experience: An examination using Japanese brass band competition recordings

**Author:** Samma *et al*

**To:** Dr. Thiago P. Fernandes, Academic Editor PLOS ONE

Dear Dr. Fernandes,

**Re: Reply to reviewers on PONE-D-23-19525**

Thank you very much for your review of our submission, manuscript PONE-D-23-19525 entitled “Replicability of the sight-over-sound effect in the judgement of brass band competitions in Japan”, which we submitted to PLoS ONE.

In this letter we summarize how we addressed the comments made by the reviewers. We found the reviews to be extremely helpful, and have done our best to utilize them to strengthen the paper.

Below, we have provided a detailed explanation of how we addressed each of the comments made by the reviewers.

We greatly appreciate your efforts as an editor of the journal and your contribution to improving our manuscript.

Thank you very much.

Tomohiro Samma, Kazuaki Honda, and Shinya Fujii PhD

**Journal requirements:**

When submitting your revision, we need you to address these additional requirements.

1. Please ensure that your manuscript meets PLOS ONEs style requirements, including those for file naming. The PLOS ONE style templates can be found at

https://journals.plos.org/plosone/s/file?id=wjVg/PLOSOne_formatting_sample_main_body.pdf and

https://journals.plos.org/plosone/s/file?id=ba62/PLOSOne_formatting_sample_title_authors_affiliations.pdf

**Author reply:** We have prepared the revised manuscript in accordance with PLOS ONE's style requirements, including the rules for file naming.

2. We note that the grant information you provided in the Funding Information and Financial Disclosure　sections do not match.

When you resubmit, please ensure that you provide the correct grant numbers for the awards you received for your study in the Funding Information section.

**Author reply:** Thank you for pointing that out. We have corrected the information to ensure consistency between the "Funding Information" and "Financial Disclosure" sections.

3. Thank you for stating the following in the Competing Interests section:

K.H. is employed by the NTT Communication Science Laboratories, Nippon Telegraph and Telephone Corporation, Japan. The remaining author has no relevant financial or non-financial interests to disclose.　S.F. has received a Grant-in-Aid for Young Scientists B and a Grant-in-Aid for Scientific Research B from JSPS and research grants from JST COI-NEXT and Keio University Academic Development Funds. S.F. has also received research support from no new folk studio Inc. and Odakyu Railway within the past three years.

Please confirm that this does not alter your adherence to all PLOS ONE policies on sharing data and materials, by including the following statement: This does not alter our adherence to PLOS ONE policies on sharing data and materials. (as detailed online in our guide for authors　http://journals.plos.org/plosone/s/competing-interests). If there are restrictions on sharing of data and/or materials, please state these. Please note that we cannot proceed with consideration of your article until this information has been declared.

Please include your updated Competing Interests statement in your cover letter; we will change the online submission form on your behalf.

**Author reply:** Thank you for your guidance. We confirm that these competing interests do not alter our adherence to all PLOS ONE policies on sharing data and materials.

4. We note that you have indicated that data from this study are available upon request. PLOS only allows data to be available upon request if there are legal or ethical restrictions on sharing data publicly. For more information on unacceptable data access restrictions, please see http://journals.plos.org/plosone/s/data-availability#loc-unacceptable-data-access-restrictions.

In your revised cover letter, please address the following prompts:

a) If there are ethical or legal restrictions on sharing a de-identified data set, please explain them in detail (e.g., data contain potentially sensitive information, data are owned by a third-party organization, etc.) and who has imposed them (e.g., an ethics committee). Please also provide contact information for a data access committee, ethics committee, or other institutional body to which data requests may be sent.

b) If there are no restrictions, please upload the minimal anonymized data set necessary to replicate your study findings as either Supporting Information files or to a stable, public repository and provide us with the relevant URLs, DOIs, or accession numbers. For a list of acceptable repositories, please see http://journals.plos.org/plosone/s/data-availability#loc-recommended-repositories.

We will update your Data Availability statement on your behalf to reflect the information you provide.

**Author reply:** Thank you for your guidance. In this study, we used video recordings of brass band competitions, and the rights to these videos are owned by a third-party organization (All Japan Band Association). Also, videos contain identifiable information, raising the possibility that individual could be recognized from the footage. Therefore, we do not have permission to broadly publicize any part or all of the stimuli, and we have adopted a format where the stimuli are made available only upon request to the authors. We would appreciate your advice if there are any issues with this approach.

5. We note that Figure 1 in your submission contain copyrighted images. All PLOS content is published under the Creative Commons Attribution License (CC BY 4.0), which means that the manuscript, images, and Supporting Information files will be freely available online, and any third party is permitted to access, download, copy, distribute, and use these materials in any way, even commercially, with proper attribution. For more information, see our copyright guidelines: http://journals.plos.org/plosone/s/licenses-and-copyright.

We require you to either (1) present written permission from the copyright holder to publish these figures specifically under the CC BY 4.0 license, or (2) remove the figures from your submission:

a. You may seek permission from the original copyright holder of Figure 1 to publish the content specifically under the CC BY 4.0 license.

We recommend that you contact the original copyright holder with the Content Permission Form (http://journals.plos.org/plosone/s/file?id=7c09/content-permission-form.pdf) and the following text:

I request permission for the open-access journal PLOS ONE to publish XXX under the Creative Commons Attribution License (CCAL) CC BY 4.0 (http://creativecommons.org/licenses/by/4.0/). Please be aware that this license allows unrestricted use and distribution, even commercially, by third parties. Please reply and provide explicit written permission to publish XXX under a CC BY license and complete the attached form.

Please upload the completed Content Permission Form or other proof of granted permissions as an Other file with your submission.

In the figure caption of the copyrighted figure, please include the following text: Reprinted from [ref] under a CC BY license, with permission from [name of publisher], original copyright [original copyright year].

b. If you are unable to obtain permission from the original copyright holder to publish these figures under the CC BY 4.0 license or if the copyright holder requirements are incompatible with the CC BY 4.0 license, please either i) remove the figure or ii) supply a replacement figure that complies with the CC BY 4.0 license. Please check copyright information on all replacement figures and update the figure caption with source information. If applicable, please specify in the figure caption text when a figure is similar but not identical to the original image and is therefore for illustrative purposes only.

**Author reply:** Thank you for your guidance. We have created and replaced the previous figure with an alternative one that complies with the CC BY 4.0 license. The replaced figure does not contain any copyright-protected information and consists entirely of illustrations created by the authors.

6. Please include captions for your Supporting Information files at the end of your manuscript, and update any in-text citations to match accordingly. Please see our Supporting Information guidelines for more information: http://journals.plos.org/plosone/s/supporting-information.

**Author reply:** Thank you for your guidance. We have updated the caption information accordingly.

**Editor's commentary**: This is a very interesting study, and the reading, for most parts, was fast-paceded. I appreaciated the text; however, as I read through it, also I noticed some aspects that could be refined. Hope the authors find these comments, and all of the raised points, helpful in some way.

**Author reply:** Thank you for your encouraging words and the recognition of the value of our study. We are pleased to hear that you found the reading largely fast-paced and appreciable. Your feedback is invaluable, and we are dedicated to addressing all the points raised to refine the quality of the manuscript. We genuinely appreciate the constructive nature of your comments and are confident they will serve to improve the work.

**Editor's commentary**: Abstract

Some sentences in the Abstract require a little bit of work to enhance clarity and flow. For example, sentences like "a crucial ...," "background: brass band...," or "that the dominance" can be rephrased. Also I think the authors can set more details into their sample (age SD) and the stats values in Abstract;

**Author reply:** Thank you. We have rephrased the sentences you pointed out. Also, we have included more detailed information about the participants and statistical values in Abstract.

**Author action:**

Revised abstract: The sight-over-sound effect refers to the dominance of visual over auditory information in judging musical performances. Several studies have pointed out difficulties in replicating the sight-over-sound effect. Previous research has not conclusively determined if this effect persists when controlling for both the music piece and camera angles of the stimuli being compared. Additionally, the relationship between the effect and evaluators’ musical experience remains unclear. To address these gaps in current knowledge, we examined the replicability of the sight-over-sound effect using recordings of Japanese brass band competitions. A total of 301 adults (average age = 34.81 ± 11.71 years) participated in this study, and they were divided into three groups based on their experience with brass band and music. Among the participants, 171 were brass band musicians (n = 171; average age = 33.08 ± 11.57 years), 78 were non-brass band musicians (n = 78; average age = 35.39 ± 13.03 years), and 52 were non-musicians (n = 52; average age = 39.15 ± 8.73 years). We found that the sight-over-sound effect was notably present in non-brass band musicians (Kruskal-Wallis test: p < 0.001), but not in brass band musicians (p = 0.48) or non-musicians (p = 0.37). Thus, the sight-over-sound effect was limited and dependent on the evaluators’ musical experience. Our findings suggest that the evaluator's musical experience should be considered when investigating the sight-over-sound effect.

**Editor's commentary**: Introduction

- The section has clarity and a swift pace, but there are opportunities for additional information, (a) explain how the study builds upon past findings and addresses their limitations, (b) a concise overview of findings, supported by data and their specifics (e.g. you can calculate ORs or even just provide data from the studies), and (c) emphasise the relevance of your study;

**Author reply:** We appreciate the positive remarks on clarity and pace. To address your suggestions (a) – (c), we revised our introduction as follows:

**Author action:**

We added the sentences to explain (a) how the study builds upon past findings and addresses their limitations and (b) a concise overview of findings, supported by data and their specifics as follows.

Line 57-68: Regardless of musical experience, people consistently reported that auditory information is a more important source than visual information when evaluating musical performances (see Experiment 1 in [1]). Experiment 3 in [1] was conducted where participants watched 6-second excerpts of the 1st, 2nd, and 3rd-place performers’ recordings in international music competitions and were asked to select the actual winner from among them (chance level: 33.3%). The results reported that non-musicians were most accurate in correctly identifying the actual winner when evaluating based solely on visual-only (VO) stimuli (mean answering rate: 46.4%), compared to evaluating based on audio-only (AO) stimuli (28.8％) or audiovisual (AV) stimuli (35.4％). Similarly, it was also reported that even professional musicians were able to select the winner with high accuracy when evaluation based on VO stimuli (47.0%), as compared to AO stimuli (25.7%) and AV stimuli (29.5% see Experiment 5 in [1]).

Line 73-78: Mehr et al. [22] demonstrated that the sight-over-sound effect was replicable when using the same stimuli presented in Tsay’s [1] study. However, when the experiment was modified to a two-choice format (chance level: 50%), using a combination of performance stimuli from the winner and a preliminary round loser, the sight-over-sound effect was not observed (VO: 45.2%, AO; 68.4%, AV: 63.6%, see Experiment 3 in [22]).

We also added the sentences to emphasize (c) the relevance of our study.

Line 144-159: The first hypothesis was based on the prediction by Mehr et al. [22] that the sight-over-sound effect would not be replicated when controlling for stimulus information such as camera angles and musical pieces. In the previous studies by Tsay [1, 20] and Mehr et al. [22], it was reported that the accuracy rate in visual-only condition was significantly higher than chance level. In the current study, where the camera angle and musical pieces of the stimuli were controlled, it was hypothesized that the variability in visual information would be reduced, potentially leading to a lower accuracy rate in visual-only condition. We aimed to verify that the sight-over-sound effect is not replicated in the choice of winners by all participants, regardless of their brass band or musical experiences. The second hypothesis is based on the possibility that BMs, utilizing their brass band experience, might be able to select the actual winners with higher accuracy in audio-only and audiovisual conditions as compared to visual-only conditions. The stimuli used in our study consist of performance recordings with the same musical pieces, which may facilitate sound-based evaluations compared to previous studies where the musical pieces were inconsistent. It is hypothesized that BMs, drawing on their brass band experiences, can delicately evaluate the sound and predict the winners, potentially leading to a non-replicability of the sight-over-sound effect.

**Editor's commentary**: - The second paragraph effectively illustrates previous findings but would benefit from offering more interpretations and explaining how these findings built the design or rationale;

**Author reply:** We revised the manuscript to document interpretations of each prior study on the sight-over-sound effect. Furthermore, the challenges posed by these prior studies, as well as the improvements made in our research, are detailed in the third and fourth paragraphs.

**Author action:** We have documented the following interpretations for each prior study.

Line 69-72 (Tsay 2013): These findings highlighted that while there is an acknowledgment of sound being the most crucial source of information in the evaluation of music performances, there was also a natural and automatic reliance on visual cues, occurring subconsciously [1].

Line 78-80 (Mehr 2018): These results suggested that the sight-over-sound effect may not be replicable when differences in the skill level of the performances being compared can be adequately judged through auditory perception alone.

Furthermore, at the end of the second paragraph, we have discussed the sight-over-sound effect from a broader perspective.

Line 83-90: In summary, there is a subjective influence that musical performances should be evaluated with an emphasis on sound. However, if the sight-over-sound effect applies to actual competitions, it is conceivable that different winners may be chosen based on the availability of visual information, especially when the skill levels of the compared performers are close. This implies that performers should be mindful that evaluators’ judgements can be significantly influenced by performers’ visual cues and preferences, necessitating training not only in the musical aspects but also in engaging the evaluators visually. The sight-over-sound effect could potentially overturn the traditional concept of music as an ‘art of sound’.

**Editor's commentary**: - Also, in the third paragraph, place the issues to be investigated in a broader context, underlining their importance;

**Author reply:** Following your comment, we revised the paragraph to describe the relationship between the sight-over-sound effect and the evaluators' musical experience within a broader context.

**Author action:** We have added the following interpretations.

Line 111-126: Third, the details of the evaluator's musical experience should be considered when examining the sight-over-sound effect. Previous studies have shown that this effect occurs regardless of musical experience, but the way musical expertise is assessed varies. For example, Tsay [1] used professional orchestra musicians to evaluate piano performances, while Mehr et al. [22] identified participants with high Musical Ear Test [26] scores as having high musical expertise, despite not having a group with extensive musical training. A key issue is the mismatch between the evaluators' musical expertise and the genre of the performance being assessed. Research indicates that long-term music training leads to brain changes specific to the instrument played, affecting cognitive functions and skills [27-30]. Therefore, even with a background in long-term music training, experiences with different instruments or genres could influence performance preferences and evaluations. Evaluators' musical backgrounds may also affect how they perceive visual aspects of performances. Waddell and Williamon [9] found that musicians were harsher in their ratings than non-musicians, particularly towards performers with poor entrance attitudes or negative facial expressions during mistakes. This highlights the need to consider musical experience when studying the sight-over-sound effect.

**Editor's commentary:** - Clarify the relevance and implications of studying the s-o-s-e. Why is this relevant? Authors need to place more value in their study;

**Author reply:** We revised our manuscript to clarify the value of our research by specifically explaining how using recorded footage from Japanese brass band competitions addresses unresolved challenges in previous studies investigating the sight-over-sound effect.

**Author action:** We revised following paragraphs in Introduction section.

Line 127-138: Consequently, we aimed to investigate the replicability of the sight-over-sound effect in the judgment of Japanese brass band competitions in controlled conditions. To address the three issues stated above, we determined that recordings from Japanese brass band competitions would be ideal for testing the replicability of the sight-over-sound effect. In the Japanese brass band competitions, there were assigned pieces that all participating bands perform, allowing for the controlling camera angle and musical pieces in the compared performances. This enabled us to address the issue of controlling camera angle and musical piece, which remained unresolved in previous studies. Additionally, by using brass band competition recordings, we were able to compare not only between musicians and non-musicians (NMs) but also between brass band musicians (BMs) and non-brass band musicians (NBMs) in their choice of winners. This allowed us to investigate the replicability of the sight-over-sound effect when musical expertise matches the genre of the stimulus.

**Editor's commentary**: - Consider reframing 4-5s paragraphs to ensure smoother transitions and clarity;

**Author reply:** We made changes to improve the readability of the relevant sections.

**Editor's commentary**: - Explain how previous studies on AVP contribute to understanding the sight-over-sound effect;

**Author reply:** We have taken into account all of your comments and those of reviewers and decided to omit the discussions related to previous studies on AVP and the sight-over-sound effect to enhance the coherence of the Introduction section.

**Editor's commentary**: - Consider exploring theoretical frameworks that can elucidate the influence of musical experience on AVs;

**Author reply:** We added theoretical flameworks that can elucidate the influence of musical experience on AVs based on previous studies.

**Author action:** We added following sentences in Introduction section.

Line 122-126: Evaluators' musical backgrounds may also affect how they perceive visual aspects of performances. Waddell and Williamon [9] found that musicians were harsher in their ratings than non-musicians, particularly towards performers with poor entrance attitudes or negative facial expressions during mistakes. This highlights the need to consider musical experience when studying the sight-over-sound effect.

**Editor's commentary**: - Ensure logical coherence in the hypotheses, particularly in explaining how the sound-over-sight effect is 'dependent' on musical experience and its implications. Overall, the authors need to provide what they expected;

**Author reply:** Considering your feedback, we have refined our explanations regarding the sight-over-sound effect and musical experience to ensure logical coherence in the hypothesis.

**Author action:**

We have described the necessity to investigate the relationship between the sight-over-sound effect and musical experience as follows.

Line 111-126: Third, the details of the evaluator's musical experience should be considered when examining the sight-over-sound effect. Previous studies have shown that this effect occurs regardless of musical experience, but the way musical expertise is assessed varies. For example, Tsay [1] used professional orchestra musicians to evaluate piano performances, while Mehr et al. [22] identified participants with high Musical Ear Test [26] scores as having high musical expertise, despite not having a group with extensive musical training. A key issue is the mismatch between the evaluators' musical expertise and the genre of the performance being assessed. Research indicates that long-term music training leads to brain changes specific to the instrument played, affecting cognitive functions and skills [27-30]. Therefore, even with a background in long-term music training, experiences with different instruments or genres could influence performance preferences and evaluations. Evaluators' musical backgrounds may also affect how they perceive visual aspects of performances. Waddell and Williamon [9] found that musicians were harsher in their ratings than non-musicians, particularly towards performers with poor entrance attitudes or negative facial expressions during mistakes. This highlights the need to consider musical experience when studying the sight-over-sound effect.

Additionally, we have added information about the expected results due to differences in musical experience.

Line 152-159: The second hypothesis is based on the possibility that BMs, utilizing their brass band experience, might be able to select the actual winners with higher accuracy in audio-only and audiovisual conditions as compared to visual-only conditions. The stimuli used in our study consist of performance recordings with the same musical pieces, which may facilitate sound-based evaluations compared to previous studies where the musical pieces were inconsistent. It is hypothesized that BMs, drawing on their brass band experiences, can delicately evaluate the sound and predict the winners, potentially leading to a non-replicability of the sight-over-sound effect.

**Editor's commentary**: Methods

- Please extend and detail your eligibility criteria. For instance, include specific conditions or criteria for exclusion, with references to their assumptions;

**Author reply:** We extended and detailed our eligibility criteria.

**Author action:** We added following sentence in Participants subsection.

Line 175-177: Participants were individuals aged 18 and over who are native Japanese speakers, without any hearing or visual impairments, and who read and consented to the online experiment.

**Editor's commentary**: - Describe the efforts made to ensure the representativeness of the sample for better generalisability. I understand this is not the main point of the text, but this could be really important for other researchers and readers;

**Author reply:** In our study, participants were recruited from Keio University and its surrounding areas, utilizing a combination of social media and word-of-mouth advertising. This method aligns with the recent replication experiment of the Sight-over-sound effect by Chiba et al., 2023, and is consistent with the participant recruitment methods and efforts to ensure sample representativeness used in prior research.

**Author action:** We added the following sentence in the participants subsection.

Line 177-180: Participants were recruited from Keio University and its surrounding areas, utilizing a combination of social media and word-of-mouth advertising. We endeavored to ensure a balanced representation of individuals with brass band and music experience by broadly soliciting participants.

**Editor's commentary**: - Address potential biases or limitations in the procedure, as these may impact the interpretation of results;

**Author reply:** To address potential biases or limitations in our study’s procedure, we have included discussions on the limitations arising from (i) employing a between-subjects design, and (ii) the variability among brass band musicians.

**Author action:** We have added the sentences in the Limitations section as follows:

Line 598-614: We consider that there are at least three limitations in this study. First, we did not employ a within-subjects design but a between-subjects design. We employed the between-subjects design to minimize learning and transfer effects that might arise from participants engaging with multiple experimental conditions. However, using a between-subjects design entails the possibility that participants who could achieve high accuracy rates in any condition were coincidentally assigned to the conditions with higher accuracy rates. Additionally, as the experiment was conducted online, the experimental conditions could have varied for each participant, and those who had better experimental condition might have been coincidentally assigned to different conditions. Taken together, it is important to test if the results in this study can be replicated using within-subjects designs in future studies.

The second limitation of this study is the variability of the age at which musical training began among BMs and NBMs. There was considerable variation in the onset age of musical training among participants in this study. Previous studies have reported structural and functional plastic changes in the brain due to music training in early childhood [44,45]. While our study did not consider the onset age or duration of music training, investigating the relationship between such musical backgrounds and the Sight-over-sound effect may lead to a deeper discussion on the impacts of musical experience.

**Editor's commentary**: - Please avoid placing stats outcomes within this section;

**Author reply:** We removed the stats into the beginning of the Results section.

**Author action:** We added the stats outcomes regarding the Intergroup comparisons for demographic and musical training characteristics as follows:

Line 309-313:

There was no significant difference in duration of musical training between the BMs and NBMs (W = 5311, p = 0.63, r = 0.033). BMs and NBMs showed no significant differences in the age at which their music training commenced (BMs: mean = 7.51, SD = 49.2, range = 2−34 years; NBMs: mean = 7.35 years, SD = 5.10, range 2−28 years; W = 4664.5, p = 0.26, r = 0.078). Eleven BMs and 10 NBMs participated in musical competitions.

**Editor's commentary**: - Actually, the authors could consider examine skewness and kurtosis as most important parameters to normality;

**Author reply:** Considering your comment, we included the kurtosis and skewness as parameters of normality in our paper. Previous research indicates that absolute values of skewness and kurtosis less than 1.0 suggest slight non-normality, while values above 1.0 indicate moderate non-normality (Lei and Lomax, 2005). We have decided to present the kurtosis and skewness alongside the Shapiro-Wilk test results in the Supplementary Information.

**Author action:** We added the following Table S1 in the Supplementary Information.

**Table S1. Results of the Shapiro-Wilk test and the values of kurtosis and skewness.**

|  | AO condition | | | VO condition | | | AV condition | | |
| --- | --- | --- | --- | --- | --- | --- | --- | --- | --- |
| Group | Shapiro-Wilk test | Kurtosis | Skewness | Shapiro-Wilk test | Kurtosis | Skewness | Shapiro-Wilk test | Kurtosis | Skewness |
| ALLs | *W* = 0.96, *p* < 0.001 | -0.11 | 0.16 | *W* = 0.96, *p* = 0.003 | -0.72 | 0.12 | *W* = 0.95, *p* < 0.001 | -0.12 | 0.27 |
| BMs | *W* = 0.96, *p* = 0.064 | 0.24 | > -0.001 | *W* = 0.96, *p* = 0.037 | -0.40 | 0.34 | *W* = 0.94, *p* = 0.045 | -0.79 | 0.26 |
| NBMs | *W* = 0.92, *p* = 0.015 | -1.10 | 0.11 | *W* = 0.89, *p* = 0.022 | -1.42 | 0.006 | *W* = 0.92, *p* = 0.045 | -0.79 | 0.26 |
| NMs | *W* = 0.93, *p* = 0.14 | -0.82 | 0.0092 | *W* = 0.95, *p* = 0.40 | -1.29 | 0.22 | *W* = 0.93, *p* = 0.35 | -1.29 | 0.22 |

**Editor's commentary**: - Provide effect sizes and CIs (i.e. I think you can use MBESS or even the rcompanion for R);

**Author reply:** Considering your comment, we calculated the confidence intervals and the effect size of the Kruskal-Wallis test using the “rstatix” package (<https://search.r-project.org/CRAN/refmans/rstatix/html/kruskal_effsize.html>).

**Author action:**

We revised the following sentences in the results section to include the Cis and effect sizes.

Line 361-363: The Kruskal−Wallis test showed significant differences between conditions (H(2) = 16.93, p < 0.001, η2 ＝ 0.53, 95% CI for effect size: [0.06 0.39]); …

Line 401-403: The Kruskal-Wallis test revealed significant differences in the percentage of correct responses (H(2) = 9.32, p = 0.009, η2 ＝ 0.072, 95% CI for effect size: [0.0018 0.22]; see top plot of Fig.6), …

Line 416-417: The Kruskal-Wallis test revealed significant differences in the percentage of correct responses (H(2) = 7.32, p = 0.026, η2 ＝ 0.057, 95% CI for effect size: [0 0.21];see middle plot of Fig.7), …

**Editor's commentary**: Discussion

- Consider reframing paragraphs 2 and 3 to refine readability;

**Author reply:** We revised the paragraphs to improve the readability of the relevant sections.

**Editor's commentary**: - Debate on stimulus control can be more concise;

**Author reply:** We have made revisions to our manuscript to present the discussion on stimulus control in a more concise manner.

**Author action:** We have made the following revisions:

Line 467-479: In our study, we used recordings with controlled camera angles for all performances, addressing the issue by Mehr et al. [22] who proposed that varying camera angles might influence the sight-over-sound effect. Our findings support their proposition, indicating that controlled camera angles, which reduce visual disparities, could diminish the sight-over-sound effect. This contrasts with previous studies where varied camera angles were used [1,20,22,24]. Additionally, unlike these studies that used stimuli which performed different musical pieces, our study used the same pieces performed by different brass band groups. Controlling musical pieces likely led to more consistent player movements, potentially reducing the sight-over-sound effect. In contrast, the use of varied musical pieces in previous studies might have resulted in more diverse player movements due to differences in melodies, harmonies, and rhythms, possibly enhancing the ability to discern between winning and losing performances. Thus, we suggest that both camera angles and musical piece selection are key factors in explaining the sight-over-sound effect.

**Editor's commentary**: - Please check "variations in performance level" and link to your main rationale;

**Author reply:** We revisited the “variations in performance level” in light of previous research and included a discussion.

**Author action:** We have added the sentences as follows.

Line 482-493: Mehr et al. highlighted that the degree of variation in performance level is a significant factor influencing the sight-over-sound effect [22]. When the variation was small, results reported a visual dominance, such as when evaluating the top three performers in international-level competitions [1,20]. Conversely, when the variation was large, auditory dominance was observed, such as when comparing competition winners to competitors eliminated in earlier rounds [22]. Recently, Chiba et al. [23] proposed that the impact of variation in performance level may be genre-dependent. For instance, when the variation was small, visual dominance was observed in Western-classical solo music competitions, while auditory dominance was noted in Japanese-Shamisen solo music competitions [23]. Chiba et al. suggest that such differences in the dominance of auditory and visual cues may be observed due to movements induced by music genres from different cultures, such as Western classical music and Japanese shamisen music.

**Editor's commentary**: - Consider extend the debate on theories and your findings;

**Author reply:** We extended the debate on theories and our findings in the Discussion section.

**Reviewers’ Comments to the Author:**

**Reviewer #1:** This study is well written and deals with an important topic. However, the rationales are weak to support the hypotheses and some of the findings are explained in a vague manner without evidence. This part should be improved before accepted for publication. Specific comments are as follows.

**Author reply:** Many thanks for providing useful comments. We did our best to revise our paper.

**Author action:** Please see our replies below.

**Reviewer #1:** Line 107: The ‘sound-over-sight effect’ seems to be an error.

**Author reply:** We corrected the sentence.

**Author action:** Please see the corrected sentence below.

Line 142-143: H2: BMs do not exhibit the sight-over-sound effect due to their ability to delicately evaluate the sounds and accurately predict the winners.

**Reviewer #1:** Line 108-111: I suggest further explaining the rationale for H1 and H2. Readers may wonder the specific reasoning behind why the sight-over-sound effect is not replicable in the judgement of Japanese brass band competitions when camera angles and music pieces are controlled and why the sight-over-sound effect is dependent on the evaluator’s musical experience. The authors provided a citation for each hypothesis but specifying those reasoning may improve the quality of this manuscript.

**Author reply:** Following your suggestions, we have added further explaining the rationale for H1 and H2 in the hypothesis section.

**Author action:** We have added following paragraph.

Line 144-159: The first hypothesis was based on the prediction by Mehr et al. [22] that the sight-over-sound effect would not be replicated when controlling for stimulus information such as camera angles and musical pieces. In the previous studies by Tsay [1,20] and Mehr et al. [22], it was reported that the accuracy rate in visual-only condition was significantly higher than chance level. In the current study, where the camera angle and musical pieces of the stimuli were controlled, it was hypothesized that the variability in visual information would be reduced, potentially leading to a lower accuracy rate in visual-only condition. We aimed to verify that the sight-over-sound effect is not replicated in the choice of winners by all participants, regardless of their brass band or musical experiences. The second hypothesis is based on the possibility that BMs, utilizing their brass band experience, might be able to select the actual winners with higher accuracy in audio-only and audiovisual conditions as compared to visual-only conditions. The stimuli used in our study consist of performance recordings with the same musical pieces, which may facilitate sound-based evaluations compared to previous studies where the musical pieces were inconsistent. It is hypothesized that BMs, drawing on their brass band experiences, can delicately evaluate the sound and predict the winners, potentially leading to a non-replicability of the sight-over-sound effect.

**Reviewer #1:** Line 134: The word, ‘subdivide’, means dividing something that has already been divided. This may need to be edited.

**Author reply:** We changed the word from 'subdivide' to 'divide'.

**Author action:** Please see the corrected sentence below:

Line 187-189: Based on their music backgrounds, they were divided into three groups: brass band musicians (BMs), non-brass band musicians (NBMs), and non-musicians (NMs; see Table 1).

**Reviewer #1:** Fig 1: I suggest clarifying the award information within performance, set, and group in the figure. It is not easy to digest this figure.

**Author reply:** Thank you for valuable feedback. To provide clearer information, we removed Fig. 1 and incorpolated a description of the stimuli in the main text. We transferred the additional detailed information to S2 text and Fig S2 in the Supplementary Information.

**Author action:** We added following sentences in Stimuli subsection.

Line 212-228: We utilized the performance recordings of 30 high school brass bands participating in Japanese regional brass band competitions (final qualifying rounds) as experimental stimuli. The recordings were organized in a three-choice format, each consisting of the same song performed by three different bands. This resulted in a total of 10 distinct sets, each made up of three groups (see S2 text and Fig S2. for detailed information on the composition of all sets, the competitions of each group's stimuli, and the pieces performed). One of the three brass bands in a set qualified for the All Japan Brass Band Competition. The other two brass bands were placed highly in regional competitions, but did not qualify for national competitions. Mehr et al., [22] reported that the sight-over-sound effect was not observed when comparing stimuli with significant differences in performance skills. Therefore, to minimize the disparity in performance skills, we used groups that received gold awards (about top 30% in the competition) as stimuli rather than silver or bronze award recipients. The musical pieces used as stimuli were selected from past brass band competition assigned pieces from 2012 to 2018. Each year, the brass band association selects five assigned pieces, and all participating brass bands must choose and perform one of these five pieces. Leveraging this characteristic, the three stimuli in a single stimulus set were made to be the same piece performed by three different groups.

**Reviewer #1:** Line 300: The ‘sound-over-sight effect’ seems to be an error.

**Author reply:** We revised the sentence.

**Author action:** Please see the revised sentence below:

Line 447-450: Our secondary hypothesis (H2) proposed that BMs did not exhibit the sight-over-sound effect because of the ability to select the actual winners with higher accuracy in audio-only and audiovisual conditions as compared to visual-only condition based on their brass band experiences.

**Reviewer #1:** Line 397-414: The results of 1-sample Wilcoxon signed-rank tests should be moved to Results section from Discussion.

**Author reply:** We moved the results of the 1-sample Wilcoxon test from the Discussion section to the Results section.

**Author action:** Please see the following sentences in Results section.

Judgement experiment

ALLs

Line 321-326: The results of the 1-sample Wilcoxon signed-rank test showed no significant difference between the mean percentage of correct responses and chance level in both the AO and AV conditions (AO: V = 3405, p = 0.046, r = 0.40; AV: V = 2977, p = 0.079, r = 0.18, respectively). However, a significant difference was observed between the accuracy rate and chance level in the VO condition (V = 3468, p < 0.001, r = 0.40).

BMs

Line 342-346: The results of the 1-sample Wilcoxon signed-rank test showed no significant difference between the mean percentage of correct responses and chance level in both the VO and AV conditions (VO: V = 1053, p = 0.13, r = 0.20; AV: V = 1265, p = 0.021, r = 0.30, respectively). However, a significant difference was observed between the accuracy rate and chance level in the AO condition (V = 1033, p = 0.002, r = 0.44).

NBMs

Line 366-371: The results of the 1-sample Wilcoxon signed-rank test showed no significant difference between the mean percentage of correct responses and chance level in both the AO and AV conditions (AO: V = 190, p = 0.17, r = 0.24; AV: V = 144, p = 0.63, r = 0.10, respectively). However, a significant difference was observed between the accuracy rate and chance level in the VO condition (V = 215, p < 0.001, r = 0.76).

NMs

Line 386-389: The 1-sample Wilcoxon signed-rank test indicated that none of the percentages exceeded the chance level (AO: V = 141, p = 0.38, r = 0.19; VO: V = 137, p = 0.03, r = 0.53; AV: V = 59, p = 0.36, r = 0.25).

**Reviewer #1:** Line 428-439: The interpretation of significant results seem to be conjecture, being described without any prior findings. Please discuss this finding in reference to prior findings.

**Author reply:** Following your comments, we revised the discussion on the results for NBMs and NMs in reference to prior findings. Please see the following two revised paragraphs.

**Author action:**

Discussion on the results for NBMs

Line 579-588: But why could the NBMs identify winners above chance level solely in the VO condition? We assume that the non-brass band musical experience might let the NBMs discern performance quality through the visual appeal of the movements. It is reasonable to assume that NBMs predominantly rely on visual information, influenced by their diverse non-brass band musical background, which aligns with the results of previous studies that have examined the sight-over-sound effect [1,20]. Additionally, in Japanese brass band performances, the visual aspects of a performance are often crucial to positive reception. The groups featured in our stimuli may have been actively engaging the audience with their movements, and such visual strategies might resonate more with NBMs, influencing their assessments.

Discussion on the results for NMs

Line 589-595: No statistical differences were observed between any of the conditions for the NMs. Although no statistical difference was observed in the NMs, there was a tendency for higher accuracy rates in the VO condition compared to chance level (p = 0.03, adjusted α = 0.016). This result suggests that the NMs, like the NBMs, may exhibit a tendency towards visual information dominance. Given their minimal musical experience, the NMs might not have been able to evaluate performances based on the visual appearance of their own musical experiences, as the NBMs could.

**Reviewer #2:** General comments:

The authors present an interesting experimental study on limited replicability of the sight-over-sound effect in the judgment of Japanese brass band performances.

There are no major concerns, but significant improvements are expected on the following points:

* For the Introduction part: an update of the references for the main topic would be useful;

* For the Methods part: although the study is well-designed, some additional details are necessary, in particular for experimental groups and stimuli choice;

* For the Results part: a reorganization of their presentation (currently distributed in different parts of the manuscript) is necessary, as well as a few additional tests;

* For the Discussion part: results analysis is well documented, but would need to be deepened, in particular by addressing the nature of the audiovisual interactions in the judgement task and the potential limitations of the study.

The manuscript is generally well written, but there are still some corrections to be made.

In conclusion, although the results presented may be important for the scientific community working on sensory processing, substantial revision is required before the manuscript can be accepted for publication.

In addition, regarding data availability, the experimental stimuli used are not directly available (but only upon request to the corresponding author). Given PLoS One policy on the matter, I don't know if this will constitute an obstacle to the acceptance of the manuscript.

**Author reply:** We sincerely appreciate your highly valuable comments. In response to your feedback, we have thoroughly revised our paper. The details of our responses to each comment and the corresponding revisions are documented below.

**[Major revisions]**

**Reviewer #2:** 2-1. Introduction:

* Page 3 and following: I suggest to update your references on audiovisual interactions in music experience (e.g.: Moura et al, PLoS One 2023; Moura et al, NPJ Sci Learn 2023; Nusseck et al, Front Psychol 2022; Rozé et al, Sci Rep 2020) and comment the findings of these studies.

**Author reply:** Thank you for your valuable suggestion. After reviewing the content of the prior studies you recommended, we have incorporated them into the Introduction section.

**Author action:** Please see the following sentence:

Line 48-50: In addition, other studies have reported that ancillary gestures which do not directly affect the performance, can influence musical expression [11-14].

**Reviewer #2:** * Page 5, lines 90-91: I suggest that you support your argument with the results of the work of Schneider and his colleagues (Schneider et al, Ann N Y Acad Sci, 2005).

**Author reply:** We have reviewed the work by Schneider and his colleagues, along with several related prior studies, and have added in our revised manuscript.

**Author action:** Please see the following sentences:

Line 118-122: Research indicates that long-term music training leads to brain changes specific to the instrument played, affecting cognitive functions and skills [27-30]. Therefore, even with a background in long-term music training, experiences with different instruments or genres could influence performance preferences and evaluations.

References:

Schneider P, Sluming V, Roberts N, Scherg M, Goebel R, Specht HJ, et al. Structural and functional asymmetry of lateral Heschl’s gyrus reflects pitch perception preference. Nat Neurosci. 2005;8: 1241–1247. doi:10.1038/nn1530

Choi U-S, Sung Y-W, Ogawa S. Brain Plasticity Reflects Specialized Cognitive Development Induced by Musical Training. Cereb Cortex Commun. 2021;2: tgab037. doi:10.1093/texcom/tgab037

Zatorre RJ, Chen JL, Penhune VB. When the brain plays music: auditory-motor interactions in music perception and production. Nat Rev Neurosci. 2007;8: 547–558. doi:10.1038/nrn2152

Schneider, P., Sluming, V., Roberts, N., Bleeck, S., & Rupp, A. Structural, functional, and perceptual differences in Heschl's gyrus and musical instrument preference. Annals of the New York Academy of Sciences. 2005;106: 387–394. doi:10.1196/annals.1360.033

**Reviewer #2:** Methods:

* Participants:

- Page 8, lines 139, 144 and 156: For BMs, could you explain the mismatch between numbers of participants: 55 (line 144) vs 105 (line 156), given their total number, 171 (line 139)?

**Author reply:** Thank you for pointing this out. Upon double-checking, we confirmed that there were indeed 118 BMs with musical experience beyond that of brass band activity. That is, out of the 171 individuals with experience in the brass bands, 118 had experience in other musical domains in addition to the brass band activity.

**Author action:** The text has been revised as follows.

Line 201-203: Among BMs, 118 participants had musical training other than brass bands training (mean = 10.98 years, SD = 8.79, range = 1−48 years, vocal: 6, keyboards: 96, strings: 3, wind instruments: 26, percussion: 2).

We also revised Table 1 as follows:

Table 1

|  | Mean age (years) | Sex (F/M) | Mean duration of musical training (years) | Mean age of commencement of musical training (years) | Mean duration of brass band training (years) | Mean age of commencement of brass band training (years) |
| --- | --- | --- | --- | --- | --- | --- |
| Brass band musicians (n = 171) | 33.08 (11.57, 18-56) | 125/46 | 10.98 (8.79,  1-48) | 7.06  (4.88 1-48) | 8.07 (5.37,  1-35) | 12.25 (1.88, 7-19) |
| Non-brass band musicians (n =78) | 35.39 (13.03, 18-58) | 62/16 | 11.72 (7.81, 1-34) | 7.35 (5.10, 2-28) |  |  |
| Non-musicians (n = 52) | 39.15 (8.73, 19-55) | 44/8 |  |  |  |  |

**Reviewer #2:** - Page 8, lines 148-152: This section should be placed at the beginning of the Results part (“There was no significant difference in…”)

**Author reply:** Following your suggestion, we have placed this section to the beginning of the Results part.

**Author action:** Please see the new section, “Intergroup comparisons for musical training characteristics”, at the beginning of the Results part:

Line 307-313:

Intergroup comparisons for musical training characteristics

There was no significant difference in duration of musical training between the BMs and NBMs (W = 5311, p = 0.63, r = 0.033). BMs and NBMs showed no significant differences in the age at which their music training commenced (BMs: mean = 7.51, SD = 49.2, range = 2−34 years; NBMs: mean = 7.35 years, SD = 5.10, range 2−28 years; W = 4664.5, p = 0.26, r = 0.078). Eleven BMs and 10 NBMs participated in musical competitions.

**Reviewer #2:** * Stimuli:

- Page 9, lines 169-170: I suggest to briefly explain why you have chosen two gold prize awarded brass band, rather than silver or bronze prize awarded ones. This choice could be regarded as “no optimal” for your protocol.

**Author reply:** Thank you for your valuable comments. In the prior research by Mehr et al., it was found that the sight-over-sound effect only occurs when the difference in performance skills between the compared stimuli is small. Therefore, in this study, to minimize the difference in performance skills as much as possible, we used only stimuli of brass bands that won gold awards, avoiding those that received silver or bronze awards.

**Author action:** We added the following sentences.

Line 220-223: Mehr et al., [22] reported that the sight-over-sound effect was not observed when comparing stimuli with significant differences in performance skills. Therefore, to minimize the disparity in performance skills, we used groups that received gold awards (about top 30% in the competition) as stimuli rather than silver or bronze award recipients.

**Reviewer #2:** - Pages 9-10, lines 170-174: Could you specify the whole number of available pieces and brass bands (e.g.: “the 10 experimental sets were chosen among…”)?

**Author reply:** The 10 experimental sets were chosen among recordings of the five regional qualifying rounds of the All Japan Brass Band Competition (specifically, 2012 Hokuriku, 2014 Hokuriku, 2016 Kyushu, 2017 Kyushu, and 2017 East-Kanto). Each year, the brass band association assign five musical pieces, and all participating bands must perform one of these. Among the 20 assigned pieces for the 2012, 2014, 2016, and 2017 competitions, 8 pieces were chosen based on the procedure outlined in S3 text and S2 Fig in Supplementary Information. Furthermore, among the 52 brass bands that received gold awards (approximately the top 30% in the competition), we narrowed down to 30 brass bands that met our criteria (detailed in the subsequent response to your comment).

**Author action:** The method section now includes the following sentence describing the 10 sets of stimuli.

Line 212-228: We utilized the performance recordings of 30 high school brass bands participating in Japanese regional brass band competitions (final qualifying round) as experimental stimuli. The recordings were organized in a three-choice format, each consisting of the same song performed by three different bands. This resulted in a total of 10 distinct sets, each made up of three groups (see S2 text and Fig S2. for detailed information on the composition of all sets, the competitions of each group's stimuli, and the pieces performed). One of the three brass bands in a set qualified for the All Japan Brass Band Competition. The other two brass bands were placed highly in regional competitions, but did not qualify for national competitions. Mehr et al., [22] reported that the sight-over-sound effect was not observed when comparing stimuli with significant differences in performance skills. Therefore, to minimize the disparity in performance skills, we used groups that received gold awards (about top 30% in the competition) as stimuli rather than silver or bronze award recipients. The musical pieces used as stimuli were selected from past brass band competition assigned pieces from 2012 to 2017. Each year, the brass band association selects five assigned pieces, and all participating brass bands must choose and perform one of these five pieces. Leveraging this characteristic, the three stimuli in a single stimulus set were made to be the same piece performed by three different groups.

**Reviewer #2:** - Pages 10, lines 174-175: To be clearer, could you briefly explain (either here or in the Fig 1 caption) the criteria for choosing the experimental sets (i.e.: for a given competition, why only certain musical pieces have been selected?)

**Author reply:** The criteria were to create a set of three bands, consisting of one gold award winner advancing to the national competition and two gold award winners not advancing, with all three bands performing the same designated piece. This approach aimed to minimize variation in performance skills, following the finding of a previous study by Mehr et al. [22], which demonstrated the sight-over sound effect within the small variations in performance skills. We applied these criteria to the recordings from the five regional qualifying rounds of the All Japan Brass Band Competition (namely 2012 Hokuriku, 2014 Hokuriku, 2016 Kyushu, 2017 Kyushu, and 2017 East-Kanto), resulting in the selection of 10 sets as illustrated in Fig S2 (see Supplementary Information). Musical pieces were automatically selected based on this approach from the five regional qualifying rounds.

**Author action:** Please also see the S3 text S2 Fig in Supplementary Information:

S3 text. Criteria for Choosing the Experiment Stimuli.

All the experimental stimuli for this study were sourced from recordings of the final qualifying rounds of the All Japan Brass Band Competition, covering five regional rounds: 2012 Hokuriku, 2014 Hokuriku, 2016 Kyushu, 2017 Kyushu, and 2017 East-Kanto. In Japanese brass band competitions, the Band Association annually selects five assigned pieces, with each participating group having to perform one of five assigned pieces. Leveraging this unique aspect of the Japanese brass band competition, each stimulus set included in our study consisted of three performance recordings from the same regional competition, all performing the same assigned piece. Within each set, one group was a finalist from the qualifying rounds, having advanced to the All Japan Brass Band Competition (usually the top 2-3 groups in regional competition), while the other two achieved gold awards but did not proceed to the national competition (typically the top 30% in each regional competition). The results of these regional competitions and all stimulus sets used in this study are detailed in S2 Fig.

S2 Fig. Structure of Each Experimental Stimulus Sets

This figure represents the actual results of each regional competition and for all stimulus sets used in this study. Dots representing individual brass bands for each assigned piece, with colors indicating competition results (red for gold award winners advancing to the national competition, gold for those winning gold but not advancing, silver for silver award, and bronze for bronze award). Stimulus sets were created only when, among the groups that performed the same assigned piece at the same regional competition, there was one red dot representing a qualified group and two gold dots representing gold prize groups.

**Reviewer #2:** * Procedure (page 11, lines 2016-207): Please briefly specify the type of randomization used.

**Author reply:** Randomization was performed using Qualtrics' Randomizer feature (see https://www.qualtrics.com/support/survey-platform/survey-module/survey-flow/standard-elements/randomizer/).

**Author action:** We have added the sentences as follows:

Line 240-244: This experiment was conducted using the online survey platform Qualtrics (https://www.qualtrics.com/), and the randomization of presentation order was achieved using the randomization methods implemented in Qualtrics.

**Reviewer #2:** 2-3. Results:

* Page 13, lines 239-240: At the beginning of this section, please add a first subpart on intergroup comparisons for demographic and musical training characteristics (currently on page 8, lines 148-152)

**Author reply:** We added the subpart on intergroup comparisons for demographic and musical training characteristics.

**Author action:** Please see the following:

Line 307-313:

Intergroup comparisons for demographic and musical training characteristics

There was no significant difference in duration of musical training between the BMs and NBMs (W = 5311, p = 0.63, r = 0.033). BMs and NBMs showed no significant differences in the age at which their music training commenced (BMs: mean = 7.51, SD = 49.2, range = 2−34 years; NBMs: mean = 7.35 years, SD = 5.10, range 2−28 years; W = 4664.5, p = 0.26, r = 0.078). Eleven BMs and 10 NBMs participated in musical competitions.

**Reviewer #2:** * Judgment experiment:

- For ALLs (page 13, lines 241-246): Did you also assess a potential effect of participants' gender (see, for instance: Bouhuys et al, J Affect Disord 1995)?

**Author reply:** Considering your comment, we assessed a potential effect of participants’ gender as follows:

When comparing the accuracy rates between conditions using data from female participants (n = 231), a there was no significant differences among three conditions in the Kruskal-Wallis test (AO: 35.24±15.96%, VO: 41.22±19.01%, AV: 34.27±15.78%, H(2) = 5.96, p = 0.051, η^2^ = 0.026). Furthermore, we conducted 1-sample Wilcoxon tests, a significant difference was observed between the average rate of VO and chance level (AO: V = 1986. p = 0.19, r = 0.15; VO: V = 2023, p < 0.001, r = 0.40; AV: V =1548, p = 0.52, r = 0.075; adjusted alpha = 0.016).

In the case of male participants (n = 70) , there were no significant differences among three conditions (AO: 36.96±10.63%, VO: 40.43±17.70%, AV: 39.58±13.01%, H(2) = 0.91, p = 0.64, η^2^ = 0.013). Applying the 1-sample Wilcoxon signed-rank test, only the percentage of AV condition was significantly differ from the chance level (AO: V = 208, p = 0.032, r = 0.45; VO: V = 200, p = 0.060, r = 0.39; AV: V = 237, p = 0.013, r = 0.51; adjusted alpha = 0.016).

As there was little observed potential effect between genders, and the impact on the sight-over-sound effect was deemed minimal, we have decided not to incorporate these results into the main text but have included them in the Supplementary Information (see S4 text).

**Reviewer #2:** - For BMs (page 13, lines 253-258): Did you also assess a potential effect of other musical experience (than brass practice) in the participants of this group?

**Author reply:** We assessed a potential effect of other musical experiences (than brass band practice) in the participants of this group as follows:

In this study, there were total of 171 BMs, and 118 of them had not only brass band experience but also experience in other musical genres. Therefore, we subdivided the BMs into participants with both brass band and other musical experiences (n = 118) and those with only brass band experience (n = 53), and compared the accuracy rates between the two subgroups. The results showed no statistically significant differences between the subgroups in any condition (Kruskal-Wallis test: BMs with other musical experience: H(2) = 3.15, p = 0.21, η2 = 0.027; BMs without other musical experience: H(2) = 0.26, p = 0.88, η2 = 0.005). This suggests that the effect of the presence or absence of experience in other musical genres may be minor in BMs.

**Author action:** We added following sentences in the Discussion section.

Line 557-566: In this study, we tested a total of 171 BMs, and 118 of them had not only brass band experience but also experience in other musical genres. This raises the question of whether the presence or absence of experience in other musical genres affect the results in BMs. Therefore, we subdivided the BMs into participants with both brass band and other musical experiences (n = 118) and those with only brass band experience (n = 53), and compared the accuracy rates between the two subgroups. The results showed no statistically significant differences between the subgroups in any condition (Kruskal-Wallis test: BMs with other musical experience: H(2) = 3.15, p = 0.21, η2 = 0.027; BMs without other musical experience: H(2) = 0.26, p = 0.88, η2 = 0.005). This suggests that the effect of the presence or absence of experience in other musical genres may be minor in BMs.

**Reviewer #2:** * Additional analysis:

- For ALLS and the three experimental groups (BMs, NBMs and NMs), could you verify that there are no intragroup differences for the number of participants between the three experimental conditions (AO, VO and AV)?

**Author reply:** There were minor intragroup differences for the number of participants between the three experimental conditions. Specifically, BM group comprised 201 individuals (AO: 52, VO: 58, AV: 61), NBM group included 78 individuals (AO: 32, VO: 21, AV: 25), and NM group had 52 individuals (AO: 21, VO: 18, AV: 13). It’s important to note that the Kruskal-Wallis test is applicable even with unequal sample sizes in the compared groups, as indicated in the original work by Kruskal & Wallis (1952). Therefore, we decided to use the Kruskal-Wallis test in our study despite the varying group sizes in our study. In response to your comment, we recognized the importance of clearly specifying the number of participants for each condition and have therefore included Table 2 in our revised manuscript.

**Author action:** Please see Table 2 in the revised manuscript, which summarizes the number of participants for each experimental condition.

**Table 2. Number of Participants in Each Experimental Conditions**

|  | Audio-only condition | Visual-only condition | Audiovisual condition |
| --- | --- | --- | --- |
| Brass band musicians  (n = 171) | 52 | 58 | 61 |
| Non-brass band musicians (n = 78) | 32 | 21 | 25 |
| Non-musicians  (n = 52) | 21 | 18 | 13 |
| All participants  (N = 301) | 105 | 97 | 99 |

We have also added following sentences in statistical analysis section.

Line 295-299: The number of participants for each experimental condition is detailed in Table 2. To compare the percentage of correct responses between conditions, the Kruskal-Wallis test was used (adjusted α = 0.05). It is important to note that the Kruskal-Wallis test can be utilized even when sample sizes in the compared groups are not equal [31].

**Reviewer #2:** - It would be interesting to provide intergroup comparisons for overall performance and according to conditions (AO, VO, AV).

**Author reply:** In response to your recommendation, we revised the manuscript to include intergroup comparisons by conditions (AO, VO, AV). The analyses yielded the following:

In the AO condition, significant differences were observed among the three groups (H(2) = 9.32, p = 0.009, η^2^ = 0.019). The Steel-Dwass tests showed significant differences between BMs and NBMs (t = 3.03, p = 0.006).

In the VO condition, significant differences were observed among the three groups (H(2) = 7.32, p = 0.026, η^2^ = 0.021). The Steel-Dwass tests showed significant differences between BMs and NBMs (t = 2.58, p = 0.027).

In the AV condition, no significant differences were found among the three groups (H(2) = 1.92, p = 0.38, η^2^ = 0.020)

It is significant to note that the BM group exhibited a higher percentage of correct responses in the AO condition compared to the NBM group, whereas the NBM group had higher percentage of correct responses in the VO condition compared to the BM group.

**Author action:**

Considering the importance of these findings, we have included the results of intergroup comparisons by conditions in the revised manuscript.

Line 396-440:

**Group comparisons by conditions**

**Audio-only**

The percentage of correct responses for BMs was 39.42% (median = 40.0, SD = 15.39, 95% CI: [35.14, 43.71]), for NBMs it was 29.37% (median = 30, SD = 12.43, 95% CI: [24.89, 33.86]), and for NMs it was 35.71% (median = 30, SD = 14.69, 95% CI: [29.03, 42.40]). A Kruskal-Wallis test revealed significant differences in the percentage of correct responses (H(2) = 9.32, p = 0.009, η2 ＝ 0.072, 95% CI for effect size: [0.0018 0.22]; see top plot of Fig.6), and Steel-Dwass tests showed a significant difference between BMs and NBMs (t = 3.03, p = 0.007).

**Fig.6 Group comparison in the audio-only condition.**
The plot on the left represents the results for BMs, the middle for NBMs, and the right for NMs. The error bars denote standard error of percentage of selecting actual winner. Each dot corresponds to an individual participant’s data. The asterisk marks (*) denote significant differences between groups.

**Visual-only**

The percentage of correct responses for BMs was 37.07% (median = 40.0, SD = 18.64, 95 % CI: [32.17 41.97]), for NBMs it was 49.52% (median = 50, SD = 16.27, 95 % CI: [42.12 56.93]), and for NMs it was 43.16% (median = 40, SD = 18.19, 95 % CI: [34.84 52.94]). A Kruskal-Wallis test revealed significant differences in the percentage of correct responses (H(2) = 7.32, p = 0.026, η2 ＝ 0.057, 95% CI for effect size: [0 0.21];see middle plot of Fig.7), and Steel-Dwass tests showed a significant difference between BMs and NBMs (t = 2.58, p = 0.027).

**Fig.7 Group comparison in the visual-only condition.**

The plot on the left represents the results for BMs, the middle for NBMs, and the right for NMs. The error bars denote standard error of percentage of selecting actual winner. Each dot corresponds to an individual participant’s data. The asterisk marks (*) denote significant differences between groups.

**Audiovisual**

The percentage of correct responses for BMs was 36.56% (median = 40.0, SD = 13.02, 95 % CI: [33.22 39.89]), for NBMs it was 31.60% (median = 30, SD = 16.25, 95 % CI: [24.89 38.31]), and for NMs it was 37.86% (median = 45, SD = 21.93, 95 % CI: [25.21 51.71]). A Kruskal-Wallis test revealed no significant differences in the percentage of correct responses (H(2) = 1.92, p = 0.382, η2 ＝ 0.020; see bottom plot of Fig.8).

**Fig.8 Group comparison in the audiovisual condition.**
The plot on the left represents the results for BMs, the middle for NBMs, and the right for NMs. The error bars denote standard error of percentage of selecting actual winner. Each dot corresponds to an individual participant’s data.

**Reviewer #2:** 2-4. Discussion:

* On the whole, the discussion regarding the results obtained is well structured, but I think it would be important to deepen their analysis, by addressing:

- the nature of the audiovisual interactions in the judgement task, e.g. trying to explain the absence of “synergy” between auditory and visual cues (AV condition), whatever the group;

**Author reply:** Thank you for your valuable comments. In the series of the study about sight-over-sound effect, it has been reported that the accuracy rate in AV condition was at chance level. However, there has not been sufficient verification as to why the accuracy does not improve in AV, which should contain more information than VO condition or AO condition. From the results obtained in our paper, it is difficult to provide a concrete explanation, so we have included this issue in the Limitations section and noted the need for future research.

**Author action:** We added the paragraph as follows:

Line 615-625: The third limitation, or an open question for future study, is why the accuracy rates in the AV condition did not surpass those in the AO or VO conditions. Because the AV condition provided more information for evaluators, one might assume that the accuracy rates in the AV condition would be higher than in the AO and VO conditions. However, neither this study nor a series of prior research on the sight-over-sound effect has reported superior accuracy in the AV condition over the other conditions. This suggests that too much information may not be beneficial for evaluators. Given that the judging in actual music competitions is conducted in a format closely resembling the AV condition, it is an open question why the accuracy rates in the AV condition was at chance level. Future studies are needed to investigate why the accuracy rates in the AV condition did not surpass those in the AO or VO conditions.

**Reviewer #2:** - the potential limitations of your study, notably the factors which may have interfered with the results obtained, e.g.: (i) potential interactions between the duration of stimuli and the perceptiveness of auditory and visual cues, (ii) the assessment of only one type of music ensemble, (iii) the fact that around 30% of subjects of the BM group had other musical experiences than brass practice (which may have interfered with the results obtained), and (iv) the high within-group variability of musical experience in the BM and NBM groups, i.e.: ranges of duration of musical training, age of onset of musical training, duration of brass ensemble training, and age of onset brass ensemble training (see: Shenker et al, Brain Struct Funct 2022).

**Author reply:** Thank you for your valuable feedback. Among the points you raised, we particularly consider (ii), (iii) and (iv) to be crucial information for interpreting the results. We have added them as potential limitations of our study in the Discussion section.

**Author action:**

We have added the sentences about (ii) the assessment of only one type of music ensemble. We referenced Chiba et al., 2019, which studied cultural differences in the sight-over-sound effect, to further discuss the need to examine the Sight-over-sound effect across various musical genres.

Line 501-509: While previous studies focused on solo performances of Western-classical music and Japanese shamisen music, this study examined group performances by Japanese brass bands. This finding aligns with the hypothesis posited by Chiba et al. [23], suggesting that the effect of variation in performance level may be genre- and culture-dependent. Taken together, the interplay between variation in performance level and genre emerges as a crucial factor in interpreting the sight-over-sound effect. Pursuing research on the sight-over-sound effect across diverse musical contexts, as Chiba et al. [23] suggested, will help identify how variation in performance level and genre influences performance evaluations.

We have added the sentences about (iii) the fact that around 30% of subjects of the BM group had other musical experiences than brass practice (which may have interfered with the results obtained).

Line 557-566: In this study, we tested a total of 171 BMs, and 118 of them had not only brass band experience but also experience in other musical genres. This raises the question of whether the presence or absence of experience in other musical genres affect the results in BMs. Therefore, we subdivided the BMs into participants with both brass band and other musical experiences (n = 118) and those with only brass band experience (n = 53), and compared the accuracy rates between the two subgroups. The results showed no statistically significant differences between the subgroups in any condition (Kruskal-Wallis test: BMs with other musical experience: H(2) = 3.15, p = 0.21, η2 = 0.027; BMs without other musical experience: H(2) = 0.26, p = 0.88, η2 = 0.005). This suggests that the effect of the presence or absence of experience in other musical genres may be minor in BMs.

We have added subsection about Limitations in the last of Discussion sections and provided following sentences about (iv) the high within-group variability of musical experience in the BM and NBM groups.

Line 608-614: The second limitation of this study is the variability of the age at which musical training began among BMs and NBMs. There was considerable variation in the onset age of musical training among participants in this study. Previous studies have reported structural and functional plastic changes in the brain due to music training in early childhood [44,45]. While our study did not consider the onset age or duration of music training, investigating the relationship between such musical backgrounds and the Sight-over-sound effect may lead to a deeper discussion on the impacts of musical experience.

**Reviewer #2:** * I also have some questions about and suggestion for the 4 explanatory factors you mentioned:

- Variation in performance level and genre (page 18, lines 351-354): Regarding the findings of Chiba and colleagues, couldn't these disparities also be linked to intercultural differences in movements induced by musical type, between Western classical music and Japanese shamisen music?

**Author reply:** Yes, the observed disparities could be linked to intercultural differences in movements induced by musical genres, specifically between Western classical music and Japanese shamisen music. We believe that the influence of the Sight-over-sound effect through cross-cultural comparisons, as suggested by Chiba and colleagues, offers a relevant perspective to explain our findings.

**Author action:**

We added the following sentence.

Line 490-493: Chiba et al. suggest that such differences in the dominance of auditory and visual cues may be observed due to movements induced by music genres from different cultures, such as Western classical music and Japanese shamisen music.

**Reviewer #2:** - Difference between solo and group performances (page 19, lines 370-390): Additionally, you could also mention the type of performed movements, more static (spatially speaking) for brass bands than for other types of ensembles (string orchestra, for example).

**Author reply:** Thank you for your valuable comment. Indeed, the magnitude of movement may be associated with the Sight-over-sound effect. Considering that the relative size of the performers appearing in the video stimuli may become smaller in ensemble performances compared to solo performances, we have added a note on the importance of paying attention to the relative size of the performers in the video when examining the Sight-over-sound effect.

**Author action:** We have added following sentences.

Line 523-535: Including our current study, the series of research on the sight-over-sound effect utilized recordings of actual competition performances. It is important to consider that these recordings were dependent on the filming environment, which could result in varying relative sizes of the performers’ movements in solo versus group performances. Specifically, the movements of group performers could appear relatively smaller compared to those of solo performers because of the degree of camera zoom used to capture the solo and group performances. In solo performances, the relative size becomes larger, allowing for the observation of subtle visual changes such as facial expressions. Conversely, in group performances, it may be possible to discern the movements of the entire body and the relationships of movements between performers rather than the detailed movements of each individual. Future research should consider using videos that take into account the actual distance between performers and evaluators, or constructing experimental environments similar to those in actual concert halls, to further investigate the sight-over-sound effect.

**Reviewer #2:** - Evaluator’s musical experience:

. page 20, lines 395-413: This subpart (for the test results) should be placed in the Results part.

**Author reply:** We moved the results of the 1-sample Wilcoxon test from the Discussion section to the Results section.

**Author action:** We added following sentences in Results section.

**Judgement experiment**

**ALLs**

Line 321-326: The results of the 1-sample Wilcoxon signed-rank test showed no significant difference between the mean percentage of correct responses and chance level in both the AO and AV conditions (AO: V = 3405, p = 0.046, r = 0.40; AV: V = 2977, p = 0.079, r = 0.18, respectively). However, a significant difference was observed between the accuracy rate and chance level in the VO condition (V = 3468, p < 0.001, r = 0.40).

**BMs**

Line 342-346: The results of the 1-sample Wilcoxon signed-rank test showed no significant difference between the mean percentage of correct responses and chance level in both the VO and AV conditions (VO: V = 1053, p = 0.13, r = 0.20; AV: V = 1265, p = 0.021, r = 0.30, respectively). However, a significant difference was observed between the accuracy rate and chance level in the AO condition (V = 1033, p = 0.002, r = 0.44).

**NBMs**

Line 366-371: The results of the 1-sample Wilcoxon signed-rank test showed no significant difference between the mean percentage of correct responses and chance level in both the AO and AV conditions (AO: V = 190, p = 0.17, r = 0.24; AV: V = 144, p = 0.63, r = 0.10, respectively). However, a significant difference was observed between the accuracy rate and chance level in the VO condition (V = 215, p < 0.001, r = 0.76).

**NMs**

Line 386-389: The 1-sample Wilcoxon signed-rank test indicated that none of the percentages exceeded the chance level (AO: V = 141, p = 0.38, r = 0.19; VO: V = 137, p = 0.03, r = 0.53; AV: V = 59, p = 0.36, r = 0.25).

**Reviewer #2:** . page 20, lines 413-414: These findings could be put into perspective with possible differences in the intergroup comparisons depending on the experimental conditions AO, VO and AV (see above).

**Author reply:** As responded above, we have added the results of intergroup comparisons by conditions in the revised manuscript.

**Author action:** We have added following sentences in the Evaluator’s musical experience.

Line 548-556: In this study, the sight-over-sound effect was not confirmed in BMs, whereas it was confirmed in NBMs. When comparing the average accuracy rates for each experimental condition across participant groups, it was found that in the AO condition, the accuracy rate of BMs was significantly higher than that of NBMs (see Fig 6). Conversely, in the VO condition, the accuracy rate of NBMs was significantly higher than that of BMs (see Fig.7). These results suggest that when evaluating excerpts from the brass band competition used in this study, BMs are able to judge more accurately from auditory-only information while NBMs are able to judge more accurately from visual-only information in their judgments.

**Reviewer #2:** . page 21, 425-427: Perhaps you could introduce in this subpart the notion of mirror neuron system, with their differential activation depending on the degree of familiarity with the observed action, musical practice and level of expertise? (e.g.: Baumann et al, Brain Res 2007; Calvo-Merino et al, Cereb Cortex 2005; Petrini et al, Neuroimage 2011).

**Author reply:** Thank you for your comment and for providing informative references. We have revised our manuscript to include a discussion on a series of prior studies concerning the mirror neuron system, which may explain why the sight-over-sound effect is influenced by musical experience. We have also addressed the auditory-motor interaction and its connection to musical practice and level of expertise.

**Author action:** The following sentences have been incorporated into the discussion section.

Line 567-578: Under the auditory-only condition, why were BMs more accurate than NBMs and NMs? We propose that BMs’ extensive experience playing in brass bands could enhance their ability to detect nuanced differences in the sounds. This enhanced auditory discernment might stem from the development of auditory-motor connections and the mirror neuron system, which could be both fortified through rigorous brass band practice. Mirror neurons react to one's own actions and the observation of others' actions [35,36], as well as related sounds [37–39]. Previous studies showed these auditory-motor interactions in musicians [40–43], and heightens conscious motor imagery in response to sounds [29]. Consequently, we hypothesize that BMs can anticipate a performer's movements based on the sounds they perceive, enabling them to discern fine nuanced sound variations. This skill is particularly pronounced in AO condition, potentially leading to the observed higher accuracy rates in BMs.

References

[29] Zatorre RJ, Chen JL, Penhune VB. When the brain plays music: auditory-motor interactions in music perception and production. Nat Rev Neurosci. 2007;8: 547–558. doi:10.1038/nrn2152

[35] Rizzolatti G, Fogassi L, Gallese V. Neurophysiological mechanisms underlying the understanding and imitation of action. Nat Rev Neurosci. 2001;2: 661–670. doi:10.1038/35090060

[36] Calvo-Merino B, Glaser DE, Grèzes J, Passingham RE, Haggard P. Action observation and acquired motor skills: an FMRI study with expert dancers. Cereb Cortex. 2005;15: 1243–1249. doi:10.1093/cercor/bhi007

[37] Kohler E, Keysers C, Umiltà MA, Fogassi L, Gallese V, Rizzolatti G. Hearing sounds, understanding actions: action representation in mirror neurons. Science. 2002;297: 846–848. doi:10.1126/science.1070311

[38] Keysers C, Kohler E, Umiltà MA, Nanetti L, Fogassi L, Gallese V. Audiovisual mirror neurons and action recognition. Exp Brain Res. 2003;153: 628–636. doi:10.1007/s00221-003-1603-5

[39] Lahav A, Saltzman E, Schlaug G. Action representation of sound: audiomotor recognition network while listening to newly acquired actions. J Neurosci. 2007;27: 308–314. doi:10.1523/JNEUROSCI.4822-06.2007

[40] Baumann S, Koeneke S, Meyer M, Lutz K, Jäncke L. A network for sensory-motor integration: what happens in the auditory cortex during piano playing without acoustic feedback? Ann N Y Acad Sci. 2005;1060: 186–188. doi:10.1196/annals.1360.038

[41] Bangert M, Peschel T, Schlaug G, Rotte M, Drescher D, Hinrichs H, et al. Shared networks for auditory and motor processing in professional pianists: evidence from fMRI conjunction. Neuroimage. 2006;30: 917–926. doi:10.1016/j.neuroimage.2005.10.044

[42] Petrini K, Pollick FE, Dahl S, McAleer P, McKay LS, Rocchesso D, et al. Action expertise reduces brain activity for audiovisual matching actions: an fMRI study with expert drummers. Neuroimage. 2011;56: 1480–1492. doi:10.1016/j.neuroimage.2011.03.009

[43] Rosenkranz K, Williamon A, Rothwell JC. Motorcortical excitability and synaptic plasticity is enhanced in professional musicians. J Neurosci. 2007;27: 5200–5206. doi:10.1523/JNEUROSCI.0836-07.2007

**Reviewer #2:** Additionally, could you also discus an alternative hypothesis that auditory cues based judgement for brass music may be more accurate for BMs than for NBMs?

**Author reply:** As responded previously, we observed that the BM group exhibited a higher percentage of correct responses in the AO condition compared to the NBM group. This observation has been discussed in the revised manuscript.

**Author action:** Please see the following discussion section in the revised manuscript.

Line 567-578: Under the auditory-only condition, why were BMs more accurate than NBMs and NMs? We propose that BMs’ extensive experience playing in brass bands could enhance their ability to detect nuanced differences in the sounds. This enhanced auditory discernment might stem from the development of auditory-motor connections and the mirror neuron system, which could be both fortified through rigorous brass band practice. Mirror neurons react to one's own actions and the observation of others' actions [35,36], as well as related sounds [37–39]. Previous studies showed these auditory-motor interactions in musicians [40–43], and heightens conscious motor imagery in response to sounds [29]. Consequently, we hypothesize that BMs can anticipate a performer's movements based on the sounds they perceive, enabling them to discern fine nuanced sound variations. This skill is particularly pronounced in AO condition, potentially leading to the observed higher accuracy rates in BMs.

**Reviewer #2:** Conclusion:

The conclusion should open on some perspectives for future research (e.g., additional studies, …).

**Author reply:** Following your suggestions, we have incorporated a section detalling the limitations of this study and perspectives for future research before the conclusion section.

**Author action:**

Line 606-607: Taken together, it is important to test if the results in this study can be replicated using within-subjects designs in future studies.

Line 612-614: While our study did not consider the onset age or duration of music training, investigating the relationship between such musical backgrounds and the Sight-over-sound effect may lead to a deeper discussion on the impacts of musical experience.

Line 621-625: Given that the judging in actual music competitions is conducted in a format closely resembling the AV condition, it is an open question why the accuracy rates in the AV condition was at chance level. Future studies are needed to investigate why the accuracy rates in the AV condition did not surpass those in the AO or VO conditions.

**[Minor revisions]**

**Reviewer #2:** 3-1. Tittle page:

* Main tittle (lines 1-2): I’d suggest a more "committed" title, e.g.: “Partial replicability”, “Limited replicability” or “Non-replicability of…”.

**Author reply:** In light of your feedback, we have revised the title to incorporate more precise and committed wording.

**Author action:** We have changed main title as follows.

Revised main title: Sight-over-sound effect depends on evaluators’ musical experience: An examination using Japanese brass band competition recordings

**Reviewer #2:** * Running tittle (lines 5-6): Please consider a more concise running title, for example by deleting “Replicability of the”.

**Author reply:** We have changed the running title to be more concise**.**

**Author action:** We have changed running title as follows.

Revised running title: Sight-over-sound effect in brass band competitions.

**Reviewer #2:** * Author Contributions (lines 17-20): Please, avoid initial capital letters for common nouns.

**Author reply:** Thank you.

**Author action:** We have revised as follows.

Revised author contribution: TS and SF conducted conceptualization, methodology, validation and visualization. TS conducted data curation, formal analysis, investigation, software programming. SF conducted supervision. TS and KH conducted writing - original draft preparation, and TS and SF conducted writing – review and editing preparation.

**Reviewer #2:** * Keywords (PDF document):

If they will be used for the referencing of the paper, I’d suggest adding “audiovisual interactions” and/or “multisensory integration”.

**Author reply:** Thank you for your suggestion. We agreed.

**Author action:** We added both "audiovisual interactions" and "multisensory integration" as keywords.

**Reviewer #2:** 3-2. Introduction:

* Page 3, line 44: please, add “conditions” at the end of the line (“between the two conditions”).

**Author reply:** We added “conditions” at the end of the line.

**Author action:** Please see the Lines 46-47:

Line 46-47: …reporting that the evaluations of the performances differed significantly between the two conditions, …

**Reviewer #2:** * Page 3, line 60: please, use lowercase letter for “the effect” (“different stimuli: the effect was”).

**Author reply:** The sentence you mentioned was removed from the revised manuscript. Additionally, we identified and corrected a similar error.

**Author action:** Please see the corrected sentence below:

Line 43-44: … visual information in music performance [2]: visual information plays a significant role in appreciating performer expressiveness …

**Reviewer #2:** * Pages 4-5, lines 66-95: I’d suggest you list the 3 issues in a single paragraph (without line breaks).

**Author reply:** We agreed.

**Author action:** We removed paragraph breaks and combined them into a single paragraph.

**Reviewer #2:** * Page 5, line 89: I’d suggest to use “professional musicians” instead of “individuals with musical experience”.

**Author reply:** Thank you for your comment. However, as we have removed the discussion on audio-visual perception from the relevant section, we have also deleted the sentences related to this comment.

**Reviewer #2:** * Page 5, line 103: I’d suggest to add “in controlled conditions” at the end of the sentence.

**Author reply:** We agreed.

**Author action:** We added “in controlled conditions” at the end of the sentence on line 103.

Line 127-128: replicability of the sight-over-sound effect in the judgment of Japanese brass band competitions in controlled conditions.

**Reviewer #2:** 3-3. Methods:

* Ethics Statement (page 7, line 123): I’d suggest to add “no possibility” (“thereby ensuring no possibility of association”).

**Author reply:** We agreed.

**Author action:** We added “no possibility ” on line 170.

Line 170: … thereby ensuring no possibility of association with their personal information.

**Reviewer #2:** * Participants:

- Pages 7-8, first paragraph: For this subpart, you should avoid redundant information between text and Table 1 (e.g., lines 139-143).

**Author reply:** We have removed information from the main text that was present in Table 1.

**Author action:** We have removed the redundant information and revised the participant section as follows.

Line 180-206: A total of 301 adults participated in this study (70 male participants and 231 female participants, mean age = 34.81 years, standard deviation [SD] = 11.71, range = 18−56 years). The participants were asked if they had any musical training experience, except for mandatory music education in schools or other educational institutions. If they had musical training experience, we asked them if they had specific experience of playing in brass bands. We also asked the participants about the musical instruments they had practiced, the age of commencement, and the duration of musical training. Based on their musical backgrounds, they were subdivided into three groups: brass band musicians (BMs), non-brass band musicians (NBMs), and non-musicians (NMs; see Table 1). Both BMs and NBMs had musical training experience; the former had specific experience of playing in brass bands, but the latter did not. The NMs were participants who did not have any musical experience, except for mandatory music education in schools or other educational institutions. The number of participants in each group was as follows: BMs: 171 (46 male participants and 125 female participants~~, mean age = 33.08 years, SD = 11.57, range = 18−56 years~~); NBMs: 78 (16 male participants, 62 female participants~~; mean age = 35.39 years, SD = 13.03, range = 18−58 years~~); and NMs: 52 (8 male participants and 44 female participants~~, mean age = 39.15 years, SD = 8.73, range = 19−55 years;~~ see Table 1 for participant’s gender, mean age, standard deviation of age and age range).

BMs had musical training experience in brass bands (mean = 8.07 years, SD = 5.37, range = 1−35 years, see Table 1 for BMs mean age of commencement of brass band training). A total of 137 BMs participated in brass band competitions, and 12 of them had participated in the highest competition levels (e.g., All Japan Brass Band Competition). Eighteen BMs reported having experience as brass band clinicians.

Among BMs, 118 participants had musical training other than brass bands training (mean = 10.985 years, SD = 8.749, range = 1−48 years, vocal: 6, keyboards: 96, strings: 3, wind instruments: 26, percussion: 2). The mean duration of musical training in NBMs was 11.72 years (SD = 7.81, range = 1−34 years, vocal: 15, keyboards: 39, strings: 14, wind instruments: 3, percussion: 3, Japanese traditional instruments: 4; see Table 1 for NBMs mean age of commencement of musical training).

**Reviewer #2:** - Page 8-lines 144-146: This sentence does not seem to me to be understandable; I’d suggest to use “other than” instead of “excerpt”.

**Author reply:** We did.

**Author action:** We changed to use "other than" instead of "except".

Line 201: … participants had musical training other than brass bands training …

**Reviewer #2:** - Page 8, lines 154-160: I’d suggest to move this paragraph before the previous one (starting at line 144).

**Author reply:** We agreed.

**Author action:** We moved this paragraph before the previous one.

**Reviewer #2:** * Stimuli (page 9, lines 164-166): I’d suggest to rephrase this sentence by using a verb.

**Author reply:** We agreed.

**Author action:** We rephrased this sentence by using a verb.

Line 212-213: We utilized the brass band performance recordings of 30 high school brass bands participating in Japanese regional brass band competitions (final qualifying rounds).

**Reviewer #2:** * Procedure (page 11, lines 202-204): It would have been interesting to propose the same type of questions after the judgment tasks, in order to carry out a pre-post-test comparison and to look for possible correlations with the experimental results.

**Author reply:** Thank you for your feedback. Indeed, comparing responses about the importance of audiovisual information before and after the experiment would have been valuable. However, since we did not conduct a survey after the evaluation experiment in this study, we are unable to perform a before-and-after comparison, but there might be changes in individuals' subjective impressions.

**Reviewer #2:** * Statistical analysis (page 12, lines 226): In this case, “To assess” would be more appropriate than “To confirm”.

**Author reply:** Corrected.

**Author action:** We changed to use “To assess” instead of “To confirm”.

Line 291: To assess the normality of the data, …

**Reviewer #2:** 3-4. Results:

* Judgment experiment:

- For ALLs (page 13, lines 242-245): You should presented the results in the same order as for Figure 2.

**Author reply:** Corrected.

**Author action:** We changed to present results in the same order as Figure 2.

Line 317-320: For ALLs, the mean percentage of correct responses was 35.62 % in the AO condition (median = 40.0, SD = 14.93, 95 % CI: [32.98 38.81]), 40.92 % in the VO condition (median = 40.0, SD = 18.56, 95 % CI: [36.94 44.78]), 35.62 % in the AO condition (median = 40.0, SD = 14.93, 95 % CI: [32.98 38.81]) and 35.50 % in the AV condition (median = 40.0, SD = 15.20, 95 % CI: [32.30 38.78]; see Fig 2.).

**Reviewer #2:** - For NBMs (page 14, lines 267-270): You should presented the results in the same order as for Figure 4.

**Author reply:** Corrected.

**Author action:** We changed to present results in the same order as Figure 4.

Line 358-361: For the NBM group, the percentage of correct responses was 29.37 % in the AO condition (median = 30, SD = 12.43, 95 % CI: [24.89 33.86]), 49.52 % in the VO condition (median = 50, SD = 16.27, 95 % CI: [42.12 56.93]) , 29.37 % in the AO condition (median = 30, SD = 12.43, 95 % CI: [24.89 33.86]) and 31.60 % in the AV condition (median = 30, SD = 16.25, 95 % CI: [24.89 38.31]; see Fig 4.).

**Reviewer #2:** - For NMs (pages 14-15, lines 284-287): You should presented the results in the same order as for Figure 5.

**Author reply:** Corrected.

**Author action:** We changed to present results in the same order as Figure 5.

Line 382-385: For the NM group, the percentage of correct responses was 35.71 % in the AO condition (median = 30, SD = 14.69, 95 % CI: [29.03 42.40]), 43.16 % in the VO condition (median = 40, SD = 18.19, 95 % CI: [34.84 52.94]), 35.71 % in the AO condition (median = 30, SD = 14.69, 95 % CI: [29.03 42.40]) and 37.86 % in the AV condition (median = 45, SD = 21.93, 95 % CI: [25.21 51.71]; see Fig 5.).

**Reviewer #2:** 3-5. Discussion:

* Page 16, lines 296-297: I’d suggest to add “in controlled conditions” at the end of this introductory sentence.

**Author reply:** We did.

**Author action:** We added “in controlled conditions” at the end of this sentence.

Line 443-444: The purpose of this study was to investigate the replicability of the sight-over-sound effect in the judgment of Japanese brass band competitions in controlled conditions.

**Reviewer #2:** * Page 16, lines 297-298: Perhaps you should use “main” or primary”, rather than “initial” hypothesis.

**Author reply:** Corrected.

**Author action:** We used “primary hypothesis” instead of “initial hypothesis”.

Line 444-445: Our primary hypothesis (H1) stipulated that …

**Reviewer #2:** * Page 18, lines 346-347: Only one quote should be sufficient for this sentence.

**Author reply:** Corrected.

**Author action:** Removed a quote at the end of this sentence.

Line 482-483: Mehr et al. ~~[18]~~ highlighted that the degree of variation in performance level is a significant factor influencing the sight-over-sound effect [22].

**Reviewer #2:** * Page 21, lines 435-438: I’d suggest to add “auditory” (“or to precisely identify crucial auditory or visual cues…”).

**Author reply:** Thank you for your comment. The sentence has been removed in the revised manuscript.

**Reviewer #2:** 3-6 Ending section:

* Data Availability (page 23, lines 463-465): If possible, I think it would be relevant to provide a set of the experimental stimuli used (in the three conditions: AO, VO and AV), in the supporting information files, in order to help the reader to better figure out the protocol.

**Author reply:** We were unable to include the video as part of the supporting information files because it contains identifiable information, raising the possibility that individual could be recognized from the footage.

**Reviewer #2:** 3-7. References:

* Page 25, lines 515-516: The Reference 17 should be completed (type, university, web link).

**Author reply:** We did.

**Author action:** We added information about Reference 21.

[21] Scannell DA. Sound Judgment : Auditory – but not Visual – Information Reveals Musical Competition Winners. Boston College, 2014. pp. 1–27. http://hdl.handle.net/2345/3867

**Reviewer #2:** 4. Figures:

4-1. Figure 1:

* Caption (page 6, lines 186-195): To be clearer, could you briefly explain (either here or in the Stimuli part) the criteria for choosing the experimental sets?

**Author reply:** In accordance with our response to another of your comment, the criteria for selection involved creating a set of three bands. This included one gold award winner advancing to the national competition and two gold award winners not advancing, with all three bands performing the same designated piece.

**Author action:** For details about the criteria used to select the experimental sets, please refer to the S3 text S2 Fig in Supplementary Information.

**Reviewer #2:** 5. Supplementary information

For the S1 Figure caption (lines 25-26), please consider the alternative proposition: “These graphs respectively represent…”

**Author reply:** Thank you for your feedback. We have made the revisions as per your suggestions.

**Author action:**

Revised Caption: These graphs respectively represent comparisons of the selection rates of the importance of audio and visual information in the judging brass band competitions among BMs (top), NBMs (middle), and NMs (bottom).

**Reviewer #3:** Here, the authors explored the classic “sight over sound” effect using a large sample with a well-controlled and novel stimulus set. They find that the effect is not reliably replicated and suggest that this is likely due to using such well-controlled stimuli. They also demonstrate that the effect may be somewhat dependent on musical experience.

Overall, the manuscript is very well written and thoughtful. However, there are a few sections where the authors should add clarity as I found them difficult to understand: (1) The methods, (2) stimuli, and (3) participant sections.

**Author reply:** We sincerely appreciate your valuable comments. Below, we have provided responses to each of the questions.

**Reviewer #3:** For example, while the authors provided a graphic, it is not immediately apparent how many trials participants do, the number of pieces that are possibly assigned (eg, do all participants see all pieces of music?), whether the correct answer is always the same piece of music (it appears that the winners always performed the piece “March..”, etc.

**Author reply:** Thank you for your constructive comment. As you pointed out, we have made revisions to clarify the experimental procedures and the selection method for the experimental stimuli.

**Author action:**

We have described the criteria for selecting the stimuli in the main text as follows.

Line 212-228: We utilized the performance recordings of 30 high school brass bands participating in Japanese regional brass band competitions (final qualifying round) as experimental stimuli. The recordings were organized in a three-choice format, each consisting of the same song performed by three different bands. This resulted in a total of 10 distinct sets, each made up of three groups (see S2 text and Fig S2. for detailed information on the composition of all sets, the competitions of each group's stimuli, and the pieces performed). One of the three brass bands in a set qualified for the All Japan Brass Band Competition. The other two brass bands were placed highly in regional competitions, but did not qualify for national competitions. Mehr et al., (Mehr et al. 2018) reported that the sight-over-sound effect was not observed when comparing stimuli with significant differences in performance skills. Therefore, to minimize the disparity in performance skills, we used groups that received gold awards (about top 30% in the competition) as stimuli rather than silver or bronze award recipients. The musical pieces used as stimuli were selected from past brass band competition assigned pieces from 2012 to 2018. Each year, the brass band association selects five assigned pieces, and all participating brass bands must choose and perform one of these five pieces. Leveraging this characteristic, the three stimuli in a single stimulus set were made to be the same piece performed by three different groups.

Also, we have added the following text to clarify the experimental procedures, making the process more transparent.

Line 258-261: In the judging experiment, participants were asked to select the performance they felt would achieve the best results in a competition from three stimuli in each set (see Figure 1). Participants worked on 10 judgement tasks for the brass band performance assigned experimental condition.

To further clarify the experimental procedures, we have created a new figure (Fig.1).

**Figure 1. Overview the procedure of the Judgement experiment.**
Example of procedure in the judgement experiment. Each participant performs a three-choice performance judgement task ten times. The order of questions and the playback sequence of the three stimuli in each set are both randomized. Participants are asked to listen to three consecutive performance recordings from a single set and then choose the one they believe achieved the highest rating in the competition. The experiment is conducted with a between-subjects design, and each participant only participates in one of the three experimental conditions assigned. The stimulus images in the procedures for the Visual-only and Audiovisual conditions are not actual experimental stimuli, but reference images created by the authors.

**Reviewer #3:** On Page 8, lines 144-148, what is this sentence referring to, given the context?

**Author reply:** We have revised our manuscript to clarify the sentence. We examined whether the presence or absence of musical experience other than brass band within the BM group affects the sight-over-sound effect.

**Author action:** Please see the following paragraph in the revised manuscript.

Line 557-566: In this study, we tested a total of 171 BMs, and 118 of them had not only brass band experience but also experience in other musical genres. This raises the question of whether the presence or absence of experience in other musical genres affect the results in BMs. Therefore, we subdivided the BMs into participants with both brass band and other musical experiences (n = 118) and those with only brass band experience (n = 53), and compared the accuracy rates between the two subgroups. The results showed no statistically significant differences between the subgroups in any condition (Kruskal-Wallis test: BMs with other musical experience: H(2) = 3.15, p = 0.21, η2 = 0.027; BMs without other musical experience: H(2) = 0.26, p = 0.88, η2 = 0.005). This suggests that the effect of the presence or absence of experience in other musical genres may be minor in BMs.

**Reviewer #3:** What is 55 referring to, and why do the numbers not seem to sum properly?

**Author reply:** Thank you for bringing this to our attention. Another reviewer also noted this issue. Upon re-examining our data, we confirmed that the number of BMs with musical experience other than brass band experience was 118, not 55.

**Author action:** The following sentence has been revised accordingly:

Line 201-203: Among BMs, 118 participants had musical training other than brass bands training (mean = 10.98 years, SD = 8.79, range = 1−48 years, vocal: 6, keyboards: 96, strings: 3, wind instruments: 26, percussion: 2).

**Reviewer #3:** Therefore, and perhaps I’m misunderstanding, but I’m unsure how participants could select a winner when there appears to be a winner for only one musical piece. Were participants always presented with this piece on each trial? Or are they simply supposed to pick whichever performance got the highest rating given the three they are comparing them to?

**Author reply:** Thank you for your feedback. We acknowledge that the Methods section was not sufficiently clear. In our study, participants viewed three videos for each question and chose the performance they rated the highest. Each question featured three videos with different groups performing the same musical piece. Additionally, the musical piece varied with each question to ensure that the same piece was not repeated across all questions.

**Author action:** We have revised the methods and have updated Figure 1 to more clearly depict the experimental procedure.

**Reviewer #3:** Since the study is conducted between-subjects, and participants appear to often be at chance levels of performance, it seems plausible that one condition is better than another simply because those participants are better at any given task and not just the particular condition they are in. Furthermore, could the lack of an effect be driven by floor performance? In other words, could the presence of an effect for NBMs be due to that group of participants being better, overall, at any task (since it is my understanding that the VO group for NBMs are a separate group of people than those in the AO condition)? This is why an orthogonal task, to detect baseline levels of performance of a particular group is important. Did the authors include a separate task to get at this potential mechanism?

**Author reply:** Thank you for your important comment. As you pointed out, since this study was conducted using only a between-subjects design, we cannot deny the possibility that participants who perform well under any condition might have been inadvertently gathered. Additionally, because this study was conducted online, the experimental environment varied among participants, which could have influenced the results. We have noted these two points as limitations of this study.

**Author action:**

We added the following sentences in the Discussion section.

Line 598-607: First, we did not employ a within-subjects design but a between-subjects design. We employed the between-subjects design to minimize learning and transfer effects that might arise from participants engaging with multiple experimental conditions. However, using a between-subjects design entails the possibility that participants who could achieve high accuracy rates in any condition were coincidentally assigned to the conditions with higher accuracy rates. Additionally, as the experiment was conducted online, the experimental conditions could have varied for each participant, and those who had better experimental condition might have been coincidentally assigned to different conditions. Taken together, it is important to test if the results in this study can be replicated using within-subjects designs in future studies.

**Reviewer #3:** Similar to combining all participants into an ALL group, could the authors look at each stimulus and see its average accuracy dependent on the modality (AO, VO, and AV). This could tell us whether the stimuli were driving some of the observed differences in accuracy (eg, between BM and NBM, for AO and VO respectively) and whether the stimuli are somewhat responsible for the sight over sound effect for NBMs. In short, could it be that the stimuli are driving the effect for NBMs instead of the purported differences in mental imagery described in the discussion?

**Author reply:** We are grateful for your insightful comment. We conducted additional analyses comparing participant groups under matched audiovisual conditions. These additional analyses revealed that in the AO condition, BMs were significantly more accurate than NBMs in predicting the winner. Conversely, in the VO condition, NBMs were significantly more accurate than BMs. We have included these findings from the additional analysis in the Results section.

**Author action:**

The results of the intergroup comparisons, as per the conditions, have been incorporated into the manuscript as follows:

Line 396-440:

**Group comparisons by conditions**

**Audio-only**

The percentage of correct responses for BMs was 39.42% (median = 40.0, SD = 15.39, 95% CI: [35.14, 43.71]), for NBMs it was 29.37% (median = 30, SD = 12.43, 95% CI: [24.89, 33.86]), and for NMs it was 35.71% (median = 30, SD = 14.69, 95% CI: [29.03, 42.40]). A Kruskal-Wallis test revealed significant differences in the percentage of correct responses (H(2) = 9.32, p = 0.009, η2 ＝ 0.072, 95% CI for effect size: [0.0018 0.22]; see top plot of Fig.6), and Steel-Dwass tests showed a significant difference between BMs and NBMs (t = 3.03, p = 0.007).

**Fig.6 Group comparison in the audio-only condition.**
The plot on the left represents the results for BMs, the middle for NBMs, and the right for NMs. The error bars denote standard error of percentage of selecting actual winner. Each dot corresponds to an individual participant’s data. The asterisk marks (*) denote significant differences between groups.

**Visual-only**

The percentage of correct responses for BMs was 37.07% (median = 40.0, SD = 18.64, 95 % CI: [32.17 41.97]), for NBMs it was 49.52% (median = 50, SD = 16.27, 95 % CI: [42.12 56.93]), and for NMs it was 43.16% (median = 40, SD = 18.19, 95 % CI: [34.84 52.94]). A Kruskal-Wallis test revealed significant differences in the percentage of correct responses (H(2) = 7.32, p = 0.026, η2 ＝ 0.057, 95% CI for effect size: [0 0.21];see middle plot of Fig.7), and Steel-Dwass tests showed a significant difference between BMs and NBMs (t = 2.58, p = 0.027).

**Fig.7 Group comparison in the visual-only condition.**

The plot on the left represents the results for BMs, the middle for NBMs, and the right for NMs. The error bars denote standard error of percentage of selecting actual winner. Each dot corresponds to an individual participant’s data. The asterisk marks (*) denote significant differences between groups.

**Audiovisual**

The percentage of correct responses for BMs was 36.56% (median = 40.0, SD = 13.02, 95 % CI: [33.22 39.89]), for NBMs it was 31.60% (median = 30, SD = 16.25, 95 % CI: [24.89 38.31]), and for NMs it was 37.86% (median = 45, SD = 21.93, 95 % CI: [25.21 51.71]). A Kruskal-Wallis test revealed no significant differences in the percentage of correct responses (H(2) = 1.92, p = 0.382, η2 ＝ 0.020; see bottom plot of Fig.8).

**Fig.8 Group comparison in the audiovisual condition.**
The plot on the left represents the results for BMs, the middle for NBMs, and the right for NMs. The error bars denote standard error of percentage of selecting actual winner. Each dot corresponds to an individual participant’s data.

**Reviewer #3:** Similarly, could the “above chance performance” for both BMs and NBMs in AO and VO respectively be, alternatively, due to the stimuli themselves? And not due to any internal mechanism? All of this is relevant since it seems that participants only had one interaction with each stimulus, which could lead to a lack of appropriate measurement power per stimulus. In other words, it could be the case that if participants saw each sample three times that they would get it wrong on one, but right on the other two occasions, demonstrating an overall ability to detect the correct answer.

**Author reply:** Following our additional analysis, which was conducted in response to the previous question, we consider that the outcomes of this study could be due to the differences in musical experiences among the evaluators. With this in mind, we have made revisions to the structure of the Discussion section.

**Author action:** The following sentences have been added to the Discussion section.

Line 567-578: Under the auditory-only condition, why were BMs more accurate than NBMs and NMs? We propose that BMs’ extensive experience playing in brass bands could enhance their ability to detect nuanced differences in the sounds. This enhanced auditory discernment might stem from the development of auditory-motor connections and the mirror neuron system, which could be both fortified through rigorous brass band practice. Mirror neurons react to one's own actions and the observation of others' actions [35,36], as well as related sounds [37–39]. Previous studies showed these auditory-motor interactions in musicians [40–43], and heightens conscious motor imagery in response to sounds [29]. Consequently, we hypothesize that BMs can anticipate a performer's movements based on the sounds they perceive, enabling them to discern fine nuanced sound variations. This skill is particularly pronounced in AO condition, potentially leading to the observed higher accuracy rates in BMs.

**Reviewer #3:** Also, on line 402, the authors state that p = .021 is non-significant. Are they using a separate alpha here?

**Author reply:** Regarding the Kruskal−Wallis test, we applied an adjusted *α* level of 0.05. For the 1-sample Wilcoxon test, we used an adjusted *α* level of 0.016. We have made these points clearer in the revised version of the manuscript.

**Author action:** Please see the following part in the methods:

Line 296-302: To compare the percentage of correct responses between conditions, the Kruskal−Wallis test was used (adjusted *α* = 0.05). There are intragroup differences for the number of participants between conditions (see Table 2), but the Kruskal-Wallis test can be utilized even when sample sizes in the compared groups are not equal (Kruskal and Wallis 1952). In addition, the Steel-Dwass test corrected for multiple comparisons was used as a post-hoc test (adjusted *α* = 0.05). To test the difference between the chance level (33.33%) and the average accuracy rate, a 1-sample Wilcoxon test was used (adjusted *α* = 0.016).

**Reviewer #3:** The authors conclude that, while they fail to replicate the effect with their general sample, they are able to replicate it within the NBMs and thus, the effect depends on musicianship. But I’m a bit unsure of whether that conclusion is warranted. Since, are they implicitly suggesting that the previously observed sight-over-sound effects were somehow secretly driven by musicians? And, in particular, musicians who were not explicit experts in the sub-domain of musicianship used as stimuli in a particular experiment that did replicate the sight-over-sound effect? Basically, couldn’t their positive effect just be noise (ie, could this just be a false positive, Type I error)?

**Author reply:** Thank you for your valuable and insightful comments. Considering your feedback, we have conducted additional analysis using a permutation test. As a result, while no differences in accuracy rate among three conditions were observed for ALLs (χ2 = 5.59, p = 0.061, 99 % CI: [0.046 0.087]), BMs (χ2 = 1.47, p = 0.48, 99 % CI: [0.46 0.54] ) and NMs (χ2 = 1.97, p = 0.37, 99 % CI: [0.35 0.43] ), a statistically significant difference was observed in NBMs (χ2 = 16.93, p < 0.001, 99 % CI: [0.00 0.005]). These results are consistent with the findings of the Kruskal-Wallis test. Taking into account the results of the permutation test, we concluded that the outcomes from the Kruskal-Wallis test are reliable.
