# Supplementary material for: Sight-over-sound effect depends on interaction between evaluators’ musical experience and auditory-visual integration: An examination using Japanese brass band competition recordings
Source: PLoS One. 2025 Apr 29;20(4):e0321442. doi: 10.1371/journal.pone.0321442 (PMC12040236; doi:10.1371/journal.pone.0321442)
Supplement: Attachment — Submitted filename: Response to Reviewers_round2_final.docx [file pone.0321442.s004.docx]

**Manuscript ID:** PONE-D-23-19525

**Type:** Research Article

**Title:** Sight-over-sound effect depends on evaluator’s musical experience: An examination using Japanese brass band competition recordings

**Author:** Samma *et al*

**To:** Dr. Thiago P. Fernandes, Academic Editor PLOS ONE

Dear Dr. Fernandes,

**Re: Reply to reviewers on PONE-D-23-19525**

Thank you very much for your review of our submission, manuscript PONE-D-23-19525, entitled “Replicability of the sight-over-sound effect in the judgement of brass band competitions in Japan,” which we submitted to PLoS ONE.

In this letter, we summarize how we addressed the comments made by the reviewers. We found the reviews to be extremely helpful and have done our best to utilize them to strengthen the paper.

Below, we have provided a detailed explanation of how we addressed each of the comments made by the reviewers.

We greatly appreciate your efforts as an editor of the journal and your contribution to improving our manuscript.

Thank you very much.

Tomohiro Samma, Kazuaki Honda, and Shinya Fujii PhD

**Reviewers’ Comments to the Author:**

**Reviewer #1:** I do appreciate the authors’ thoughtful revision and responses to the comments. Introduction is now good to describe the gap in the current literature and provide rationale for the hypotheses. Revisions of other sections also has improved the quality of the manuscript. I have no other comments.

**Author reply:** We appreciate your comments on the previous version of our manuscript. Your valuable feedback was extremely helpful in improving our paper.

**Reviewer #2: 1. General comments:**
In this revised version of the manuscript, the authors have addressed almost all of the issues previously raised.
Notably, they improved the Introduction section, clarified the experimental procedure, reorganized the Results section, and strengthened the Discussion section.
Additionally, they provided intergroup comparisons (along with three additional figures) and discussed some limitations of their study.
Finally, they expanded the Supplementary Information section.
In all, the authors have adequately revised their manuscript which is now publishable in PLoS One, after some minor revisions.

**Author reply:** We sincerely appreciate the specific and constructive comments you provided. Based on your feedback, we have made efforts to further improve our paper. Please see our responses below to each of your comments.

**Author action:** Please see our actions outlined below.

**Reviewer #2:** **2. Minor revisions:**

For page and line numbers, I will refer to the Word document (unless otherwise specified).

**2-1. Abstract:**

- Page 1, lines 29, 31-33: For the indication of age, please, add “+/- SD” (average age +/- SD = …).

- Page 1, lines 31-33: You should delete the three "n= " at the beginning of the three parts in parentheses.

**Author reply:** Please see the corrected sentences highlighted in red below.

**Author action:**

Line 29: A total of 301 adults (average age ± SD = 34.81 ± 11.71 years) participated…

Line 31-33: Among the participants, 171 were brass band musicians (~~n = 171;~~ average age ± SD = 33.08 ± 11.57 years), 78 were non-brass band musicians (~~n = 78;~~ average age ± SD = 35.39 ± 13.03 years), and 52 were non-musicians (~~n = 52;~~ average age ± SD = 39.15 ± 8.73 years).

**Reviewer #2:** **2-2. Methods:**

- Pages 10-11, lines 220-223: The authors' response and Mehr et al's findings suggest that the differences in musical performance skills are basically more related to auditory information than to visual information. The choice of gold prize awarded brass bands only could have partially neutralized the effect of auditory information (almost equivalent levels between groups) in favor of visual information. In this condition, visual information could have become the main cue for achieving the national selection.

**Author reply:** Thank you for your thoughtful comment. We considered your perspective to be very important for interpreting our results, and thus we revised our manuscript to add a limitation in the discussion section regarding differences in performance levels.

**Author action:** Please see the following revised discussion.

Revised discussion (see Pages 28-29, lines 620-636):

“As a third limitation, this study did not test whether the sight-over-sound effect could be observed in cases where there were clear differences in brass band performance levels. The stimuli used in our study were limited to those that received gold prizes at regional brass band competitions, resulting in relatively small differences in performance levels for comparison. This was because a previous study by Mehr et al. [22] reported that the sight-over-sound effect was not observed when comparing stimuli with significant differences in performance skills. Based on the results from this study and Mehr et al.'s findings, it can be assumed that clear differences in musical performance skills are generally more related to auditory information than to visual information. Specifically, it can be assumed that selecting only gold prize-awarded brass bands might have partially neutralized the effect of auditory information (almost equivalent levels between groups) in favor of visual information. In this condition, visual information could have become the main cue for selecting the winner. Additionally, the BMs in this study showed more accurate judgments than the NBMs and NMs because they might have been able to notice the subtle acoustic differences (auditory cues) across the gold-prize brass bands. Thus, further studies are needed to test if the results from this study are replicated under conditions where there are clear differences in brass band performance levels.”

**Reviewer #2:**
- Page 11, lines 241-244: Please clarify whether or not you used the: "Evenly Present Elements" option?

**Author reply:** We have added the clarification to the sentence to indicate that we used the “Evenly Present Elements” option.

**Author action:**

Line 226-227: … the randomization methods with the Evenly Present Elements option implemented in Qualtrics.

**Reviewer #2:**
- Page 13, line 273: Please use “Fig. 1” (instead of Figure 1) and reconsider the title (eg : Overview of the Judgment Experiment Procedure). Furthermore, the first sentence is redundant with the title.

**Author reply:** We used “Fig.1” and changed the title. Additionally, we deleted the first sentence from the caption.

**Author action:**

Line 254-255: Fig~~ure~~ 1. Overview ~~the procedure~~ of the judgement experiment procedure.

~~Example of procedure in the judgement experiment.~~ Each participant performs…

**Reviewer #2: 2-3. Results:**
* Group comparisons by conditions:

- Page 19, lines 398-401: I suggest you rephrase this sentence:

“The percentage of correct responses was: X (median = ...) for BMS, Y (median = ...) for NBMS and Z (median = ...) for NMs.”

**Author reply:** We rephrased the sentence as follows:

**Author action:**

Line 409-411: The percentage of correct responses ~~for BMs~~ was 39.42% (median = 40.0, SD = 15.39, 95% CI: [35.14, 43.71]) for BMs, ~~for NBMs~~ it was 29.37% (median = 30, SD = 12.43, 95% CI: [24.89, 33.86]) for NBMs, and ~~for NMs~~ it was 35.71% (median = 30, SD = 14.69, 95% CI: [29.03, 42.40]) for NMs.

**Reviewer #2:**

- Page 19, lines 413-416: I suggest you rephrase this sentence:

“The percentage of correct responses was: X (median = ...) for BMS, Y (median = ...) for NBMS and Z (median = ...) for NMs.”

**Author reply:** We rephrased the sentence as follows:

**Author action:**

Line 424-426: The percentage of correct responses ~~for BMs~~ was 37.07% (median = 40.0, SD = 18.64, 95 % CI: [32.17 41.97]) for BMs, ~~for NBMs~~ it was 49.52% (median = 50, SD = 16.27, 95 % CI: [42.12 56.93]) for NBMs, and ~~for NMs~~ it was 43.16% (median = 40, SD = 18.19, 95 % CI: [34.84 52.94]) for NMs.

**Reviewer #2:**

- Page 20, lines 430-433: I suggest you rephrase this sentence:

“The percentage of correct responses was: X (median = ...) for BMS, Y (median = ...) for NBMS and Z (median = ...) for NMs.”

**Author reply:** We rephrased the sentence as follows:

**Author action:**

Line 440-442: The percentage of correct responses ~~for BMs~~ was 36.56% (median = 40.0, SD = 13.02, 95 % CI: [33.22 39.89]) for BMs, ~~for NBMs~~ it was 31.60% (median = 30, SD = 16.25, 95 % CI: [24.89 38.31]) for NBMs, and ~~for NMs~~ it was 37.86% (median = 45, SD = 21.93, 95 % CI: [25.21 51.71]) for NMs.

**Reviewer #2: 2-4. Discussion:**

*** Evaluator’s musical experience:**

- Page 25, lines 548-556: An alternative explanation may be related to the choice of gold prize awarded brass bands only (see above, Comment on pages 10-11, lines 220-223): Subtle acoustic differences (auditory cues) across brass bands may have been noticeable only to the BMs.

**Author reply:** Thank you for your valuable suggestion. In response to your comments, we revised the discussion to mention that the results may be related to the choice of gold prize-awarded brass bands only. Specifically, we added a discussion noting that the BMs in this study showed more accurate judgments than the NBMs and NMs because they might have been able to notice the subtle acoustic differences (auditory cues) across the gold-prize brass bands.

**Author action:** Please see the following revised discussion:

Revised discussion (see Pages 28-29, lines 620-636):

“As a third limitation, this study did not test whether the sight-over-sound effect could be observed in cases where there were clear differences in brass band performance levels. The stimuli used in our study were limited to those that received gold prizes at regional brass band competitions, resulting in relatively small differences in performance levels for comparison. This was because a previous study by Mehr et al. [22] reported that the sight-over-sound effect was not observed when comparing stimuli with significant differences in performance skills. Based on the results from this study and Mehr et al.'s findings, it can be assumed that clear differences in musical performance skills are generally more related to auditory information than to visual information. Specifically, it can be assumed that selecting only gold prize-awarded brass bands might have partially neutralized the effect of auditory information (almost equivalent levels between groups) in favor of visual information. In this condition, visual information could have become the main cue for selecting the winner. Additionally, the BMs in this study showed more accurate judgments than the NBMs and NMs because they might have been able to notice the subtle acoustic differences (auditory cues) across the gold-prize brass bands. Thus, further studies are needed to test if the results from this study are replicated under conditions where there are clear differences in brass band performance levels.”

**Reviewer #2:**

- Page 25, lines 557-566: The results of these additional analyzes should be moved to the Results section, in order to only mention the absence of inter-subgroup differences in the Discussion section.

**Author reply:** Considering your comments, we performed a two-way ANOVA to examine the effects of musical experience outside of the brass band. The results from additional analyses have been included in the Results section in the revised manuscript. We decided to delete the results testing the differences among AV, A, and V conditions for each of the BMs with and without other musical experiences because the ANOVA results showed no main effect of musical experience outside of the brass band.

**Author action:** Please see the following Results and Discussion sections.

**Author action:** We added following sentences.

Page 19. Line 399-405 in the Results section:

To examine the main effect of musical experience outside of brass bands in BMs, 2 (presence or absence of musical experience outside of brass bands) × 3 (experimental conditions) two-way analysis of variance was conducted. The results indicated that there was no main effect of musical experience (F (1, 0) = 0.0, p = 0.99, ηp2 < 0.0001), nor was there an interaction between gender and experimental conditions (F (2, 714) = 1.42, p = 0.24, ηp2 = 0.02). Therefore, it is considered unlikely that the music experience outside of brass bands influenced the results of the current experiment.

Page 26, Lines 567-569, Discussion section:

Nevertheless, we could not find main effect of musical experience outside of brass bands, suggesting that the effect of having or lacking experience in other musical genres may be minor among BMs.

**Reviewer #2:**

*** Limitations:**

- Pages 27-28, lines 615-625: An alternative explanation may be related to the choice of regional gold prize awarded brass bands (see above, Comment on pages 10-11, lines 220-223).

**Author reply:** Please see our responses above to your comments. We have added limitations to mention that an alternative explanation may be related to the choice of regional gold prize-awarded brass bands.

**Author action:** Please see the limitation section in the revised discussions.

**Reviewer #2:**

- Page 27, line 606: I suggest you to use "it would be important" rather than “it is important”

**Author reply:** We corrected the sentence.

**Author action:**

Line 610: “Taken together, it would be~~is~~ important to test…”

**Reviewer #2: 2-5. References:**

- Page 32, line 729: There is a typing error for the reference 21 (“Colledge”).

**Author reply:** We corrected the typing error.

**Author action:** Please see below:

Line 752-753: 21. Scannell DA. Sound Judgment : Auditory – but not Visual – Information Reveals Musical Competition Winners. Boston College~~Colledge~~, 2014 pp. 1–27. http://hdl.handle.net/2345/3867

**Reviewer #2: 2-6. Figures:**

* Figure 1:

This figure helps clarify the experimental protocol used. However, for each condition, I suggest you shift the "Judgement" rectangle and the word "Response" to the right, so as to make the "Stimuli 3" rectangle more visible. Furthermore, to illustrate the randomization in order of presentation of stimuli, you should alternate the location of the qualified group across sets (in 1st, 2nd or 3rd position).

**Author reply:** We have revised the Fig. 1 according to your comments.

**Author action:**

Please see the revised figure below.

**Reviewer #2: 3. Supplementary information:**

* Table S1: For the title, could add “for assessing the normality of data”.

**Author reply:** We added.

**Author action:**

Supp info, Line 2-3: Table S1. Results of the Shapiro-Wilk test and the values of kurtosis and skewness for assessing the normality of data.

**Reviewer #2:**

* Supplementary Texts: I think there was a confusion (or an error) in the numbering of the additional texts: S1 Text is missing in the Supplementary Information document and S4 Text is missing in the revised manuscript (only mentioned in the Response to Reviewers). Please correct this point and then check the consistency of the numbering between the manuscript and the supplementary information.

**Author reply:** We checked the consistency of the numbering between the manuscript and the supplementary information.

**Author action:** We corrected the numbering of the texts in the supplementary information document.

**Reviewer #2:**

* S1 Figure: The caption should be in normal character (line 26). Please, add a space between “among” and “brass” (line 27).

**Author reply:** We added a space.

**Author action:**

Supp info, Line 29: …brass band competitions among brass band musicians…

**Reviewer #2:**

* S3 Text (S2 Text ?): The title should be in bold (line 29).

**Author reply:** We have changed the title to bold.

**Author action:**

Supp info, Line 31: **S2~~3~~ text. Criteria for Choosing the Experiment Stimuli**.

**Reviewer #2:**

* S4 Text (S3 Text ?):

- For the rational, you should mentioned Bouhuys et al, 1995 (Bouhuys AL et al, Induction of depressed and elated mood by music influences the perception of facial emotional expressions in healthy subjects, J Affect Disord 1995).

**Author reply:** We mentioned Bouhuys et al., 1995 in the S3 Text.

**Author action:**

Supp info, Line 59-60: Previous studies evaluating facial emotional expressions have reported that the gender of the evaluator can influence the results [1].

**Reviewer #2:**

- For the gender comparison, I suggested assessing the effect of gender (as independent variable) rather than the effect of each gender, on the observed results.

**Author reply:** We reconsidered your comments and tested the effect of gender by performing a two-way ANOVA and found that the main effect of gender and the interaction between gender and experimental condition were not significant..

**Author action:** We added following sentences.

Supp info, Line 62-67: The 2 (participants’ gender) × 3 (experimental conditions) two-way analysis of variance was conducted to examine the main effect of gender. The results indicated that there was no main effect of gender (F (1, 295) = 0.94, p = 0.33, η_p_^2^ = 0.003), nor was there an interaction between gender and experimental conditions (F (2, 295) = 0.63, p = 0.53, η_p_^2^ = 0.004). Therefore, it is considered unlikely that the gender of the evaluators influenced the results of the current experiment.

**Reviewer #2:**

- If possible, I thank you for carrying out a new analysis, on the same model as the analysis of the two sub-groups of BMs.

**Author reply:** Same as the response above, we reconsidered your comments and tested the effect of musical experience outside of brass bands by performing a two-way ANOVA and found that the main effect of musical experience and the interaction between musical experience and experimental condition were not significant.

**Author action:** We added following sentences.

Line 399-405: To examine the main effect of musical experience outside of brass bands in BMs, 2 (presence or absence of musical experience outside of brass bands) × 3 (experimental conditions) two-way analysis of variance was conducted. The results indicated that there was no main effect of musical experience (F (1, 0) = 0.0, p = 0.99, η_p_^2^ < 0.0001), nor was there an interaction between gender and experimental conditions (F (2, 714) = 1.42, p = 0.24, η_p_^2^ = 0.02). Therefore, it is considered unlikely that the music experience outside of brass bands influenced the results of the current experiment.

**Reviewer #2:**

- Lines 58 and 64: you should add “the” (among the three conditions)

**Author reply:** Due to the revision in the analysis method, the sentence has been removed.

**Reviewer #2:**

* S3 Figure:

- The caption should be in normal character (lines 74-76). 26). Please, add a space between “among” and “brass” (line 27).

**Author reply:** Due to the revision in the analysis method, the caption has been removed.

**Reviewer #2:**

- Lines 74-75: Please add “according to gender” (for each participant, according to gender)

**Author reply:** Due to the revision in the analysis method, the sentence has been removed.

**Reviewer #2:**

- Please explain the signification of the dagger mark for VO condition in female participants.

**Author reply:** Due to the revision in the analysis method, the figure has been removed.

**Reviewer #4:**

Thank you for the opportunity to review the manuscript. I believe it is sound and suitable for publication after correcting minor changes.

Below, I provide questions that I believe are pertinent to improving the manuscript.

**Author reply:** Thank you. We did our best to improve our manuscript based on your comments.

**Reviewer #4:**

The introduction covers the justification for the study well, however, I believe the authors could make it more succinct and objective.

**Author reply:** Based on your feedback, we revised our introduction to be more succinct and objective. For instance, we revised the first paragraph to concisely mention the findings of the previous study. We also made the last paragraph more concise. We chose to retain the detailed descriptions of previous findings on the sight-over-sound effect, as they are crucial for understanding the findings of this study.

**Author action:** Please see our revised introduction.

**Reviewer #4:**

I suggest that the authors could describe the methodology better, for example by adding a topic on the location of the study.

**Author reply:** Considering your comments, we revised the Methods section to better describe our methodology. Please refer to the Ethics Statement and Participants sections in the Methods.

**Reviewer #4:**

There is confusion about the topic of methods and results. The methods should include information that was used in the methodology of the study, the results found after collection should appear in another topic.

**Author reply:** Following your feedback, we revised the Methods and Results sections. We made an effort to describe the study's methodology in detail in the Methods section and to present the findings from data collection in the Results section. For instance, the “Demographic and musical training background” are now described in the Results section, as these details were obtained after data collection.

**Author action:** Please see the revised the Methods and Results sections. The “Demographic and musical training background” were described as:

Line 287-315: **Demographic and musical training background.**

The number of BMs had musical training experience in brass bands (mean = 8.07 years, SD = 5.37, range = 1−35 years, see Table 1 for BMs mean age of commencement of brass band training). A total of 137 BMs participated in brass band competitions, and 12 of them had participated in the highest competition levels (e.g., All Japan Brass Band Competition). Eighteen BMs reported having experience as brass band clinicians.

Among BMs, 118 participants had musical training other than brass bands training (mean = 10.98 years, SD = 8.79, range = 1−48 years, vocal: 6, keyboards: 96, strings: 3, wind instruments: 26, percussion: 2). The mean duration of musical training in NBMs was 11.72 years (SD = 7.81, range = 1−34 years, vocal: 15, keyboards: 39, strings: 14, wind instruments: 3, percussion: 3, Japanese traditional instruments: 4; see Table 1 for NBMs mean age of commencement of musical training).

The number of participants in each group and in each experimental condition was as follows: BMs: 171 (46 male participants and 125 female participants; AO: 52, VO: 58, AV: 61); NBMs: 78 (16 male participants, 62 female participants; AO: 32, VO: 21, AV: 25); and NMs: 52 (8 male participants and 44 female participants; AO: 21, VO: 18, AV: 13; see Table 1 for participant’s gender, mean age, standard deviation of age and age range; see Table 2 for number of participants in each experimental condition).

**Table 1. Demographics and Musical Training Background**

|  | Mean age (years) | Sex (F/M) | Mean duration of musical training (years) | Mean age of commencement of musical training (years) | Mean duration of brass-band training (years) | Mean age of commencement of brass-band training (years) |
| --- | --- | --- | --- | --- | --- | --- |
| Brass band musicians (n = 171) | 33.08 (11.57, 18-56) | 125/46 | 10.98 (8.79,  1-48) | 7.06  (4.88, 2-34) | 8.07 (5.37,  1-35) | 12.25 (1.88, 7-19) |
| Non-brass band musicians (n =78) | 35.39 (13.03, 18-58) | 62/16 | 11.72 (7.81, 1-34) | 7.35 (5.10, 2-28) |  |  |
| Non-musicians (n = 52) | 39.15 (8.73, 19-55) | 44/8 |  |  |  |  |

SD and range in parentheses.

**Table 2. Number of Participants in Each Experimental Condition**

|  | Audio-only condition | Visual-only condition | Audiovisual condition |
| --- | --- | --- | --- |
| Brass band musicians (n = 171) | 52 | 58 | 61 |
| Non-brass band musicians (n = 78) | 32 | 21 | 25 |
| Non-musicians (n = 52) | 21 | 18 | 13 |
| All participants (N = 301) | 105 | 97 | 99 |

**Reviewer #4:**

I think it would be interesting to provide data characterising the sample for the different groups (e.g. mean and standard deviation for females and males in each group).

**Author reply:** Please see our response above.

**Reviewer #4:**

With regard to the evaluation procedures, participants could only watch the videos once and could not go back to revisit the stimuli. This can limit the accuracy of judgements, especially if participants are unfamiliar with the procedure.

**Author reply:** We used this procedure (i.e., watching the videos only once and not being able to go back) based on a series of previous studies on the sight-over-sound effect [Tsay, 2013; 2014; Mehr et al., 2018; Chiba et al., 2023]. This was to maintain consistency with the experimental procedures used in those studies. To clarify this, we revised the sentence in the Methods section.

**Author action:**

Line 247-248: Based on the previous study [1, 20, 22, 23], ~~P~~participants were instructed to play each stimulus only once, and they were not allowed to return to the previous page.

**Reviewer #4:**

Was a sample calculated? what were the eligibility criteria for the sample? what inclusion and exclusion criteria were adopted? Who was responsible for recruiting the sample? Make it clear whether the sample was carried out by convenience.

**Author reply:** We revised our method section and added information on how to set the sample size, inclusion and exclusion criteria in the Ethics statement section.

**Author action:**

Line 159-165: Inclusion criteria~~Participants~~ were individuals aged 18 and over who are native Japanese speakers aged 18 and over who are native Japanese speakers, without any hearing or visual impairments. Based on previous research [1], the sample size was estimated~~set~~ to approximately 300 participants. Participants were recruited from Keio University and its surrounding areas, utilizing a combination of social media and word-of-mouth advertising. We endeavored to ensure a balanced representation of individuals with brass band and music experience by broadly soliciting participants.

**Reviewer #4:**

Using more robust tests (e.g. ANCOVA) could help to understand confounding variables, such as the imbalance between female and male gender in the groups analysed. Another suggestion would be to calculate effect sizes (e.g. eta square for Kruskal-Wallis, r for Wilcoxon) to quantify the magnitude of the differences observed between the experimental conditions and groups of participants.

**Author reply:** Thank you for your valuable feedback. We performed an additional analysis using ANCOVA, with gender set as a covariate. As a result, we confirmed that the findings were consistent with those obtained from the ANOVA. Since we believe these results strengthen our conclusions, we have added the ANCOVA results to the Supplementary Information.

**Author action:**

Supp Info Page 8, Line 68-80:

We also conducted ANCOVAs to rule out the effect of gender on accuracy rates in our experiment. The ANCOVA model included the dependent variable (accuracy rate), the independent variable (experiment condition), and gender as the covariate. For ALLs, the ANCOVA revealed that the condition had a significant effect on the accuracy rates after controlling for participants’ gender (F(2, 1948) = 3.65, p = 0.027, partial η² = 0.02). For BMs, the ANCOVA revealed no significant effect on the accuracy rates after controlling for participants’ gender (F(2, 255) = 0.51, p = 0.60, partial η² = 0.006). For NBMs, the ANCOVA revealed that the condition had a significant effect on the accuracy rates after controlling for participants’ gender (F(2, 5711) = 13.10, p < 0.001, partial η² = 0.26). For NMs, the ANCOVA revealed no significant effect on the accuracy rates after controlling for participants’ gender (F(2, 658) = 1.005, p = 0.37, partial η² = 0.04). These ANCOVA analyses, which included gender as a covariate, yielded results consistent with those obtained without including the covariate. Therefore, the influence of gender on the findings of this study is considered to be minor.

**Reviewer #4:**

I hope that my considerations will help to improve the final manuscript.

Sincerely.

**Author reply:** Thank you for your valuable comments. We did our best to improve the manuscript based on your comment.
