# Supplementary material for: Sight-over-sound effect depends on interaction between evaluators’ musical experience and auditory-visual integration: An examination using Japanese brass band competition recordings
Source: PLoS One. 2025 Apr 29;20(4):e0321442. doi: 10.1371/journal.pone.0321442 (PMC12040236; doi:10.1371/journal.pone.0321442)
Supplement: Attachment — Submitted filename: Response to Reviewers_round3.docx [file pone.0321442.s005.docx]

**Manuscript ID:** PONE-D-23-19525R3

**Type:** Research Article

**Title:** Sight-over-sound effect depends on evaluator’s musical experience: An examination using Japanese brass band competition recordings

**Author:** Samma *et al*

**To:** Dr. Thiago P. Fernandes, Academic Editor PLOS ONE

Dear Dr. Fernandes,

**Re: Reply to reviewers on PONE-D-23-19525R2**

Thank you very much for your review of our submission, manuscript PONE-D-23-19525R3, entitled “Sight-over-sound effect depends on interaction between evaluators’ musical experience and auditory-visual integration: An examination using Japanese brass band competition recordings,” which we submitted to PLoS ONE.

In this letter, we summarize how we addressed the comments made by the reviewers. We found the reviews to be extremely helpful and have done our best to utilize them to strengthen the paper.

Below, we have provided a detailed explanation of how we addressed each of the comments made by the reviewers.

We greatly appreciate your efforts as an editor of the journal and your contribution to improving our manuscript.

Thank you very much.

Tomohiro Samma, Kazuaki Honda PhD, and Shinya Fujii PhD

**Reviewers’ Comments to the Author:**

**Editor:** Please double-check grammar (e.g. punctuation and wording);

**Author reply:** Thank you for your comment. We have checked the grammar throughout the manuscript. In addition, we have had them manuscript copyedited by a professional scientific editing service.

**Editor:** Please double-check refs (e.g. abbreviations, page numbers, and sentence case need to be corrected);

**Author reply:** Thank you for your comment. We have checked all references and made revisions where required.

**Editor:** Although the title is not concerning, I believe the authors could improve by focusing on the novelty and importance of the study. Objective phrasing, such as highlighting the interaction between musical experience and sensory judgement, would engage a broader audience. For example, using terms like 'auditory-visual' could improve clarity, or elaborating on how musical experience influences sensory perception or performance would be beneficial;

**Author reply:** Thank you for your comment. We have revised the title to better highlight the interaction between musical experience and sensory judgment. The title has been revised to: “**Sight-over-sound effect depends on interaction between evaluators’ musical experience and auditory-visual integration: An examination using Japanese brass band competition recordings.**”

**Editor:** Consider refining the Abstract, particularly in terms of language and how the information is presented. The authors are strongly encouraged to make it more engaging, emphasizing the novelty and relevance of the research;
- The introductory lines should be revised to highlight the importance of the s-o-s in performance evaluations;
- Consider making the methods and results subsections more distinct;
- Elaborate slightly on why the absence of this effect in brass band musicians and non-musicians is important;
- The abstract could more strongly highlight why this study fills an important gap in understanding multisensory integration in evaluative sensory (assessment?);
- Some phrasing could be simplified to make the Abstract more direct;
- Additionally, strengthen the Abstract by reinforcing the importance of the findings and making the practical implications of the study more explicit;

**Author reply:** Thank you for your detailed feedback on refining the Abstract. We have revised the introduction to emphasize the importance of the sight-over-sound effect in performance evaluation (lines 23-25). Additionally, we have clearly stated the objective of this study in relation to previous research and highlighted its novelty (lines 25-28). The methods and results sections have been clarified by distinguishing the subgroups more explicitly (lines 28-34), and we have discussed the significance of the absence of the sight-over-sound effect in BMs and NMs (lines 34-38). Furthermore, we have explicitly stated that our findings contribute to filling a critical gap in multisensory integration research by elucidating how differences in musical experience influence evaluative processes across sensory modalities (lines 38-40).

**Editor:** Expand the discussion on how musical expertise may influence the sight-over-sound effect. Incorporating cognitive and perceptual theories could provide a deeper theoretical framework for Hypothesis 2, especially regarding how experts process auditory and visual cues differently;

**Author reply:** Thank you for your valuable suggestion. Based on previous studies indicating that musicians exhibit greater selective attention to auditory information than non-musicians [48, 49], we have noted that BMs, with their brass band training experience, may possess superior selective auditory attention abilities compared to NBMs and NMs.

**Editor:** The transition between sections, such as from the summary of previous findings to the Introduction of the study, could be made smoother;

**Author reply:** Thank you for your comment. We have revised the transition between sections throughout the manuscript. In particular, we have rewritten the transition from the summary of previous findings to the aim of the present study to improve the flow of the Introduction (lines 140–146).

**Editor:** Including more references to studies on the influence of visual expressiveness and non-verbal cues on performance judgements would strengthen the arguments;

**Author reply:** Thank you for your suggestion. We added more references to studies on the influence of visual expressiveness and non-verbal cues on performance judgements [3, 4, 8, 9, 14].

**Editor:** More detail could be provided on how musical training might influence sensory integration in music performance. A brief discussion on perceptual training would also be of interest;

**Author reply:** Thank you for your comment. We have added a discussion citing previous studies reporting that musicians possess advanced auditory processing abilities [40,41] and demonstrate higher accuracy in pitch [42-44] and rhythm perception [45-47] compared to non-musicians. Additionally, we have noted that auditory information processing developed through musical training may influence the sight-over-sound effect.

**Editor:** The Methods section is a little bit worrying. I'd highly encourage the authors to consider:

- A detailed justification for the chosen sample size is required. Please consider performing a power analysis to ensure the sample size is appropriate;

- More refs and details from prior studies that used similar sample size calculations are necessary;

**Author reply:** Thank you for your valuable feedback. We have added a *Statistical Power* subsection to include the results of our power analysis (lines 195-200). Additionally, we have provided details on sample size calculations and effect sizes from previous studies.

**Editor:**

- Clarify how the bands were selected (e.g. randomly or based on performance level) and provide further details on the scoring and ranking system used in the competition;

**Author reply:** Thank you for your valuable comments. We have clarified that the brass bands were selected based on their performance level (lines 225-237) and have added a detailed explanation of the scoring and ranking system used in the competition (lines 220-224).

**Editor:**

- Differences of demographics - particularly gender - need to be addressed in the analysis. Please clarify whether gender was included as a covariate or if a gender interaction analysis was conducted to ensure it did not influence the results;

**Author reply:** Thank you for your insightful comment. We have decided to move the explanation about the impact of gender differences on the sight-over-sound effect, previously included in the Supporting Information, to the Results section of the manuscript (lines 483-499). We conducted a two-way ANOVA with sex and experimental condition as factors, as well as an ANCOVA with accuracy rate as the dependent variable, experimental condition as the independent variable, and sex as a covariate. In both analyses, we confirmed that participants’ sex had no effect on the results of this study.

**Editor:**

- The reasons for grouping participants (BMs, NBMs, and NMs) need more explanation. Please explain why these cutoff points were used and consider adding more detailed information on groupings;

**Author reply:** Thank you for your valuable feedback. In this study, we hypothesized that experience with a specific musical genre could influence the sight-over-sound effect. Therefore, we categorized participants into three groups: BMs, who had direct experience with brass band music; NBMs, who had general musical experience but no direct brass band experience; and NMs, who had no formal musical training beyond compulsory education. This classification was implemented to examine the impact of brass band experience on performance evaluation. We have included this explanation in the *Participant Grouping* subsection (lines 207-215).

**Editor:**

- While participant recruitment via social media and word-of-mouth is mentioned, there is no discussion of how recruitment bias was controlled. Please provide further elaboration;

**Author reply:** Thank you for your comment. We have added a discussion on the limitations of recruitment bias in online experiments as the first limitation of this study (lines 676-678).

**Editor:**

- The choice of a 6-second stimulus length needs more explanation. Please explain how the stimuli were randomized across the three conditions (AO, VO, AV) to avoid bias. Also, give more details on how you kept the stimulus presentation consistent, especially since participants may have used different equipment;

**Author reply:** Thank you for your comment. We have explained the rationale for selecting a 6-second stimulus length by referencing Ambady et al.‘s “thin-slices” research [51] (lines 242-246). Additionally, we have provided details on the method used to randomize the stimuli across conditions (lines 252-256). Furthermore, we have acknowledged the potential variation in participants’ equipment as the first limitation of this study (lines 678-681).

**Editor:**

Some aspects for the Results:

- Ensure consistent rounding across all data. For example, round p = 0.021 to p = 0.02 or use three decimal places throughout for precision;* Table S1: For the title, could add “for assessing the normality of data”.

**Author reply:** Thank you for your comment. We have rounded all the data to two decimal places (e.g. *p* = 0.07 instead of *p* = 0.067).

**Editor:**

- Format the confidence intervals with commas to clearly separate the lower and upper bounds (e.g. [35.14, 43.71] instead of [35.14 43.71]);

**Author reply:** Thank you for your comment. We have formatted the confidence intervals with commas to clearly separate the lower and upper bounds.

**Editor:**

- Present all medians in the same format, with or without decimals;

**Author reply:** Thank you for your valuable comment. We formatted all medians without decimals (e.g. 40 instead of 40.0)

**Editor:**

- Report effect sizes for each pairwise comparison (e.g., AO vs. VO) instead of only for the Kruskal-Wallis test;

**Author reply:** Thank you for your valuable comment. We added effect sizes for each pairwise comparison (lines 401-402; 445-446; 458-461).

**Editor:**

- Clarify the main effect of musical experience, as zero degrees of freedom is unusual and may be a typo;

**Author reply:** Thank you for your valuable comment. It was a typo and we revised the result (line 485).

**Editor:**

- In cases where the lower bound of an effect size CI is 0, it may indicate no effect. The effect size and its confidence intervals should be checked to ensure they align with the conclusion of significance;

**Author reply:** Thank you for your suggestion. We have added that the lower bound of the effect size CI is 0, indicating the possibility that this result may not reflect a meaningful effect (lines 460-461).

**Editor:**

- Consider using Hedges' g for pairwise comparisons and ω² for ANOVA to give more reliable effect size estimates;

**Author reply:** Thank you for your comment. We use ω² for ANOVA (lines 484-486).

**Editor:**

The inclusion of effect sizes is commendable, but they could be discussed more explicitly in the context of their implications for the findings. This would offer a deeper understanding of the practical significance of the results;

**Author reply:** Thank you for your suggestion. We have noted that the effect sizes calculated for the comparisons between conditions in NBMs were relatively large (*η*^2^ = 0.53), indicating that the observed differences in NBMs were meaningful (lines 512-516).

**Editor:**

The non-significant results for certain conditions (e.g., AO and AV for ALLs, BMs, and NMs) could use more explanation. Consider briefly discussing possible confounding factors or limits, like visual or auditory dominance, in this group;

**Author reply:** Thank you for your suggestion. We have noted as the fourth limitation of this study, as well as an open question for future research, that the accuracy rates in the AV condition did not surpass those in the AO or VO conditions. Based on previous studies suggesting that the combination of auditory and visual information allows for a more accurate evaluation of a performer’s emotional intent [80,81], we have expanded the discussion accordingly (lines 699-709).

**Editor:**

The analysis shows no significant effect of musical experience outside brass bands. However, consider expanding on this. Is there research that explains why outside musical experience won't affect judgment accuracy in this case? Explaining this would strengthen your interpretation;

**Author reply:** Thank you for your suggestion. We have expanded the discussion to strengthen the validity of our interpretation by citing previous studies that report how experience with specific music genres shapes neural responses to musical features [63] and influences sensorimotor synchronization and timing perception [64] (lines 615-622).

**Editor:**

Expand the discussion to include other visual factors, like performer interactions, that might influence the sight-over-sound effect, not just camera angles;

**Author reply:** Thank you for your comment. As suggested, we have included a discussion on the potential influence of visual factors, such as performer interactions, on the sight-over-sound effect (lines 596-606).

**Editor:**

Provide more detailed explanations for NMs' performance. Consider alternatives, like relying on basic visual cues due to a lack of musical knowledge;

**Author reply:** Thank you for your comment. As you suggested, we have added a statement discussing the possibility that NMs relied on basic visual cues for evaluation due to their lack of musical knowledge (lines 668-672). Additionally, we have included citations to relevant previous studies.

**Editor:**

The study's use of only gold-prize brass bands is noted, but the explanation for why this neutralized auditory information is unconvincing. Expand this reasoning with more evidence or theoretical support;

**Author reply:** Thank you for your valuable comment. In our study, we aimed to minimize differences in performance level (auditory information) when selecting stimuli. However, since brass band competition rankings are not publicly disclosed, it is difficult to objectively confirm that performance-level neutralization was fully achieved. Nevertheless, the chance-level accuracy observed in the auditory-only condition (e.g., ALLs data) suggests that participants were unable to systematically identify performance differences based on auditory information alone, implying that the influence of auditory cues was effectively minimized. We have added this discussion as the fifth limitation of our study (lines 710-721).

**Editor:**

Discuss how a within-subjects design might affect results, considering factors beyond learning effects, like order effects or familiarity with specific pieces;

**Author reply:** Thank you for your comment. We have noted the lack of a within-subject design as the second limitation of this study (lines 685-692). In that section, we have added a statement highlighting the need to consider order effects and familiarity with specific musical pieces (lines 687-690).

**Editor:**

Overall, the discussion needs a clearer structure. Differ sections on limitations, rationale research, and hypotheses separately. Some sections seem repetitive, and transitions are unclear. Strengthen claims about attentional focus, visual dominance, and auditory skills by adding more empirical evidence;

**Author reply:** Thank you for your suggestions. Given that the study specifically involves an investigation into the replicability of the sight-over-sound effect, we have structured the Discussion to focus on the differences and similarities between our findings and those of previous studies. As well as subsections discussing these differences and similarities, we have included separate limitations and conclusions subsections to clearly structure the Discussion section. We have also added text (with appropriate citations) to strengthen the claims about attentional focus (lines 580-581), visual dominance (lines 660-666), and auditory skills (lines 635-641).

**Editor:**

In addition, consider updating refs, including those by Zamm, Bigand, and others that explain how musical training affects motor-cortical plasticity, providing more modern insights than earlier work.

**Author reply:** Thank you for your suggestion. We have added a description of the potential influence of performer-specific behaviors derived from ensemble experience on performance evaluation (lines 606-610) and included appropriate citations.
