# Supplementary material for: Sight-over-sound effect depends on interaction between evaluators’ musical experience and auditory-visual integration: An examination using Japanese brass band competition recordings
Source: PLoS One. 2025 Apr 29;20(4):e0321442. doi: 10.1371/journal.pone.0321442 (PMC12040236; doi:10.1371/journal.pone.0321442)
Supplement: Peer review history

# Author comment: Author response to Decision Letter 0

**DOI:** 10.1371/journal.pone.0321442.r001

5 Jul 2023

# Peer review: Decision Letter 0

**DOI:** 10.1371/journal.pone.0321442.r002

**Authors:** Thiago P. Fernandes

11 Sep 2023

PONE-D-23-19525Replicability of the sight-over-sound effect in the judgment of brass band competitions in JapanPLOS ONE

Dear Dr. Samma,

Thank you for submitting your manuscript to PLOS ONE. After careful consideration, we feel that it has merit but does not fully meet PLOS ONE’s publication criteria as it currently stands. Therefore, we invite you to submit a revised version of the manuscript that addresses the points raised during the review process.

==============================

This is a very interesting study, and the reading, for most parts, was fast-paceded. I appreaciated the text; however, as I read through it, also I noticed some aspects that could be refined. Hope the authors find these comments, and all of the raised points, helpful in some way.

Abstract

Some sentences in the Abstract require a little bit of work to enhance clarity and flow. For example, sentences like "a crucial ...," "background: brass band...," or "that the dominance" can be rephrased. Also I think the authors can set more details into their sample (age SD) and the stats values in Abstract;

Introduction

- The section has clarity and a swift pace, but there are opportunities for additional information, (a) explain how the study builds upon past findings and addresses their limitations, (b) a concise overview of findings, supported by data and their specifics (e.g. you can calculate ORs or even just provide data from the studies), and (c) emphasise the relevance of your study;

- The second paragraph effectively illustrates previous findings but would benefit from offering more interpretations and explaining how these findings built the design or rationale;

- Also, in the third paragraph, place the issues to be investigated in a broader context, underlining their importance;

- Clarify the relevance and implications of studying the s-o-s-e. Why is this relevant? Authors need to place more value in their study; 

- Consider reframing 4-5s paragraphs to ensure smoother transitions and clarity;

- Explain how previous studies on AVP contribute to understanding the sight-over-sound effect;

- Consider exploring theoretical frameworks that can elucidate the influence of musical experience on AVs;

- Ensure logical coherence in the hypotheses, particularly in explaining how the sound-over-sight effect is 'dependent' on musical experience and its implications. Overall, the authorss need to provide what they expected;

Methods

- Please extend and detail your eligibility criteria.  For instance, include specific conditions or criteria for exclusion, with references to their assumptions;

- Describe the efforts made to ensure the representativeness of the sample for better generalisability. I understand this is not the main point of the text, but this could be really important for other researchers and readers;

- Address potential biases or limitations in the procedure, as these may impact the interpretation of results;

- Please avoid placing stats outcomes within this section;

- Actually, the authors could consider examine skewness and kurtosis as most important parameters to normality;

- Provide effect sizes and CIs (i.e. I think you can use MBESS or even the rcompanion for R);

Discussion

- Consider reframing paragraphs 2 and 3 to refine readability;

- Debate on stimulus control can be more concise;

- Please check "variations in performance level" and link to your main rationale;

- Consider extend the debate on theories and your findings;

==============================

Please submit your revised manuscript by Oct 26 2023 11:59PM. If you will need more time than this to complete your revisions, please reply to this message or contact the journal office at plosone@plos.org . When you're ready to submit your revision, log on to https://www.editorialmanager.com/pone/ and select the 'Submissions Needing Revision' folder to locate your manuscript file.

Please include the following items when submitting your revised manuscript:

- A rebuttal letter that responds to each point raised by the academic editor and reviewer(s). You should upload this letter as a separate file labeled 'Response to Reviewers'.
- A marked-up copy of your manuscript that highlights changes made to the original version. You should upload this as a separate file labeled 'Revised Manuscript with Track Changes'.
- An unmarked version of your revised paper without tracked changes. You should upload this as a separate file labeled 'Manuscript'.

If you would like to make changes to your financial disclosure, please include your updated statement in your cover letter. Guidelines for resubmitting your figure files are available below the reviewer comments at the end of this letter.

If applicable, we recommend that you deposit your laboratory protocols in protocols.io to enhance the reproducibility of your results. Protocols.io assigns your protocol its own identifier (DOI) so that it can be cited independently in the future. For instructions see: https://journals.plos.org/plosone/s/submission-guidelines#loc-laboratory-protocols . Additionally, PLOS ONE offers an option for publishing peer-reviewed Lab Protocol articles, which describe protocols hosted on protocols.io. Read more information on sharing protocols at https://plos.org/protocols?utm_medium=editorial-email&utm_source=authorletters&utm_campaign=protocols .

We look forward to receiving your revised manuscript.

Kind regards,

Thiago P. Fernandes, PhD

Academic Editor

PLOS ONE

Journal requirements:

When submitting your revision, we need you to address these additional requirements.

1. Please ensure that your manuscript meets PLOS ONE's style requirements, including those for file naming. The PLOS ONE style templates can be found at

https://journals.plos.org/plosone/s/file?id=wjVg/PLOSOne_formatting_sample_main_body.pdf and

https://journals.plos.org/plosone/s/file?id=ba62/PLOSOne_formatting_sample_title_authors_affiliations.pdf

2. We note that the grant information you provided in the ‘Funding Information’ and ‘Financial Disclosure’ sections do not match.

When you resubmit, please ensure that you provide the correct grant numbers for the awards you received for your study in the ‘Funding Information’ section.

3. Thank you for stating the following in the Competing Interests section:

“K.H. is employed by the NTT Communication Science Laboratories, Nippon Telegraph and Telephone Corporation, Japan. The remaining author has no relevant financial or non-financial interests to disclose.

S.F. has received a Grant-in-Aid for Young Scientists B and a Grant-in-Aid for Scientific Research B from JSPS and research grants from JST COI-NEXT and Keio University Academic Development Funds. S.F. has also received research support from no new folk studio Inc. and Odakyu Railway within the past three years.”

Please confirm that this does not alter your adherence to all PLOS ONE policies on sharing data and materials, by including the following statement: "This does not alter our adherence to  PLOS ONE policies on sharing data and materials.” (as detailed online in our guide for authors http://journals.plos.org/plosone/s/competing-interests).  If there are restrictions on sharing of data and/or materials, please state these. Please note that we cannot proceed with consideration of your article until this information has been declared.

Please include your updated Competing Interests statement in your cover letter; we will change the online submission form on your behalf.

4. We note that you have indicated that data from this study are available upon request. PLOS only allows data to be available upon request if there are legal or ethical restrictions on sharing data publicly. For more information on unacceptable data access restrictions, please see http://journals.plos.org/plosone/s/data-availability#loc-unacceptable-data-access-restrictions.

In your revised cover letter, please address the following prompts:

a) If there are ethical or legal restrictions on sharing a de-identified data set, please explain them in detail (e.g., data contain potentially sensitive information, data are owned by a third-party organization, etc.) and who has imposed them (e.g., an ethics committee). Please also provide contact information for a data access committee, ethics committee, or other institutional body to which data requests may be sent.

b) If there are no restrictions, please upload the minimal anonymized data set necessary to replicate your study findings as either Supporting Information files or to a stable, public repository and provide us with the relevant URLs, DOIs, or accession numbers. For a list of acceptable repositories, please see http://journals.plos.org/plosone/s/data-availability#loc-recommended-repositories.

We will update your Data Availability statement on your behalf to reflect the information you provide.

5. We note that Figure 1 in your submission contain copyrighted images. All PLOS content is published under the Creative Commons Attribution License (CC BY 4.0), which means that the manuscript, images, and Supporting Information files will be freely available online, and any third party is permitted to access, download, copy, distribute, and use these materials in any way, even commercially, with proper attribution. For more information, see our copyright guidelines: http://journals.plos.org/plosone/s/licenses-and-copyright.

We require you to either (1) present written permission from the copyright holder to publish these figures specifically under the CC BY 4.0 license, or (2) remove the figures from your submission:

a. You may seek permission from the original copyright holder of Figure 1 to publish the content specifically under the CC BY 4.0 license.

We recommend that you contact the original copyright holder with the Content Permission Form (http://journals.plos.org/plosone/s/file?id=7c09/content-permission-form.pdf) and the following text:

“I request permission for the open-access journal PLOS ONE to publish XXX under the Creative Commons Attribution License (CCAL) CC BY 4.0 (http://creativecommons.org/licenses/by/4.0/). Please be aware that this license allows unrestricted use and distribution, even commercially, by third parties. Please reply and provide explicit written permission to publish XXX under a CC BY license and complete the attached form.”

Please upload the completed Content Permission Form or other proof of granted permissions as an "Other" file with your submission.

In the figure caption of the copyrighted figure, please include the following text: “Reprinted from [ref] under a CC BY license, with permission from [name of publisher], original copyright [original copyright year].”

b. If you are unable to obtain permission from the original copyright holder to publish these figures under the CC BY 4.0 license or if the copyright holder’s requirements are incompatible with the CC BY 4.0 license, please either i) remove the figure or ii) supply a replacement figure that complies with the CC BY 4.0 license. Please check copyright information on all replacement figures and update the figure caption with source information. If applicable, please specify in the figure caption text when a figure is similar but not identical to the original image and is therefore for illustrative purposes only.

6. Please include captions for your Supporting Information files at the end of your manuscript, and update any in-text citations to match accordingly. Please see our Supporting Information guidelines for more information: http://journals.plos.org/plosone/s/supporting-information.

[Note: HTML markup is below. Please do not edit.]

Reviewers' comments:

Reviewer's Responses to Questions

**Comments to the Author**

1. Is the manuscript technically sound, and do the data support the conclusions?

The manuscript must describe a technically sound piece of scientific research with data that supports the conclusions. Experiments must have been conducted rigorously, with appropriate controls, replication, and sample sizes. The conclusions must be drawn appropriately based on the data presented. 

Reviewer #1: Yes

Reviewer #2: Yes

Reviewer #3: Partly

**********

2. Has the statistical analysis been performed appropriately and rigorously? 

Reviewer #1: Yes

Reviewer #2: Yes

Reviewer #3: Yes

**********

3. Have the authors made all data underlying the findings in their manuscript fully available?

The PLOS Data policy requires authors to make all data underlying the findings described in their manuscript fully available without restriction, with rare exception (please refer to the Data Availability Statement in the manuscript PDF file). The data should be provided as part of the manuscript or its supporting information, or deposited to a public repository. For example, in addition to summary statistics, the data points behind means, medians and variance measures should be available. If there are restrictions on publicly sharing data—e.g. participant privacy or use of data from a third party—those must be specified.

Reviewer #1: Yes

Reviewer #2: Yes

Reviewer #3: Yes

**********

4. Is the manuscript presented in an intelligible fashion and written in standard English?

PLOS ONE does not copyedit accepted manuscripts, so the language in submitted articles must be clear, correct, and unambiguous. Any typographical or grammatical errors should be corrected at revision, so please note any specific errors here.

Reviewer #1: Yes

Reviewer #2: Yes

Reviewer #3: Yes

**********

5. Review Comments to the Author

Please use the space provided to explain your answers to the questions above. You may also include additional comments for the author, including concerns about dual publication, research ethics, or publication ethics. (Please upload your review as an attachment if it exceeds 20,000 characters)

Reviewer #1: General Comments:

This study is well written and deals with an important topic. However, the rationales are weak to support the hypotheses and some of the findings are explained in a vague manner without evidence. This part should be improved before accepted for publication. Specific comments are as follows.

Specific Comments:

• Line 107: The ‘sound-over-sight effect’ seems to be an error.

• Line 108-111: I suggest further explaining the rationale for H1 and H2. Readers may wonder the specific reasoning behind why the sight-over-sound effect is not replicable in the judgement of Japanese brass band competitions when camera angles and music pieces are controlled and why the sight-over-sound effect is dependent on the evaluator’s musical experience. The authors provided a citation for each hypothesis but specifying those reasoning may improve the quality of this manuscript.

• Line 134: The word, ‘subdivide’, means dividing something that has already been divided. This may need to be edited.

• Fig 1: I suggest clarifying the award information within performance, set, and group in the figure. It is not easy to digest this figure.

• Line 300: The ‘sound-over-sight effect’ seems to be an error.

• Line 397-414: The results of 1-sample Wilcoxon signed-rank tests should be moved to Results section from Discussion.

• Line 428-439: The interpretation of significant results seem to be conjecture, being described without any prior findings. Please discuss this finding in reference to prior findings.

Reviewer #2: 1. General comments:

The authors present an interesting experimental study on limited replicability of the sight-over-sound effect in the judgment of Japanese brass band performances.

There are no major concerns, but significant improvements are expected on the following points:

* For the Introduction part: an update of the references for the main topic would be useful;

* For the Methods part: although the study is well-designed, some additional details are necessary, in particular for experimental groups and stimuli choice;

* For the Results part: a reorganization of their presentation (currently distributed in different parts of the manuscript) is necessary, as well as a few additional tests;

* For the Discussion part: results analysis is well documented, but would need to be deepened, in particular by adressing the nature of the audiovisual interactions in the judgement task and the potential limitations of the study.

The manuscript is generally well written, but there are still some corrections to be made.

In conclusion, although the results presented may be important for the scientific community working on sensory processing, substantial revision is required before the manuscript can be accepted for publication.

In addition, regarding data availability, the experimental stimuli used are not directly available (but only upon request to the corresponding author). Given PLoS One policy on the matter, I don't know if this will constitute an obstacle to the acceptance of the manuscript.

2. Major revisions:

For page and line numbers, I will refer to the Word document (unless otherwise specified).

2-1. Introduction:

* Page 3 and following: I suggest to update your references on audiovisual interactions in music experience (e.g.: Moura et al, PLoS One 2023; Moura et al, NPJ Sci Learn 2023; Nusseck et al, Front Psychol 2022; Rozé et al, Sci Rep 2020) and comment the findings of these studies.

* Page 5, lines 90-91: I suggest that you support your argument with the results of the work of Schneider and his colleagues (Schneider et al, Ann N Y Acad Sci, 2005).

2-2. Methods:

* Participants:

- Page 8, lines 139, 144 and 156: For BMs, could you explain the mismatch between numbers of participants: 55 (line 144) vs 105 (line 156), given their total number, 171 (line 139)?

- Page 8, lines 148-152: This section should be placed at the beginning of the Results part (“There was no significant difference in…”)

* Stimuli:

- Page 9, lines 169-170: I suggest to briefly explain why you have chosen two gold prize awarded brass band, rather than silver or bronze prize awarded ones. This choice could be regarded as “no optimal” for your protocol.

- Pages 9-10, lines 170-174: Could you specify the whole number of available pieces and brass bands (e.g.: “the 10 experimental sets were chosen among…”)?

- Pages 10, lines 174-175: To be clearer, could you briefly explain (either here or in the Fig 1 caption) the criteria for choosing the experimental sets (i.e.: for a given competition, why only certain musical pieces have been selected?)

* Procedure (page 11, lines 2016-207): Please briefly specify the type of randomization used.

2-3. Results:

* Page 13, lines 239-240: At the beginning of this section, please add a first subpart on intergroup comparisons for demographic and musical training characteristics (currently on page 8, lines 148-152)

* Judgment experiment:

- For ALLs (page 13, lines 241-246): Did you also assess a potential effect of participants' gender (see, for instance: Bouhuys et al, J Affect Disord 1995)?

- For BMs (page 13, lines 253-258): Did you also assess a potential effect of other musical experience (than brass practice) in the participants of this group?

* Additional analysis:

- For ALLS and the three experimental groups (BMs, NBMs and NMs), could you verify that there are no intragroup differences for the number of participants between the three experimental conditions (AO, VO and AV)?

- It would be interesting to provide intergroup comparisons for overall performance and according to conditions (AO, VO, AV).

2-4. Discussion:

* On the whole, the discussion regarding the results obtained is well structured, but I think it would be important to deepen their analysis, by addressing:

- the nature of the audiovisual interactions in the judgement task, e.g. trying to explain the absence of “synergy” between auditory and visual cues (AV condition), whatever the group;

- the potential limitations of your study, notably the factors which may have interfered with the results obtained, e.g.: (i) potential interactions between the duration of stimuli and the perceptiveness of auditory and visual cues, (ii) the assessment of only one type of music ensemble, (iii) the fact that around 30% of subjects of the BM group had other musical experiences than brass practice (which may have interfered with the results obtained), and (iv) the high within-group variability of musical experience in the BM and NBM groups, i.e.: ranges of duration of musical training, age of onset of musical training, duration of brass ensemble training, and age of onset brass ensemble training (see: Shenker et al, Brain Struct Funct 2022).

* I also have some questions about and suggestion for the 4 explanatory factors you mentioned:

- Variation in performance level and genre (page 18, lines 351-354): Regarding the findings of Chiba and colleagues, couldn't these disparities also be linked to intercultural differences in movements induced by musical type, between Western classical music and Japanese shamisen music?

- Difference between solo and group performances (page 19, lines 370-390): Additionally, you could also mention the type of performed movements, more static (spatially speaking) for brass bands than for other types of ensembles (string orchestra, for example).

- Evaluator’s musical experience:

. page 20, lines 395-413: This subpart (for the test results) should be placed in the Results part.

. page 20, lines 413-414: These findings could be put into perspective with possible differences in the intergroup comparisons depending on the experimental conditions AO, VO and AV (see above).

. page 21, 425-427: Perhaps you could introduce in this subpart the notion of mirror neuron system, with their differential activation depending on the degree of familiarity with the observed action, musical practice and level of expertise? (e.g.: Baumann et al, Brain Res 2007; Calvo-Merino et al, Cereb Cortex 2005; Petrini et al, Neuroimage 2011).

Additionally, could you also discus an alternative hypothesis that auditory cues based judgement for brass music may be more accurate for BMs than for NBMs?

2-5. Conclusion:

The conclusion should open on some perspectives for future research (e.g., additional studies,…).

3. Minor revisions:

3-1. Tittle page:

* Main tittle (lines 1-2): I’d suggest a more "committed" title, e.g.: “Partial replicability”, “Limited replicability” or “Non-replicability of…”.

* Running tittle (lines 5-6): Please consider a more concise running title, for example by deleting “Replicability of the”.

* Author Contributions (lines 17-20): Please, avoid initial capital letters for common nouns.

* Keywords (PDF document):

If they will be used for the referencing of the paper, I’d suggest adding “audiovisual interactions” and/or “multisensory integration”.

3-2. Introduction:

* Page 3, line 44: please, add “conditions” at the end of the line (“between the two conditions”).

* Page 3, line 60: please, use lowercase letter for “the effect” (“different stimuli: the effect was”).

* Pages 4-5, lines 66-95: I’d suggest you list the 3 issues in a single paragraph (without line breaks).

* Page 5, line 89: I’d suggest to use “professional musicians” instead of “individuals with musical experience”.

* Page 5, line 103: I’d suggest to add “in controlled conditions” at the end of the sentence.

3-3. Methods:

* Ethics Statement (page 7, line 123): I’d suggest to add “no possibility” (“thereby ensuring no possibility of association”).

* Participants:

- Pages 7-8, first paragraph: For this subpart, you should avoid redundant information between text and Table 1 (e.g., lines 139-143).

- Page 8-lines 144-146: This sentence does not seem to me to be understandable; I’d suggest to use “other than” instead of “excerpt”.

- Page 8, lines 154-160: I’d suggest to move this paragraph before the previous one (starting at line 144).

* Stimuli (page 9, lines 164-166): I’d suggest to rephrase this sentence by using a verb.

* Procedure (page 11, lines 202-204): It would have been interesting to propose the same type of questions after the judgment tasks, in order to carry out a pre-post-test comparison and to look for possible correlations with the experimental results.

* Statistical analysis (page 12, lines 226): In this case, “To asses” would be more appropriate than “To confirm”.

3-4. Results:

* Judgment experiment:

- For ALLs (page 13, lines 242-245): You should presented the results in the same order as for Figure 2.

- For NBMs (page 14, lines 267-270): You should presented the results in the same order as for Figure 4.

- For NMs (pages 14-15, lines 284-287): You should presented the results in the same order as for Figure 5.

3-5. Discussion:

* Page 16, lines 296-297: I’d suggest to add “in controlled conditions” at the end of this introductory sentence.

* Page 16, lines 297-298: Perhaps you should use “main” or primary”, rather than “initial” hypothesis.

* Page 18, lines 346-347: Only one quote should be sufficient for this sentence.

* Page 21, lines 435-438: I’d suggest to add “auditory” (“or to precisely identify crucial auditory or visual cues…”).

3-6 Ending section:

* Data Availability (page 23, lines 463-465): If possible, I think it would be relevant to provide a set of the experimental stimuli used (in the three conditions: AO, VO and AV), in the supporting information files, in order to help the reader to better figure out the protocol.

3-7. References:

* Page 25, lines 515-516: The Reference 17 should be completed (type, university, web link).

4. Figures:

4-1. Figure 1:

* Caption (page 6, lines 186-195): To be clearer, could you briefly explain (either here or in the Stimuli part) the criteria for choosing the experimental sets?

5. Supplementary information

For the S1 Figure caption (lines 25-26), please consider the alternative proposition: “These graphs respectively represent…”

6. Additional references:

Baumann S et al, A network for audio-motor coordination in skilled pianists and non-musicians, Brain Res 2007

Bouhuys AL et al, Induction of depressed and elated mood by music influences the perception of facial emotional expressions in healthy subjects, J Affect Disord 1995

Calvo-Merino B et al, Action observation and acquired motor skills: an FMRI study with expert dancers, Cereb Cortex 2005

Moura N et al, Knee flexion of saxophone players anticipates tonal context of music, NPJ Sci Learn 2023

Moura N et al, The impact of visual display of human motion on observers' perception of music performance, PLoS One 2023

Nusseck M et al, Associations Between Ancillary Body Movements and Acoustic Parameters of Pitch, Dynamics and Timbre in Clarinet Playing, Front Psychol 2022

Petrini K et al, Action expertise reduces brain activity for audiovisual matching actions: an fMRI study with expert drummers, Neuroimage 2011

Rozé J et al, Cellists' sound quality is shaped by their primary postural behavior, Sci Rep 2020

Schneider et al, Structural, functional, and perceptual differences in Heschl's gyrus and musical instrument preference, Ann N Y Acad Sci, 2005

Shenker JJ et al, Early musical training shapes cortico-cerebellar structural covariation, Brain Struct Funct 2022

Reviewer #3: Here, the authors explored the classic “sight over sound” effect using a large sample with a well-controlled and novel stimulus set. They find that the effect is not reliably replicated and suggest that this is likely due to using such well-controlled stimuli. They also demonstrate that the effect may be somewhat dependent on musical experience.

Overall, the manuscript is very well written and thoughtful. However, there are a few sections where the authors should add clarity as I found them difficult to understand: (1) The methods, (2) stimuli, and (3) participant sections. For example, while the authors provided a graphic, it is not immediately apparent how many trials participants do, the number of pieces that are possibly assigned (eg, do all participants see all pieces of music?), whether the correct answer is always the same piece of music (it appears that the winners always performed the piece “March..”, etc. On Page 8, lines 144-148, what is this sentence referring to, given the context? What is 55 referring to, and why do the numbers not seem to sum properly? Therefore, and perhaps I’m misunderstanding, but I’m unsure how participants could select a winner when there appears to be a winner for only one musical piece. Were participants always presented with this piece on each trial? Or are they simply supposed to pick whichever performance got the highest rating given the three they are comparing them to?

Since the study is conducted between-subjects, and participants appear to often be at chance levels of performance, it seems plausible that one condition is better than another simply because those participants are better at any given task and not just the particular condition they are in. Furthermore, could the lack of an effect be driven by floor performance? In other words, could the presence of an effect for NBMs be due to that group of participants being better, overall, at any task (since it is my understanding that the VO group for NBMs are a separate group of people than those in the AO condition)? This is why an orthogonal task, to detect baseline levels of performance of a particular group is important. Did the authors include a separate task to get at this potential mechanism?

Similar to combining all participants into an ALL group, could the authors look at each stimulus and see its average accuracy dependent on the modality (AO, VO, and AV). This could tell us whether the stimuli were driving some of the observed differences in accuracy (eg, between BM and NBM, for AO and VO respectively) and whether the stimuli are somewhat responsible for the sight over sound effect for NBMs. In short, could it be that the stimuli are driving the effect for NBMs instead of the purported differences in mental imagery described in the discussion? Similarly, could the “above chance performance” for both BMs and NBMs in AO and VO respectively be, alternatively, due to the stimuli themselves? And not due to any internal mechanism? All of this is relevant since it seems that participants only had one interaction with each stimulus, which could lead to a lack of appropriate measurement power per stimulus. In other words, it could be the case that if participants saw each sample three times that they would get it wrong on one, but right on the other two occasions, demonstrating an overall ability to detect the correct answer.

Also, on line 402, the authors state that p = .021 is non-significant. Are they using a separate alpha here?

The authors conclude that, while they fail to replicate the effect with their general sample, they are able to replicate it within the NBMs and thus, the effect depends on musicianship. But I’m a bit unsure of whether that conclusion is warranted. Since, are they implicitly suggesting that the previously observed sight-over-sound effects were somehow secretly driven by musicians? And, in particular, musicians who were not explicit experts in the sub-domain of musicianship used as stimuli in a particular experiment that did replicate the sight-over-sound effect? Basically, couldn’t their positive effect just be noise (ie, could this just be a false positive, Type I error)?

**********

6. PLOS authors have the option to publish the peer review history of their article (what does this mean? ). If published, this will include your full peer review and any attached files.

If you choose “no”, your identity will remain anonymous but your review may still be made public.

**Do you want your identity to be public for this peer review?** For information about this choice, including consent withdrawal, please see our Privacy Policy .

Reviewer #1: No

Reviewer #2: **Yes: ** Dr Xavier PERROT

Reviewer #3: No

**********

[NOTE: If reviewer comments were submitted as an attachment file, they will be attached to this email and accessible via the submission site. Please log into your account, locate the manuscript record, and check for the action link "View Attachments". If this link does not appear, there are no attachment files.]

While revising your submission, please upload your figure files to the Preflight Analysis and Conversion Engine (PACE) digital diagnostic tool, https://pacev2.apexcovantage.com/ . PACE helps ensure that figures meet PLOS requirements. To use PACE, you must first register as a user. Registration is free. Then, login and navigate to the UPLOAD tab, where you will find detailed instructions on how to use the tool. If you encounter any issues or have any questions when using PACE, please email PLOS at figures@plos.org . Please note that Supporting Information files do not need this step.

# Author comment: Author response to Decision Letter 1

**DOI:** 10.1371/journal.pone.0321442.r003

15 Apr 2024

Manuscript ID: PONE-D-23-19525

Type: Research Article

Title: Sight-over-sound effect depends on evaluator’s musical experience: An examination using Japanese brass band competition recordings

Author: Samma et al

To: Dr. Thiago P. Fernandes, Academic Editor PLOS ONE

Dear Dr. Fernandes,

Re: Reply to reviewers on PONE-D-23-19525

Thank you very much for your review of our submission, manuscript PONE-D-23-19525 entitled “Replicability of the sight-over-sound effect in the judgement of brass band competitions in Japan”, which we submitted to PLoS ONE.

In this letter we summarize how we addressed the comments made by the reviewers. We found the reviews to be extremely helpful, and have done our best to utilize them to strengthen the paper.

Below, we have provided a detailed explanation of how we addressed each of the comments made by the reviewers.

We greatly appreciate your efforts as an editor of the journal and your contribution to improving our manuscript.

Thank you very much.

Tomohiro Samma, Kazuaki Honda, and Shinya Fujii PhD

---

Journal requirements:

When submitting your revision, we need you to address these additional requirements.

---

1. Please ensure that your manuscript meets PLOS ONEs style requirements, including those for file naming. The PLOS ONE style templates can be found at

https://journals.plos.org/plosone/s/file?id=wjVg/PLOSOne_formatting_sample_main_body.pdf and

https://journals.plos.org/plosone/s/file?id=ba62/PLOSOne_formatting_sample_title_authors_affiliations.pdf

Author reply: We have prepared the revised manuscript in accordance with PLOS ONE's style requirements, including the rules for file naming.

---

2. We note that the grant information you provided in the Funding Information and Financial Disclosure　sections do not match.

When you resubmit, please ensure that you provide the correct grant numbers for the awards you received for your study in the Funding Information section.

Author reply: Thank you for pointing that out. We have corrected the information to ensure consistency between the "Funding Information" and "Financial Disclosure" sections.

---

3. Thank you for stating the following in the Competing Interests section:

K.H. is employed by the NTT Communication Science Laboratories, Nippon Telegraph and Telephone Corporation, Japan. The remaining author has no relevant financial or non-financial interests to disclose.　S.F. has received a Grant-in-Aid for Young Scientists B and a Grant-in-Aid for Scientific Research B from JSPS and research grants from JST COI-NEXT and Keio University Academic Development Funds. S.F. has also received research support from no new folk studio Inc. and Odakyu Railway within the past three years.

Please confirm that this does not alter your adherence to all PLOS ONE policies on sharing data and materials, by including the following statement: This does not alter our adherence to PLOS ONE policies on sharing data and materials. (as detailed online in our guide for authors　http://journals.plos.org/plosone/s/competing-interests). If there are restrictions on sharing of data and/or materials, please state these. Please note that we cannot proceed with consideration of your article until this information has been declared.

Please include your updated Competing Interests statement in your cover letter; we will change the online submission form on your behalf.

Author reply: Thank you for your guidance. We confirm that these competing interests do not alter our adherence to all PLOS ONE policies on sharing data and materials.

---

4. We note that you have indicated that data from this study are available upon request. PLOS only allows data to be available upon request if there are legal or ethical restrictions on sharing data publicly. For more information on unacceptable data access restrictions, please see http://journals.plos.org/plosone/s/data-availability#loc-unacceptable-data-access-restrictions.

In your revised cover letter, please address the following prompts:

a) If there are ethical or legal restrictions on sharing a de-identified data set, please explain them in detail (e.g., data contain potentially sensitive information, data are owned by a third-party organization, etc.) and who has imposed them (e.g., an ethics committee). Please also provide contact information for a data access committee, ethics committee, or other institutional body to which data requests may be sent.

b) If there are no restrictions, please upload the minimal anonymized data set necessary to replicate your study findings as either Supporting Information files or to a stable, public repository and provide us with the relevant URLs, DOIs, or accession numbers. For a list of acceptable repositories, please see http://journals.plos.org/plosone/s/data-availability#loc-recommended-repositories.

We will update your Data Availability statement on your behalf to reflect the information you provide.

Author reply: Thank you for your guidance. In this study, we used video recordings of brass band competitions, and the rights to these videos are owned by a third-party organization (All Japan Band Association). Also, videos contain identifiable information, raising the possibility that individual could be recognized from the footage. Therefore, we do not have permission to broadly publicize any part or all of the stimuli, and we have adopted a format where the stimuli are made available only upon request to the authors. We would appreciate your advice if there are any issues with this approach.

---

5. We note that Figure 1 in your submission contain copyrighted images. All PLOS content is published under the Creative Commons Attribution License (CC BY 4.0), which means that the manuscript, images, and Supporting Information files will be freely available online, and any third party is permitted to access, download, copy, distribute, and use these materials in any way, even commercially, with proper attribution. For more information, see our copyright guidelines: http://journals.plos.org/plosone/s/licenses-and-copyright.

We require you to either (1) present written permission from the copyright holder to publish these figures specifically under the CC BY 4.0 license, or (2) remove the figures from your submission:

a. You may seek permission from the original copyright holder of Figure 1 to publish the content specifically under the CC BY 4.0 license.

We recommend that you contact the original copyright holder with the Content Permission Form (http://journals.plos.org/plosone/s/file?id=7c09/content-permission-form.pdf) and the following text:

I request permission for the open-access journal PLOS ONE to publish XXX under the Creative Commons Attribution License (CCAL) CC BY 4.0 (http://creativecommons.org/licenses/by/4.0/). Please be aware that this license allows unrestricted use and distribution, even commercially, by third parties. Please reply and provide explicit written permission to publish XXX under a CC BY license and complete the attached form.

Please upload the completed Content Permission Form or other proof of granted permissions as an Other file with your submission.

In the figure caption of the copyrighted figure, please include the following text: Reprinted from [ref] under a CC BY license, with permission from [name of publisher], original copyright [original copyright year].

b. If you are unable to obtain permission from the original copyright holder to publish these figures under the CC BY 4.0 license or if the copyright holder requirements are incompatible with the CC BY 4.0 license, please either i) remove the figure or ii) supply a replacement figure that complies with the CC BY 4.0 license. Please check copyright information on all replacement figures and update the figure caption with source information. If applicable, please specify in the figure caption text when a figure is similar but not identical to the original image and is therefore for illustrative purposes only.

Author reply: Thank you for your guidance. We have created and replaced the previous figure with an alternative one that complies with the CC BY 4.0 license. The replaced figure does not contain any copyright-protected information and consists entirely of illustrations created by the authors.

---

6. Please include captions for your Supporting Information files at the end of your manuscript, and update any in-text citations to match accordingly. Please see our Supporting Information guidelines for more information: http://journals.plos.org/plosone/s/supporting-information.

Author reply: Thank you for your guidance. We have updated the caption information accordingly.

---

Editor's commentary: This is a very interesting study, and the reading, for most parts, was fast-paceded. I appreaciated the text; however, as I read through it, also I noticed some aspects that could be refined. Hope the authors find these comments, and all of the raised points, helpful in some way.

Author reply: Thank you for your encouraging words and the recognition of the value of our study. We are pleased to hear that you found the reading largely fast-paced and appreciable. Your feedback is invaluable, and we are dedicated to addressing all the points raised to refine the quality of the manuscript. We genuinely appreciate the constructive nature of your comments and are confident they will serve to improve the work.

---

Editor's commentary: Abstract

Some sentences in the Abstract require a little bit of work to enhance clarity and flow. For example, sentences like "a crucial ...," "background: brass band...," or "that the dominance" can be rephrased. Also I think the authors can set more details into their sample (age SD) and the stats values in Abstract;

Author reply: Thank you. We have rephrased the sentences you pointed out. Also, we have included more detailed information about the participants and statistical values in Abstract.

Author action:

Revised abstract: The sight-over-sound effect refers to the dominance of visual over auditory information in judging musical performances. Several studies have pointed out difficulties in replicating the sight-over-sound effect. Previous research has not conclusively determined if this effect persists when controlling for both the music piece and camera angles of the stimuli being compared. Additionally, the relationship between the effect and evaluators’ musical experience remains unclear. To address these gaps in current knowledge, we examined the replicability of the sight-over-sound effect using recordings of Japanese brass band competitions. A total of 301 adults (average age = 34.81 ± 11.71 years) participated in this study, and they were divided into three groups based on their experience with brass band and music. Among the participants, 171 were brass band musicians (n = 171; average age = 33.08 ± 11.57 years), 78 were non-brass band musicians (n = 78; average age = 35.39 ± 13.03 years), and 52 were non-musicians (n = 52; average age = 39.15 ± 8.73 years). We found that the sight-over-sound effect was notably present in non-brass band musicians (Kruskal-Wallis test: p < 0.001), but not in brass band musicians (p = 0.48) or non-musicians (p = 0.37). Thus, the sight-over-sound effect was limited and dependent on the evaluators’ musical experience. Our findings suggest that the evaluator's musical experience should be considered when investigating the sight-over-sound effect.

---

Editor's commentary: Introduction

- The section has clarity and a swift pace, but there are opportunities for additional information, (a) explain how the study builds upon past findings and addresses their limitations, (b) a concise overview of findings, supported by data and their specifics (e.g. you can calculate ORs or even just provide data from the studies), and (c) emphasise the relevance of your study;

Author reply: We appreciate the positive remarks on clarity and pace. To address your suggestions (a) – (c), we revised our introduction as follows:

Author action:

We added the sentences to explain (a) how the study builds upon past findings and addresses their limitations and (b) a concise overview of findings, supported by data and their specifics as follows.

Line 57-68: Regardless of musical experience, people consistently reported that auditory information is a more important source than visual information when evaluating musical performances (see Experiment 1 in [1]). Experiment 3 in [1] was conducted where participants watched 6-second excerpts of the 1st, 2nd, and 3rd-place performers’ recordings in international music competitions and were asked to select the actual winner from among them (chance level: 33.3%). The results reported that non-musicians were most accurate in correctly identifying the actual winner when evaluating based solely on visual-only (VO) stimuli (mean answering rate: 46.4%), compared to evaluating based on audio-only (AO) stimuli (28.8�) or audiovisual (AV) stimuli (35.4�). Similarly, it was also reported that even professional musicians were able to select the winner with high accuracy when evaluation based on VO stimuli (47.0%), as compared to AO stimuli (25.7%) and AV stimuli (29.5% see Experiment 5 in [1]).

Line 73-78: Mehr et al. [22] demonstrated that the sight-over-sound effect was replicable when using the same stimuli presented in Tsay’s [1] study. However, when the experiment was modified to a two-choice format (chance level: 50%), using a combination of performance stimuli from the winner and a preliminary round loser, the sight-over-sound effect was not observed (VO: 45.2%, AO; 68.4%, AV: 63.6%, see Experiment 3 in [22]).

We also added the sentences to emphasize (c) the relevance of our study.

Line 144-159: The first hypothesis was based on the prediction by Mehr et al. [22] that the sight-over-sound effect would not be replicated when controlling for stimulus information such as camera angles and musical pieces. In the previous studies by Tsay [1, 20] and Mehr et al. [22], it was reported that the accuracy rate in visual-only condition was significantly higher than chance level. In the current study, where the camera angle and musical pieces of the stimuli were controlled, it was hypothesized that the variability in visual information would be reduced, potentially leading to a lower accuracy rate in visual-only condition. We aimed to verify that the sight-over-sound effect is not replicated in the choice of winners by all participants, regardless of their brass band or musical experiences. The second hypothesis is based on the possibility that BMs, utilizing their brass band experience, might be able to select the actual winners with higher accuracy in audio-only and audiovisual conditions as compared to visual-only conditions. The stimuli used in our study consist of performance recordings with the same musical pieces, which may facilitate sound-based evaluations compared to previous studies where the musical pieces were inconsistent. It is hypothesized that BMs, drawing on their brass band experiences, can delicately evaluate the sound and predict the winners, potentially leading to a non-replicability of the sight-over-sound effect.

---

Editor's commentary: - The second paragraph effectively illustrates previous findings but would benefit from offering more interpretations and explaining how these findings built the design or rationale;

Author reply: We revised the manuscript to document interpretations of each prior study on the sight-over-sound effect. Furthermore, the challenges posed by these prior studies, as well as the improvements made in our research, are detailed in the third and fourth paragraphs.

Author action: We have documented the following interpretations for each prior study.

Line 69-72 (Tsay 2013): These findings highlighted that while there is an acknowledgment of sound being the most crucial source of information in the evaluation of music performances, there was also a natural and automatic reliance on visual cues, occurring subconsciously [1].

Line 78-80 (Mehr 2018): These results suggested that the sight-over-sound effect may not be replicable when differences in the skill level of the performances being compared can be adequately judged through auditory perceptio

Attachment Submitted filename: Response to Reviewers_ver2.docx

# Peer review: Decision Letter 1

**DOI:** 10.1371/journal.pone.0321442.r004

**Authors:** Thiago P. Fernandes

1 Jul 2024

<div>PONE-D-23-19525R1Sight-over-sound effect depends on evaluators’ musical experience: An examination using Japanese brass band competition recordingsPLOS ONE

Dear Dr. Samma,

Thank you for submitting your manuscript to PLOS ONE. After careful consideration, we feel that it has merit but does not fully meet PLOS ONE’s publication criteria as it currently stands. Therefore, we invite you to submit a revised version of the manuscript that addresses the points raised during the review process.

==============================

Thank you for your valuable submission. Please respond to all comments and highlight the changes in the ms. Ensure your rebuttal is clear and provide sufficient details so we can streamline. Wishing you success with the study.

==============================

Please submit your revised manuscript by Aug 15 2024 11:59PM. If you will need more time than this to complete your revisions, please reply to this message or contact the journal office at plosone@plos.org . When you're ready to submit your revision, log on to https://www.editorialmanager.com/pone/ and select the 'Submissions Needing Revision' folder to locate your manuscript file.

Please include the following items when submitting your revised manuscript:

- A rebuttal letter that responds to each point raised by the academic editor and reviewer(s). You should upload this letter as a separate file labeled 'Response to Reviewers'.
- A marked-up copy of your manuscript that highlights changes made to the original version. You should upload this as a separate file labeled 'Revised Manuscript with Track Changes'.
- An unmarked version of your revised paper without tracked changes. You should upload this as a separate file labeled 'Manuscript'.

If you would like to make changes to your financial disclosure, please include your updated statement in your cover letter. Guidelines for resubmitting your figure files are available below the reviewer comments at the end of this letter.

If applicable, we recommend that you deposit your laboratory protocols in protocols.io to enhance the reproducibility of your results. Protocols.io assigns your protocol its own identifier (DOI) so that it can be cited independently in the future. For instructions see: https://journals.plos.org/plosone/s/submission-guidelines#loc-laboratory-protocols . Additionally, PLOS ONE offers an option for publishing peer-reviewed Lab Protocol articles, which describe protocols hosted on protocols.io. Read more information on sharing protocols at https://plos.org/protocols?utm_medium=editorial-email&utm_source=authorletters&utm_campaign=protocols .

We look forward to receiving your revised manuscript.

Kind regards,

Thiago P. Fernandes, PhD

Academic Editor

PLOS ONE

Journal Requirements:

Please review your reference list to ensure that it is complete and correct. If you have cited papers that have been retracted, please include the rationale for doing so in the manuscript text, or remove these references and replace them with relevant current references. Any changes to the reference list should be mentioned in the rebuttal letter that accompanies your revised manuscript. If you need to cite a retracted article, indicate the article’s retracted status in the References list and also include a citation and full reference for the retraction notice.

[Note: HTML markup is below. Please do not edit.]

Reviewers' comments:

Reviewer's Responses to Questions

**Comments to the Author**

1. If the authors have adequately addressed your comments raised in a previous round of review and you feel that this manuscript is now acceptable for publication, you may indicate that here to bypass the “Comments to the Author” section, enter your conflict of interest statement in the “Confidential to Editor” section, and submit your "Accept" recommendation.

Reviewer #1: All comments have been addressed

Reviewer #2: (No Response)

Reviewer #4: All comments have been addressed

**********

2. Is the manuscript technically sound, and do the data support the conclusions?

The manuscript must describe a technically sound piece of scientific research with data that supports the conclusions. Experiments must have been conducted rigorously, with appropriate controls, replication, and sample sizes. The conclusions must be drawn appropriately based on the data presented. 

Reviewer #1: Yes

Reviewer #2: Yes

Reviewer #4: Partly

**********

3. Has the statistical analysis been performed appropriately and rigorously? 

Reviewer #1: Yes

Reviewer #2: Yes

Reviewer #4: No

**********

4. Have the authors made all data underlying the findings in their manuscript fully available?

The PLOS Data policy requires authors to make all data underlying the findings described in their manuscript fully available without restriction, with rare exception (please refer to the Data Availability Statement in the manuscript PDF file). The data should be provided as part of the manuscript or its supporting information, or deposited to a public repository. For example, in addition to summary statistics, the data points behind means, medians and variance measures should be available. If there are restrictions on publicly sharing data—e.g. participant privacy or use of data from a third party—those must be specified.

Reviewer #1: Yes

Reviewer #2: No

Reviewer #4: Yes

**********

5. Is the manuscript presented in an intelligible fashion and written in standard English?

PLOS ONE does not copyedit accepted manuscripts, so the language in submitted articles must be clear, correct, and unambiguous. Any typographical or grammatical errors should be corrected at revision, so please note any specific errors here.

Reviewer #1: Yes

Reviewer #2: Yes

Reviewer #4: Yes

**********

6. Review Comments to the Author

Please use the space provided to explain your answers to the questions above. You may also include additional comments for the author, including concerns about dual publication, research ethics, or publication ethics. (Please upload your review as an attachment if it exceeds 20,000 characters)

Reviewer #1: I do appreciate the authors’ thoughtful revision and responses to the comments. Introduction is now good to describe the gap in the current literature and provide rationale for the hypotheses. Revisions of other sections also has improved the quality of the manuscript. I have no other comments.

Reviewer #2: 1. General comments:

In this revised version of the manuscript, the authors have addressed almost all of the issues previously raised.

Notably, they improved the Introduction section, clarified the experimental procedure, reorganized the Results section, and strengthened the Discussion section.

Additionally, they provided intergroup comparisons (along with three additional figures) and discussed some limitations of their study.

Finally, they expanded the Supplementary Information section.

In all, the authors have adequately revised their manuscript which is now publishable in PLoS One, after some minor revisions.

2. Minor revisions:

For page and line numbers, I will refer to the Word document (unless otherwise specified).

2-1. Abstract:

- Page 1, lines 29, 31-33: For the indication of age, please, add “+/- SD” (average age +/- SD = …).

- Page 1, lines 31-33: You should delete the three "n= " at the beginning of the three parts in parentheses.

2-2. Methods:

- Pages 10-11, lines 220-223: The authors' response and Mehr et al's findings suggest that the differences in musical performance skills are basically more related to auditory information than to visual information. The choice of gold prize awarded brass bands only could have partially neutralized the effect of auditory information (almost equivalent levels between groups) in favor of visual information. In this condition, visual information could have become the main cue for achieving the national selection.

- Page 11, lines 241-244: Please clarify whether or not you used the: "Evenly Present Elements" option?

- Page 13, line 273: Please use “Fig. 1” (instead of Figure 1) and reconsider the title (eg : Overview of the Judgment Experiment Procedure). Furthermore, the first sentence is redundant with the title.

2-3. Results:

* Group comparisons by conditions:

- Page 19, lines 398-401: I suggest you rephrase this sentence:

“The percentage of correct responses was: X (median = ...) for BMS, Y (median = ...) for NBMS and Z (median = ...) for NMs.”

- Page 19, lines 413-416: I suggest you rephrase this sentence:

“The percentage of correct responses was: X (median = ...) for BMS, Y (median = ...) for NBMS and Z (median = ...) for NMs.”

- Page 20, lines 430-433: I suggest you rephrase this sentence:

“The percentage of correct responses was: X (median = ...) for BMS, Y (median = ...) for NBMS and Z (median = ...) for NMs.”

2-4. Discussion:

* Evaluator’s musical experience:

- Page 25, lines 548-556: An alternative explanation may be related to the choice of gold prize awarded brass bands only (see above, Comment on pages 10-11, lines 220-223): Subtle acoustic differences (auditory cues) across brass bands may have been noticeable only to the BMs.

- Page 25, lines 557-566: The results of these additional analyzes should be moved to the Results section, in order to only mention the absence of inter-subgroup differences in the Discussion section.

* Limitations:

- Pages 27-28, lines 615-625: An alternative explanation may be related to the choice of regional gold prize awarded brass bands (see above, Comment on pages 10-11, lines 220-223).

- Page 27, line 606: I suggest you to use "it would be important" rather than “it is important”

2-5. References:

- Page 32, line 729: There is a typing error for the reference 21 (“Colledge”).

2-6. Figures:

* Figure 1:

This figure helps clarify the experimental protocol used. However, for each condition, I suggest you shift the "Judgement" rectangle and the word "Response" to the right, so as to make the "Stimuli 3" rectangle more visible. Furthermore, to illustrate the randomization in order of presentation of stimuli, you should alternate the location of the qualified group across sets (in 1st, 2nd or 3rd position).

3. Supplementary information:

* Table S1: For the title, could add “for assessing the normality of data”.

* Supplementary Texts: I think there was a confusion (or an error) in the numbering of the additional texts: S1 Text is missing in the Supplementary Information document and S4 Text is missing in the revised manuscript (only mentioned in the Response to Reviewers). Please correct this point and then check the consistency of the numbering between the manuscript and the supplementary information.

* S1 Figure: The caption should be in normal character (line 26). Please, add a space between “among” and “brass” (line 27).

* S3 Text (S2 Text ?): The title should be in bold (line 29).

* S4 Text (S3 Text ?):

- For the rational, you should mentioned Bouhuys et al, 1995 (Bouhuys AL et al, Induction of depressed and elated mood by music influences the perception of facial emotional expressions in healthy subjects, J Affect Disord 1995)

- For the gender comparison, I suggested assessing the effect of gender (as independent variable) rather than the effect of each gender, on the observed results. If possible, I thank you for carrying out a new analysis, on the same model as the analysis of the two sub-groups of BMs.

- Lines 58 and 64: you should add “the” (among the three conditions)

* S3 Figure:

- The caption should be in normal character (lines 74-76). 26). Please, add a space between “among” and “brass” (line 27).

- Lines 74-75: Please add “according to gender” (for each participant, according to gender)

- Please explain the signification of the dagger mark for VO condition in female participants.

Dr Xavier PERROT, 2024/04/23

Reviewer #4: Thank you for the opportunity to review the manuscript. I believe it is sound and suitable for publication after correcting minor changes.

Below, I provide questions that I believe are pertinent to improving the manuscript.

The introduction covers the justification for the study well, however, I believe the authors could make it more succinct and objective.

I suggest that the authors could describe the methodology better, for example by adding a topic on the location of the study.

there is confusion about the topic of methods and results. The methods should include information that was used in the methodology of the study, the results found after collection should appear in another topic.

I think it would be interesting to provide data characterising the sample for the different groups (e.g. mean and standard deviation for females and males in each group).

With regard to the evaluation procedures, participants could only watch the videos once and could not go back to revisit the stimuli. This can limit the accuracy of judgements, especially if participants are unfamiliar with the procedure.

Was a sample calculated? what were the eligibility criteria for the sample? what inclusion and exclusion criteria were adopted? Who was responsible for recruiting the sample? Make it clear whether the sample was carried out by convenience.

Using more robust tests (e.g. ANCOVA) could help to understand confounding variables, such as the imbalance between female and male gender in the groups analysed. Another suggestion would be to calculate effect sizes (e.g. eta square for Kruskal-Wallis, r for Wilcoxon) to quantify the magnitude of the differences observed between the experimental conditions and groups of participants.

I hope that my considerations will help to improve the final manuscript.

Sincerely.

**********

7. PLOS authors have the option to publish the peer review history of their article (what does this mean? ). If published, this will include your full peer review and any attached files.

If you choose “no”, your identity will remain anonymous but your review may still be made public.

**Do you want your identity to be public for this peer review?** For information about this choice, including consent withdrawal, please see our Privacy Policy .

Reviewer #1: **Yes: ** Kyoung Shin Park

Reviewer #2: **Yes: ** Dr Xavier PERROT

Reviewer #4: No

**********

[NOTE: If reviewer comments were submitted as an attachment file, they will be attached to this email and accessible via the submission site. Please log into your account, locate the manuscript record, and check for the action link "View Attachments". If this link does not appear, there are no attachment files.]

While revising your submission, please upload your figure files to the Preflight Analysis and Conversion Engine (PACE) digital diagnostic tool, https://pacev2.apexcovantage.com/ . PACE helps ensure that figures meet PLOS requirements. To use PACE, you must first register as a user. Registration is free. Then, login and navigate to the UPLOAD tab, where you will find detailed instructions on how to use the tool. If you encounter any issues or have any questions when using PACE, please email PLOS at figures@plos.org . Please note that Supporting Information files do not need this step.

# Author comment: Author response to Decision Letter 2

**DOI:** 10.1371/journal.pone.0321442.r005

30 Aug 2024

Manuscript ID: PONE-D-23-19525

Type: Research Article

Title: Sight-over-sound effect depends on evaluator’s musical experience: An examination using Japanese brass band competition recordings

Author: Samma et al

To: Dr. Thiago P. Fernandes, Academic Editor PLOS ONE

Dear Dr. Fernandes,

Re: Reply to reviewers on PONE-D-23-19525

Thank you very much for your review of our submission, manuscript PONE-D-23-19525, entitled “Replicability of the sight-over-sound effect in the judgement of brass band competitions in Japan,” which we submitted to PLoS ONE.

In this letter, we summarize how we addressed the comments made by the reviewers. We found the reviews to be extremely helpful and have done our best to utilize them to strengthen the paper.

Below, we have provided a detailed explanation of how we addressed each of the comments made by the reviewers.

We greatly appreciate your efforts as an editor of the journal and your contribution to improving our manuscript.

Thank you very much.

Tomohiro Samma, Kazuaki Honda, and Shinya Fujii PhD

Reviewers’ Comments to the Author:

________________________________________

Reviewer #1: I do appreciate the authors’ thoughtful revision and responses to the comments. Introduction is now good to describe the gap in the current literature and provide rationale for the hypotheses. Revisions of other sections also has improved the quality of the manuscript. I have no other comments.

Author reply: We appreciate your comments on the previous version of our manuscript. Your valuable feedback was extremely helpful in improving our paper.

________________________________________

Reviewer #2: 1. General comments:

In this revised version of the manuscript, the authors have addressed almost all of the issues previously raised.

Notably, they improved the Introduction section, clarified the experimental procedure, reorganized the Results section, and strengthened the Discussion section.

Additionally, they provided intergroup comparisons (along with three additional figures) and discussed some limitations of their study.

Finally, they expanded the Supplementary Information section.

In all, the authors have adequately revised their manuscript which is now publishable in PLoS One, after some minor revisions.

Author reply: We sincerely appreciate the specific and constructive comments you provided. Based on your feedback, we have made efforts to further improve our paper. Please see our responses below to each of your comments.

Author action: Please see our actions outlined below.

________________________________________

Reviewer #2: 2. Minor revisions:

For page and line numbers, I will refer to the Word document (unless otherwise specified).

2-1. Abstract:

- Page 1, lines 29, 31-33: For the indication of age, please, add “+/- SD” (average age +/- SD = …).

- Page 1, lines 31-33: You should delete the three "n= " at the beginning of the three parts in parentheses.

Author reply: Please see the corrected sentences highlighted in red below.

Author action:

Line 29: A total of 301 adults (average age ± SD = 34.81 ± 11.71 years) participated…

Line 31-33: Among the participants, 171 were brass band musicians (n = 171; average age ± SD = 33.08 ± 11.57 years), 78 were non-brass band musicians (n = 78; average age ± SD = 35.39 ± 13.03 years), and 52 were non-musicians (n = 52; average age ± SD = 39.15 ± 8.73 years).

________________________________________

Reviewer #2: 2-2. Methods:

- Pages 10-11, lines 220-223: The authors' response and Mehr et al's findings suggest that the differences in musical performance skills are basically more related to auditory information than to visual information. The choice of gold prize awarded brass bands only could have partially neutralized the effect of auditory information (almost equivalent levels between groups) in favor of visual information. In this condition, visual information could have become the main cue for achieving the national selection.

Author reply: Thank you for your thoughtful comment. We considered your perspective to be very important for interpreting our results, and thus we revised our manuscript to add a limitation in the discussion section regarding differences in performance levels.

Author action: Please see the following revised discussion.

Revised discussion (see Pages 28-29, lines 620-636):

“As a third limitation, this study did not test whether the sight-over-sound effect could be observed in cases where there were clear differences in brass band performance levels. The stimuli used in our study were limited to those that received gold prizes at regional brass band competitions, resulting in relatively small differences in performance levels for comparison. This was because a previous study by Mehr et al. [22] reported that the sight-over-sound effect was not observed when comparing stimuli with significant differences in performance skills. Based on the results from this study and Mehr et al.'s findings, it can be assumed that clear differences in musical performance skills are generally more related to auditory information than to visual information. Specifically, it can be assumed that selecting only gold prize-awarded brass bands might have partially neutralized the effect of auditory information (almost equivalent levels between groups) in favor of visual information. In this condition, visual information could have become the main cue for selecting the winner. Additionally, the BMs in this study showed more accurate judgments than the NBMs and NMs because they might have been able to notice the subtle acoustic differences (auditory cues) across the gold-prize brass bands. Thus, further studies are needed to test if the results from this study are replicated under conditions where there are clear differences in brass band performance levels.”

________________________________________

Reviewer #2:

- Page 11, lines 241-244: Please clarify whether or not you used the: "Evenly Present Elements" option?

Author reply: We have added the clarification to the sentence to indicate that we used the “Evenly Present Elements” option.

Author action:

Line 226-227: … the randomization methods with the Evenly Present Elements option implemented in Qualtrics.

________________________________________

Reviewer #2:

- Page 13, line 273: Please use “Fig. 1” (instead of Figure 1) and reconsider the title (eg : Overview of the Judgment Experiment Procedure). Furthermore, the first sentence is redundant with the title.

Author reply: We used “Fig.1” and changed the title. Additionally, we deleted the first sentence from the caption.

Author action:

Line 254-255: Figure 1. Overview the procedure of the judgement experiment procedure.

Example of procedure in the judgement experiment. Each participant performs…

________________________________________

Reviewer #2: 2-3. Results:

* Group comparisons by conditions:

- Page 19, lines 398-401: I suggest you rephrase this sentence:

“The percentage of correct responses was: X (median = ...) for BMS, Y (median = ...) for NBMS and Z (median = ...) for NMs.”

Author reply: We rephrased the sentence as follows:

Author action:

Line 409-411: The percentage of correct responses for BMs was 39.42% (median = 40.0, SD = 15.39, 95% CI: [35.14, 43.71]) for BMs, for NBMs it was 29.37% (median = 30, SD = 12.43, 95% CI: [24.89, 33.86]) for NBMs, and for NMs it was 35.71% (median = 30, SD = 14.69, 95% CI: [29.03, 42.40]) for NMs.

________________________________________

Reviewer #2:

- Page 19, lines 413-416: I suggest you rephrase this sentence:

“The percentage of correct responses was: X (median = ...) for BMS, Y (median = ...) for NBMS and Z (median = ...) for NMs.”

Author reply: We rephrased the sentence as follows:

Author action:

Line 424-426: The percentage of correct responses for BMs was 37.07% (median = 40.0, SD = 18.64, 95 % CI: [32.17 41.97]) for BMs, for NBMs it was 49.52% (median = 50, SD = 16.27, 95 % CI: [42.12 56.93]) for NBMs, and for NMs it was 43.16% (median = 40, SD = 18.19, 95 % CI: [34.84 52.94]) for NMs.

________________________________________

Reviewer #2:

- Page 20, lines 430-433: I suggest you rephrase this sentence:

“The percentage of correct responses was: X (median = ...) for BMS, Y (median = ...) for NBMS and Z (median = ...) for NMs.”

Author reply: We rephrased the sentence as follows:

Author action:

Line 440-442: The percentage of correct responses for BMs was 36.56% (median = 40.0, SD = 13.02, 95 % CI: [33.22 39.89]) for BMs, for NBMs it was 31.60% (median = 30, SD = 16.25, 95 % CI: [24.89 38.31]) for NBMs, and for NMs it was 37.86% (median = 45, SD = 21.93, 95 % CI: [25.21 51.71]) for NMs.

________________________________________

Reviewer #2: 2-4. Discussion:

* Evaluator’s musical experience:

- Page 25, lines 548-556: An alternative explanation may be related to the choice of gold prize awarded brass bands only (see above, Comment on pages 10-11, lines 220-223): Subtle acoustic differences (auditory cues) across brass bands may have been noticeable only to the BMs.

Author reply: Thank you for your valuable suggestion. In response to your comments, we revised the discussion to mention that the results may be related to the choice of gold prize-awarded brass bands only. Specifically, we added a discussion noting that the BMs in this study showed more accurate judgments than the NBMs and NMs because they might have been able to notice the subtle acoustic differences (auditory cues) across the gold-prize brass bands.

Author action: Please see the following revised discussion:

Revised discussion (see Pages 28-29, lines 620-636):

“As a third limitation, this study did not test whether the sight-over-sound effect could be observed in cases where there were clear differences in brass band performance levels. The stimuli used in our study were limited to those that received gold prizes at regional brass band competitions, resulting in relatively small differences in performance levels for comparison. This was because a previous study by Mehr et al. [22] reported that the sight-over-sound effect was not observed when comparing stimuli with significant differences in performance skills. Based on the results from this study and Mehr et al.'s findings, it can be assumed that clear differences in musical performance skills are generally more related to auditory information than to visual information. Specifically, it can be assumed that selecting only gold prize-awarded brass bands might have partially neutralized the effect of auditory information (almost equivalent levels between groups) in favor of visual information. In this condition, visual information could have become the main cue for selecting the winner. Additionally, the BMs in this study showed more accurate judgments than the NBMs and NMs because they might have been able to notice the subtle acoustic differences (auditory cues) across the gold-prize brass bands. Thus, further studies are needed to test if the results from this study are replicated under conditions where there are clear differences in brass band performance levels.”

________________________________________

Reviewer #2:

- Page 25, lines 557-566: The results of these additional analyzes should be moved to the Results section, in order to only mention the absence of inter-subgroup differences in the Discussion section.

Author reply: Considering your comments, we performed a two-way ANOVA to examine the effects of musical experience outside of the brass band. The results from additional analyses have been included in the Results section in the revised manuscript. We decided to delete the results testing the differences among AV, A, and V conditions for each of the BMs with and without other musical experiences because the ANOVA results showed no main effect of musical experience outside of the brass band.

Author action: Please see the following Results and Discussion sections.

Author action: We added following sentences.

Page 19. Line 399-405 in the Results section:

To examine the main effect of musical experience outside of brass bands in BMs, 2 (presence or absence of musical experience outside of brass bands) × 3 (experimental conditions) two-way analysis of variance was conducted. The results indicated that there was no main effect of musical experience (F (1, 0) = 0.0, p = 0.99, ηp2 < 0.0001), nor was there an interaction between gender and experimental conditions (F (2, 714) = 1.42, p = 0.24, ηp2 = 0.02). Therefore, it is considered unlikely that the music experience outside of brass bands influenced the results of the current experiment.

Page 26, Lines 567-569, Discussion section:

Nevertheless, we could not find main effect of musical experience outside of brass bands, suggesting that the effect of having or lacking experience in other musical genres may be minor among BMs.

________________________________________

Reviewer #2:

* Limitations:

- Pages 27-28, lines 615-625: An alternative explanation may be related to the choice of regional gold prize awarded brass bands (see above, Comment on pages 10-11, lines 220-223).

Author reply: Please see our responses above to your comments. We have added limitations to mention that an alternative explanation may be related to the choice of regional gold prize-awarded brass bands.

Author action: Please see the limitation section in the revised discussions.

________________________________________

Reviewer #2:

- Page 27, line 606: I suggest you to use "it would be important" rather than “it is important”

Author reply: We corrected the sentence.

Author action:

Line 610: “Taken together, it would beis important to test…”

________________________________________

Reviewer #2: 2-5. References:

- Page 32, line 729: There is a typing error for the reference 21 (“Colledge”).

Author reply: We corrected the typing error.

Author action: Please see below:

Line 752-753: 21. Scannell DA. Sound Judgment : Auditory – but not Visual – Information Reveals Musical Competition Winners. Boston CollegeColledge, 2014 pp. 1–27. http://hdl.handle.net/2345/3867

________________________________________

Reviewer #2: 2-6. Figures:

* Figure 1:

This figure helps clarify the experimental protocol used. However, for each condition, I suggest you shift the "Judgement" rectangle and the word "Response" to the right, so as to make the "Stimuli 3" rectangle more visible. Furthermore, to illustrate the randomization in order of presentation of stimuli, you should alternate the location of the qualified group across sets (in 1st, 2nd or 3rd position).

Author reply: We have revised the Fig. 1 according to your comments.

Author action:

Please see the revised figure below.

________________________________________

Reviewer #2: 3. Supplementary information:

* Table S1: For the title, could add “for assessing the normality of data”.

Author reply: We added.

Author action:

Supp info, Line 2-3: Table S1. Results of the Shapiro-Wilk test and the values of kurtosis and skewness for assessing the normality of data.

________________________________________

Reviewer #2:

* Supplementary Texts: I think there was a confusion (or an error) in the numbering of the additional texts: S1 Text is missing in the Supplementary Information document and S4 Text is missing in the revised manuscript (only mentioned in the Response to Reviewers). Please correct this point and then check the consistency of the numbering between the manuscript and the supplementary information.

Author reply: We checked the consistency of the numbering between the manuscript and the supplementary information.

Author action: We corrected the numbering of the texts in the supplementary information document.

________________________________________

Reviewer #2:

* S1 Figure: The caption should be in normal character (line 26). Please, add a space between “among” and “brass” (line 27).

Author reply: We added a space.

Author action:

Supp info, Line 29: …brass band competitions among brass band musicians…

________________________________________

Reviewer #2:

* S3 Text (S2 Text ?): The title should be in bold (line 29).

Author reply: We have changed the title to bold.

Author action:

Supp info, Line 31: S23 text. Criteria for

Attachment Submitted filename: Response to Reviewers_round2_final.docx

# Editor report: Decision Letter 2

**DOI:** 10.1371/journal.pone.0321442.r006

**Authors:** Thiago P. Fernandes

6 Sep 2024

<div>PONE-D-23-19525R2Sight-over-sound effect depends on evaluators’ musical experience:

An examination using Japanese brass band competition recordingsPLOS ONE

Dear Dr. Samma,

Thank you for submitting your manuscript to PLOS ONE. After careful consideration, we feel that it has merit but does not fully meet PLOS ONE’s publication criteria as it currently stands. Therefore, we invite you to submit a revised version of the manuscript that addresses the points raised during the review process.

==============================

**Thank you for your valuable submission.**

1) Please double-check grammar (e.g. punctuation and wording);

2) Please double-check refs (e.g. abbreviations, page numbers, and sentence case need to be corrected);

3) Although the title is not concerning, I believe the authors could improve by focusing on the novelty and importance of the study. Objective phrasing, such as highlighting the interaction between musical experience and sensory judgement, would engage a broader audience. For example, using terms like 'auditory-visual' could improve clarity, or elaborating on how musical experience influences sensory perception or performance would be beneficial;

4) Consider refining the Abstract, particularly in terms of language and how the information is presented. The authors are strongly encouraged to make it more engaging, emphasising the novelty and relevance of the research;

- The introductory lines should be revised to highlight the importance of the s-o-s in performance evaluations;

- Consider making the methods and results subsections more distinct;

- Elaborate slightly on why the absence of this effect in brass band musicians and non-musicians is important;

- The abstract could more strongly highlight why this study fills an important gap in understanding multisensory integration in evaluative sensory (assessment?);

- Some phrasing could be simplified to make the Abstract more direct; 

- Additionally, strengthen the Abstract by reinforcing the importance of the findings and making the practical implications of the study more explicit;

5) Expand the discussion on how musical expertise may influence the sight-over-sound effect. Incorporating cognitive and perceptual theories could provide a deeper theoretical framework for Hypothesis 2, especially regarding how experts process auditory and visual cues differently; 

6) The transition between sections, such as from the summary of previous findings to the Introduction of the study, could be made smoother;

7)  Including more references to studies on the influence of visual expressiveness and non-verbal cues on performance judgements would strengthen the arguments;

8) More detail could be provided on how musical training might influence sensory integration in music performance. A brief discussion on perceptual training would also be of interest;

9) The Methods section is a little bit worrying. I'd highly encourage the authors to consider:

- A detailed justification for the chosen sample size is required. Please consider performing a power analysis to ensure the sample size is appropriate;

- More refs and details from prior studies that used similar sample size calculations are necessary;

-  Clarify how the bands were selected (e.g. randomly or based on performance level) and provide further details on the scoring and ranking system used in the competition;

- Differences of demographics - particularly gender -  need to be addressed in the analysis. Please clarify whether gender was included as a covariate or if a gender interaction analysis was conducted to ensure it did not influence the results;

- The reasons for grouping participants (BMs, NBMs, and NMs) need more explanation. Please explain why these cutoff points were used and consider adding more detailed information on groupings;

- While participant recruitment via social media and word-of-mouth is mentioned, there is no discussion of how recruitment bias was controlled. Please provide further elaboration;

- The choice of a 6-second stimulus length needs more explanation. Please explain how the stimuli were randomised across the three conditions (AO, VO, AV) to avoid bias. Also, give more details on how you kept the stimulus presentation consistent, especially since participants may have used different equipment;

10) The inclusion of effect sizes is commendable, but they could be discussed more explicitly in the context of their implications for the findings. This would offer a deeper understanding of the practical significance of the results;

11) The non-significant results for certain conditions (e.g., AO and AV for ALLs, BMs, and NMs) could use more explanation. Consider briefly discussing possible confounding factors or limits, like visual or auditory dominance, in this group;

12) The analysis shows no significant effect of musical experience outside brass bands. However, consider expanding on this. Is there research that explains why outside musical experience won't affect judgment accuracy in this case? Explaining this would strengthen your interpretation;

13) Some aspects for the Results:

- Ensure consistent rounding across all data. For example, round p = 0.021 to p = 0.02 or use three decimal places throughout for precision;

- Format the confidence intervals with commas to clearly separate the lower and upper bounds (e.g. [35.14, 43.71] instead of [35.14 43.71]);

- Present all medians in the same format, with or without decimals;

- Report effect sizes for each pairwise comparison (e.g., AO vs. VO) instead of only for the Kruskal-Wallis test;

- Clarify the main effect of musical experience, as zero degrees of freedom is unusual and may be a typo;

- In cases where the lower bound of an effect size CI is 0, it may indicate no effect. The effect size and its confidence intervals should be checked to ensure they align with the conclusion of significance;

- Consider using Hedges' g for pairwise comparisons and ω² for ANOVA to give more reliable effect size estimates;

14) Expand the discussion to include other visual factors, like performer interactions, that might influence the sight-over-sound effect, not just camera angles;

15) Provide more detailed explanations for NMs' performance. Consider alternatives, like relying on basic visual cues due to a lack of musical knowledge;

16) The study's use of only gold-prize brass bands is noted, but the explanation for why this neutralized auditory information is unconvincing. Expand this reasoning with more evidence or theoretical support;

17) Discuss how a within-subjects design might affect results, considering factors beyond learning effects, like order effects or familiarity with specific pieces;

18) Overall, the discussion needs a clearer structure. Differ sections on limitations, rationale research, and hypotheses separately. Some sections seem repetitive, and transitions are unclear. Strengthen claims about attentional focus, visual dominance, and auditory skills by adding more empirical evidence;

In addition, consider updating refs, including those by Zamm, Bigand, and others that explain how musical training affects motor-cortical plasticity, providing more modern insights than earlier work. Also, provide a clearer summary of the changes made to the ms.

Wishing you success with the study.

==============================

Please submit your revised manuscript by Oct 21 2024 11:59PM. If you will need more time than this to complete your revisions, please reply to this message or contact the journal office at plosone@plos.org . When you're ready to submit your revision, log on to https://www.editorialmanager.com/pone/ and select the 'Submissions Needing Revision' folder to locate your manuscript file.

Please include the following items when submitting your revised manuscript:

- A rebuttal letter that responds to each point raised by the academic editor and reviewer(s). You should upload this letter as a separate file labeled 'Response to Reviewers'.
- A marked-up copy of your manuscript that highlights changes made to the original version. You should upload this as a separate file labeled 'Revised Manuscript with Track Changes'.
- An unmarked version of your revised paper without tracked changes. You should upload this as a separate file labeled 'Manuscript'.

If you would like to make changes to your financial disclosure, please include your updated statement in your cover letter. Guidelines for resubmitting your figure files are available below the reviewer comments at the end of this letter.

If applicable, we recommend that you deposit your laboratory protocols in protocols.io to enhance the reproducibility of your results. Protocols.io assigns your protocol its own identifier (DOI) so that it can be cited independently in the future. For instructions see: https://journals.plos.org/plosone/s/submission-guidelines#loc-laboratory-protocols . Additionally, PLOS ONE offers an option for publishing peer-reviewed Lab Protocol articles, which describe protocols hosted on protocols.io. Read more information on sharing protocols at https://plos.org/protocols?utm_medium=editorial-email&utm_source=authorletters&utm_campaign=protocols .

We look forward to receiving your revised manuscript.

Kind regards,

Thiago P. Fernandes, PhD

Academic Editor

PLOS ONE

**Journal Requirements:**

Please review your reference list to ensure that it is complete and correct. If you have cited papers that have been retracted, please include the rationale for doing so in the manuscript text, or remove these references and replace them with relevant current references. Any changes to the reference list should be mentioned in the rebuttal letter that accompanies your revised manuscript. If you need to cite a retracted article, indicate the article’s retracted status in the References list and also include a citation and full reference for the retraction notice.

[Note: HTML markup is below. Please do not edit.]

[NOTE: If reviewer comments were submitted as an attachment file, they will be attached to this email and accessible via the submission site. Please log into your account, locate the manuscript record, and check for the action link "View Attachments". If this link does not appear, there are no attachment files.]

While revising your submission, please upload your figure files to the Preflight Analysis and Conversion Engine (PACE) digital diagnostic tool, https://pacev2.apexcovantage.com/ . PACE helps ensure that figures meet PLOS requirements. To use PACE, you must first register as a user. Registration is free. Then, login and navigate to the UPLOAD tab, where you will find detailed instructions on how to use the tool. If you encounter any issues or have any questions when using PACE, please email PLOS at figures@plos.org . Please note that Supporting Information files do not need this step.

# Author comment: Author response to Decision Letter 3

**DOI:** 10.1371/journal.pone.0321442.r007

5 Mar 2025

Reviewers’ Comments to the Author:

Editor: Please double-check grammar (e.g. punctuation and wording);

Author reply: Thank you for your comment. We have checked the grammar throughout the manuscript. In addition, we have had them manuscript copyedited by a professional scientific editing service.

Editor: Please double-check refs (e.g. abbreviations, page numbers, and sentence case need to be corrected);

Author reply: Thank you for your comment. We have checked all references and made revisions where required.

Editor: Although the title is not concerning, I believe the authors could improve by focusing on the novelty and importance of the study. Objective phrasing, such as highlighting the interaction between musical experience and sensory judgement, would engage a broader audience. For example, using terms like 'auditory-visual' could improve clarity, or elaborating on how musical experience influences sensory perception or performance would be beneficial;

Author reply: Thank you for your comment. We have revised the title to better highlight the interaction between musical experience and sensory judgment. The title has been revised to: “Sight-over-sound effect depends on interaction between evaluators’ musical experience and auditory-visual integration: An examination using Japanese brass band competition recordings.”

Editor: Consider refining the Abstract, particularly in terms of language and how the information is presented. The authors are strongly encouraged to make it more engaging, emphasizing the novelty and relevance of the research;

- The introductory lines should be revised to highlight the importance of the s-o-s in performance evaluations;

- Consider making the methods and results subsections more distinct;

- Elaborate slightly on why the absence of this effect in brass band musicians and non-musicians is important;

- The abstract could more strongly highlight why this study fills an important gap in understanding multisensory integration in evaluative sensory (assessment?);

- Some phrasing could be simplified to make the Abstract more direct;

- Additionally, strengthen the Abstract by reinforcing the importance of the findings and making the practical implications of the study more explicit;

Author reply: Thank you for your detailed feedback on refining the Abstract. We have revised the introduction to emphasize the importance of the sight-over-sound effect in performance evaluation (lines 23-25). Additionally, we have clearly stated the objective of this study in relation to previous research and highlighted its novelty (lines 25-28). The methods and results sections have been clarified by distinguishing the subgroups more explicitly (lines 28-34), and we have discussed the significance of the absence of the sight-over-sound effect in BMs and NMs (lines 34-38). Furthermore, we have explicitly stated that our findings contribute to filling a critical gap in multisensory integration research by elucidating how differences in musical experience influence evaluative processes across sensory modalities (lines 38-40).

Editor: Expand the discussion on how musical expertise may influence the sight-over-sound effect. Incorporating cognitive and perceptual theories could provide a deeper theoretical framework for Hypothesis 2, especially regarding how experts process auditory and visual cues differently;

Author reply: Thank you for your valuable suggestion. Based on previous studies indicating that musicians exhibit greater selective attention to auditory information than non-musicians [48, 49], we have noted that BMs, with their brass band training experience, may possess superior selective auditory attention abilities compared to NBMs and NMs.

Editor: The transition between sections, such as from the summary of previous findings to the Introduction of the study, could be made smoother;

Author reply: Thank you for your comment. We have revised the transition between sections throughout the manuscript. In particular, we have rewritten the transition from the summary of previous findings to the aim of the present study to improve the flow of the Introduction (lines 140–146).

Editor: Including more references to studies on the influence of visual expressiveness and non-verbal cues on performance judgements would strengthen the arguments;

Author reply: Thank you for your suggestion. We added more references to studies on the influence of visual expressiveness and non-verbal cues on performance judgements [3, 4, 8, 9, 14].

Editor: More detail could be provided on how musical training might influence sensory integration in music performance. A brief discussion on perceptual training would also be of interest;

Author reply: Thank you for your comment. We have added a discussion citing previous studies reporting that musicians possess advanced auditory processing abilities [40,41] and demonstrate higher accuracy in pitch [42-44] and rhythm perception [45-47] compared to non-musicians. Additionally, we have noted that auditory information processing developed through musical training may influence the sight-over-sound effect.

Editor: The Methods section is a little bit worrying. I'd highly encourage the authors to consider:

- A detailed justification for the chosen sample size is required. Please consider performing a power analysis to ensure the sample size is appropriate;

- More refs and details from prior studies that used similar sample size calculations are necessary;

Author reply: Thank you for your valuable feedback. We have added a Statistical Power subsection to include the results of our power analysis (lines 195-200). Additionally, we have provided details on sample size calculations and effect sizes from previous studies.

Editor:

- Clarify how the bands were selected (e.g. randomly or based on performance level) and provide further details on the scoring and ranking system used in the competition;

Author reply: Thank you for your valuable comments. We have clarified that the brass bands were selected based on their performance level (lines 225-237) and have added a detailed explanation of the scoring and ranking system used in the competition (lines 220-224).

Editor:

- Differences of demographics - particularly gender - need to be addressed in the analysis. Please clarify whether gender was included as a covariate or if a gender interaction analysis was conducted to ensure it did not influence the results;

Author reply: Thank you for your insightful comment. We have decided to move the explanation about the impact of gender differences on the sight-over-sound effect, previously included in the Supporting Information, to the Results section of the manuscript (lines 483-499). We conducted a two-way ANOVA with sex and experimental condition as factors, as well as an ANCOVA with accuracy rate as the dependent variable, experimental condition as the independent variable, and sex as a covariate. In both analyses, we confirmed that participants’ sex had no effect on the results of this study.

Editor:

- The reasons for grouping participants (BMs, NBMs, and NMs) need more explanation. Please explain why these cutoff points were used and consider adding more detailed information on groupings;

Author reply: Thank you for your valuable feedback. In this study, we hypothesized that experience with a specific musical genre could influence the sight-over-sound effect. Therefore, we categorized participants into three groups: BMs, who had direct experience with brass band music; NBMs, who had general musical experience but no direct brass band experience; and NMs, who had no formal musical training beyond compulsory education. This classification was implemented to examine the impact of brass band experience on performance evaluation. We have included this explanation in the Participant Grouping subsection (lines 207-215).

Editor:

- While participant recruitment via social media and word-of-mouth is mentioned, there is no discussion of how recruitment bias was controlled. Please provide further elaboration;

Author reply: Thank you for your comment. We have added a discussion on the limitations of recruitment bias in online experiments as the first limitation of this study (lines 676-678).

Editor:

- The choice of a 6-second stimulus length needs more explanation. Please explain how the stimuli were randomized across the three conditions (AO, VO, AV) to avoid bias. Also, give more details on how you kept the stimulus presentation consistent, especially since participants may have used different equipment;

Author reply: Thank you for your comment. We have explained the rationale for selecting a 6-second stimulus length by referencing Ambady et al.‘s “thin-slices” research [51] (lines 242-246). Additionally, we have provided details on the method used to randomize the stimuli across conditions (lines 252-256). Furthermore, we have acknowledged the potential variation in participants’ equipment as the first limitation of this study (lines 678-681).

Editor:

Some aspects for the Results:

- Ensure consistent rounding across all data. For example, round p = 0.021 to p = 0.02 or use three decimal places throughout for precision;* Table S1: For the title, could add “for assessing the normality of data”.

Author reply: Thank you for your comment. We have rounded all the data to two decimal places (e.g. p = 0.07 instead of p = 0.067).

Editor:

- Format the confidence intervals with commas to clearly separate the lower and upper bounds (e.g. [35.14, 43.71] instead of [35.14 43.71]);

Author reply: Thank you for your comment. We have formatted the confidence intervals with commas to clearly separate the lower and upper bounds.

Editor:

- Present all medians in the same format, with or without decimals;

Author reply: Thank you for your valuable comment. We formatted all medians without decimals (e.g. 40 instead of 40.0)

Editor:

- Report effect sizes for each pairwise comparison (e.g., AO vs. VO) instead of only for the Kruskal-Wallis test;

Author reply: Thank you for your valuable comment. We added effect sizes for each pairwise comparison (lines 401-402; 445-446; 458-461).

Editor:

- Clarify the main effect of musical experience, as zero degrees of freedom is unusual and may be a typo;

Author reply: Thank you for your valuable comment. It was a typo and we revised the result (line 485).

Editor:

- In cases where the lower bound of an effect size CI is 0, it may indicate no effect. The effect size and its confidence intervals should be checked to ensure they align with the conclusion of significance;

Author reply: Thank you for your suggestion. We have added that the lower bound of the effect size CI is 0, indicating the possibility that this result may not reflect a meaningful effect (lines 460-461).

Editor:

- Consider using Hedges' g for pairwise comparisons and ω² for ANOVA to give more reliable effect size estimates;

Author reply: Thank you for your comment. We use ω² for ANOVA (lines 484-486).

Editor:

The inclusion of effect sizes is commendable, but they could be discussed more explicitly in the context of their implications for the findings. This would offer a deeper understanding of the practical significance of the results;

Author reply: Thank you for your suggestion. We have noted that the effect sizes calculated for the comparisons between conditions in NBMs were relatively large (η2 = 0.53), indicating that the observed differences in NBMs were meaningful (lines 512-516).

Editor:

The non-significant results for certain conditions (e.g., AO and AV for ALLs, BMs, and NMs) could use more explanation. Consider briefly discussing possible confounding factors or limits, like visual or auditory dominance, in this group;

Author reply: Thank you for your suggestion. We have noted as the fourth limitation of this study, as well as an open question for future research, that the accuracy rates in the AV condition did not surpass those in the AO or VO conditions. Based on previous studies suggesting that the combination of auditory and visual information allows for a more accurate evaluation of a performer’s emotional intent [80,81], we have expanded the discussion accordingly (lines 699-709).

Editor:

The analysis shows no significant effect of musical experience outside brass bands. However, consider expanding on this. Is there research that explains why outside musical experience won't affect judgment accuracy in this case? Explaining this would strengthen your interpretation;

Author reply: Thank you for your suggestion. We have expanded the discussion to strengthen the validity of our interpretation by citing previous studies that report how experience with specific music genres shapes neural responses to musical features [63] and influences sensorimotor synchronization and timing perception [64] (lines 615-622).

Editor:

Expand the discussion to include other visual factors, like performer interactions, that might influence the sight-over-sound effect, not just camera angles;

Author reply: Thank you for your comment. As suggested, we have included a discussion on the potential influence of visual factors, such as performer interactions, on the sight-over-sound effect (lines 596-606).

Editor:

Provide more detailed explanations for NMs' performance. Consider alternatives, like relying on basic visual cues due to a lack of musical knowledge;

Author reply: Thank you for your comment. As you suggested, we have added a statement discussing the possibility that NMs relied on basic visual cues for evaluation due to their lack of musical knowledge (lines 668-672). Additionally, we have included citations to relevant previous studies.

Editor:

The study's use of only gold-prize brass bands is noted, but the explanation for why this neutralized auditory information is unconvincing. Expand this reasoning with more evidence or theoretical support;

Author reply: Thank you for your valuable comment. In our study, we aimed to minimize differences in performance level (auditory information) when selecting stimuli. However, since brass band competition rankings are not publicly disclosed, it is difficult to objectively confirm that performance-level neutralization was fully achieved. Nevertheless, the chance-level accuracy observed in the auditory-only condition (e.g., ALLs data) suggests that participants were unable to systematically identify performance differences based on auditory information alone, implying that the influence of auditory cues was effectively minimized. We have added this discussion as the fifth limitation of our study (lines 710-721).

Editor:

Discuss how a within-subjects design might affect results, considering factors beyond learning effects, like order effects or familiarity with specific pieces;

Author reply: Thank you for your comment. We have noted the lack of a within-subject design as the second limitation of this study (lines 685-692). In that section, we have added a statement highlighting the need to consider order effects and familiarity with specific musical pieces (lines 687-690).

Editor:

Overall, the discussion needs a clearer structure. Differ sections on limitations, rationale research, and hypotheses separately. Some sections seem repetitive, and transitions are unclear. Strengthen claims about attentional focus, visual dominance, and auditory skills by adding more empirical evidence;

Author reply: Thank you for your suggestions. Given that the study specifically involves an investigation into the replicability of the sight-over-sound effect, we have structured the Discussion to focus on the differences and similarities between our findings and those of previous studies. As well as subsections discussing these differences and similarities, we have included separate limitations and conclusions subsections to clearly structure the Discussion section. We have also added text (with appropriate citations) to strengthen the claims about attentional focus (lines 580-581), visual dominance (lines 660-666), and auditory skills (lines 635-641).

Editor:

In addition, consider updating refs, including those by Zamm, Bigand, and others that explain how musical training affects motor-cortical pla

Attachment Submitted filename: Response to Reviewers_round3.docx

# Editor report: Decision Letter 3

**DOI:** 10.1371/journal.pone.0321442.r008

**Authors:** Thiago P. Fernandes

7 Mar 2025

Sight-over-sound effect depends on interaction between evaluators’ musical experience and auditory-visual integration:

An examination using Japanese brass band competition recordings

PONE-D-23-19525R3

Dear Dr. Samma,

We’re pleased to inform you that your manuscript has been judged scientifically suitable for publication and will be formally accepted for publication once it meets all outstanding technical requirements.

Within one week, you’ll receive an e-mail detailing the required amendments. When these have been addressed, you’ll receive a formal acceptance letter and your manuscript will be scheduled for publication.

An invoice will be generated when your article is formally accepted. Please note, if your institution has a publishing partnership with PLOS and your article meets the relevant criteria, all or part of your publication costs will be covered. Please make sure your user information is up-to-date by logging into Editorial Manager at Editorial Manager®  and clicking the ‘Update My Information' link at the top of the page. If you have any questions relating to publication charges, please contact our Author Billing department directly at authorbilling@plos.org.

If your institution or institutions have a press office, please notify them about your upcoming paper to help maximize its impact. If they’ll be preparing press materials, please inform our press team as soon as possible -- no later than 48 hours after receiving the formal acceptance. Your manuscript will remain under strict press embargo until 2 pm Eastern Time on the date of publication. For more information, please contact onepress@plos.org.

Kind regards,

Thiago P. Fernandes, PhD

Academic Editor

PLOS ONE

Additional Editor Comments (optional):

Thank you for your thoughtful and careful edits.

Wishing you success with the study.

Reviewers' comments:

# Editor report: Acceptance letter

**DOI:** 10.1371/journal.pone.0321442.r009

**Authors:** Thiago P. Fernandes

PONE-D-23-19525R3

PLOS ONE

Dear Dr. Samma,

I'm pleased to inform you that your manuscript has been deemed suitable for publication in PLOS ONE. Congratulations! Your manuscript is now being handed over to our production team.

At this stage, our production department will prepare your paper for publication. This includes ensuring the following:

* All references, tables, and figures are properly cited

* All relevant supporting information is included in the manuscript submission,

* There are no issues that prevent the paper from being properly typeset

If revisions are needed, the production department will contact you directly to resolve them. If no revisions are needed, you will receive an email when the publication date has been set. At this time, we do not offer pre-publication proofs to authors during production of the accepted work. Please keep in mind that we are working through a large volume of accepted articles, so please give us a few weeks to review your paper and let you know the next and final steps.

Lastly, if your institution or institutions have a press office, please let them know about your upcoming paper now to help maximize its impact. If they'll be preparing press materials, please inform our press team within the next 48 hours. Your manuscript will remain under strict press embargo until 2 pm Eastern Time on the date of publication. For more information, please contact onepress@plos.org.

If we can help with anything else, please email us at customercare@plos.org.

Thank you for submitting your work to PLOS ONE and supporting open access.

Kind regards,

PLOS ONE Editorial Office Staff

on behalf of

Dr. Thiago P. Fernandes

Academic Editor

PLOS ONE
